# Ion-Imprinted Polymeric Materials for Selective Adsorption of Heavy Metal Ions from Aqueous Solution

**DOI:** 10.3390/molecules28062798

**Published:** 2023-03-20

**Authors:** Maria Marinela Lazar, Claudiu-Augustin Ghiorghita, Ecaterina Stela Dragan, Doina Humelnicu, Maria Valentina Dinu

**Affiliations:** 1 Department of Functional Polymers, Petru Poni Institute of Macromolecular Chemistry, Grigore Ghica Voda Alley 41 A, 700487 Iasi, Romania; 2 Faculty of Chemistry, Alexandru Ioan Cuza University of Iasi, Carol I Bd. 11, 700506 Iasi, Romania

**Keywords:** adsorption, ion-imprinted polymers, heavy metal ions, selectivity, wastewater treatment

## Abstract

The introduction of selective recognition sites toward certain heavy metal ions (HMIs) is a great challenge, which has a major role when the separation of species with similar physicochemical features is considered. In this context, ion-imprinted polymers (IIPs) developed based on the principle of molecular imprinting methodology, have emerged as an innovative solution. Recent advances in IIPs have shown that they exhibit higher selectivity coefficients than non-imprinted ones, which could support a large range of environmental applications starting from extraction and monitoring of HMIs to their detection and quantification. This review will emphasize the application of IIPs for selective removal of transition metal ions (including HMIs, precious metal ions, radionuclides, and rare earth metal ions) from aqueous solution by critically analyzing the most relevant literature studies from the last decade. In the first part of this review, the chemical components of IIPs, the main ion-imprinting technologies as well as the characterization methods used to evaluate the binding properties are briefly presented. In the second part, synthesis parameters, adsorption performance, and a descriptive analysis of solid phase extraction of heavy metal ions by various IIPs are provided.

## 1. Introduction

The removal and monitoring of heavy metal ions (HMIs) from/in aqueous environments are continuously in demand because of their high persistence in ecosystems, ability to accumulate in living organisms, and high toxicity [1,2]. In the past few years, numerous attempts have been reported to minimize the harmful impact of HMIs [3,4,5,6,7,8]. In this regard, various water treatment technologies, including liquid–liquid extraction, solid–liquid extraction (or solid-phase extraction, SPE) [3,4,5,6,8], coagulation/flocculation [3,4,5,9], chemical precipitation [3,4,5], oxidation [3,4,5], bioremediation [3,4,5,10], electrochemical treatment [3,4,5], and membrane filtration technologies [3,4,5,11] have received increasing attention.

Sorbents bearing various chelating ligands were prepared by two main strategies: (i) post-functionalization of commercially available [12,13,14,15,16] or homemade [12,17,18,19,20] cross-linked copolymers; (ii) “bottom-up” design of specialized materials by the free radical polymerization of monomers endowed with adequate pre-existing heteroatomic moieties, such as –COOH, –OH, –SH, –NH_2_, =NH, –N=, –SO_3_H, and –PO_3_H, in the presence of a cross-linker [18,20,21], or by the condensation of monomers containing a high density of reactive functional groups [12,22]. Even so, all the sorbents described above have weak selectivity.

Although selective recognition is a challenging issue for both wastewater treatment and HMIs monitoring, it is laborious to accomplish with the above-mentioned sorbents. The development of highly selective sorbents for the purification of wastewater and recovery of HMIs requires a multitude of recognition sites and tailored morphologies [23]. To achieve these goals, molecular imprinting was developed as an emerging technology to create selective recognition sites in a polymeric matrix [24,25,26,27]. Molecularly imprinted polymers (MIPs) are well-known as the synthetic mimics of biological recognition systems, such as antibody–antigen, enzyme–inhibitor, and receptor–effector [26,27]. The operation principle of MIPs is based on a “lock and key” mechanism similar to that of an antibody that is capable of recognizing a specific antigen in the biological system. Compared with natural receptors, MIPs have the advantages of long-term stability, low cost (cheap starting materials), and better control of the response (stimuli-responsive MIPs) [6,28]. Consequently, MIPs have found a fast-growing trend in analytical chemistry (chromatography, SPE, and chiral separations) as well as in medicine, sensing, and catalysis [24,25,26,27,28].

**Figure 1:**
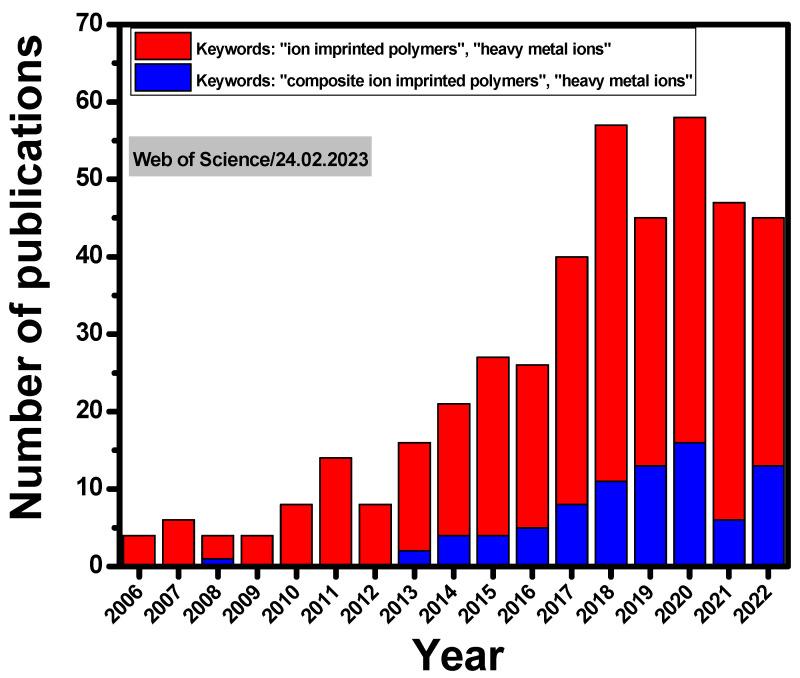
A timeline showing the number of publications on IIPs and composite IIPs in the last 16 years indexed by the Web of Science™ database.

The concept of MIPs has also been considered to design ion-imprinted polymers (IIPs). The first study on IIPs was published by Nishide et al., in 1976 [29], who synthesized IIPs by cross-linking poly(4-vinylpiridine) [P(4-VP)] with 1,4-dibromobutane in the presence of a metal ion as a template (such as Co(II), Cu(II), Fe(III), Hg(II), Ni(II), or Zn(II)). Nevertheless, a considerable interest in the development of this field is more recently visible as the number of publications has steadily increased year-by-year (Figure 1). In addition, since 2013, the preparation of composite IIPs for the selective separation of HMIs has also received growing attention from the scientific community (Figure 1).

**Figure 2:**
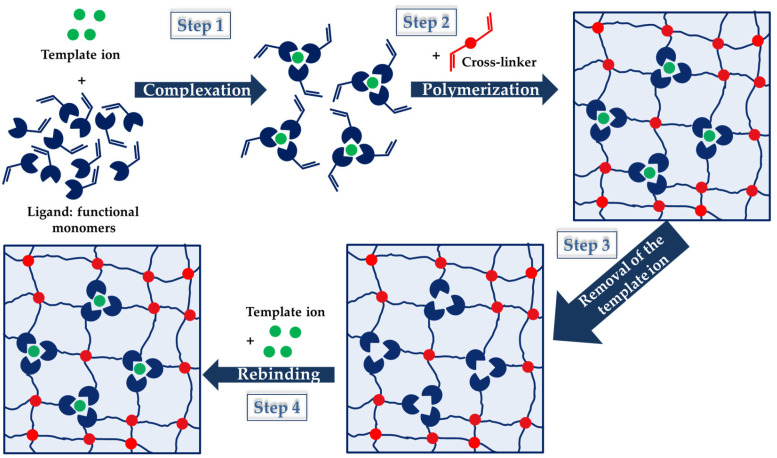
A schematic representation of the steps employed within the IIPs synthesis. The imprinting process is presented using functional monomers as ligand.

IIPs exhibit similar features as MIPs, the main difference being related to the specific recognition sites that are inorganic ions after the imprinting process in the case of former ones [30,31,32,33,34,35,36,37,38]. The strategies applied to prepare MIPs have been also adapted to the synthesis of IIPs and they will be briefly described in the next section of this review. However, all of the approaches follow a similar outline, as can be seen in Figure 2, where the imprinting process using functional monomers as a ligand is depicted.

As Figure 2 shows, the IIP synthesis is based on three steps: (i) in the first step, a complex between metal ion (template) and ligand functional groups of the host (monomers) is generated by non-covalent interactions (chelation, electrostatic interactions, and hydrophobic interactions); (ii) in the second step, the polymerization of this complex and the stabilization of the binding cavities are achieved using a bi-functional monomer (cross-linker); (iii) in the third step, the template ion is leached from the copolymer host using adequate compounds, and thus specific cavities available for selective rebinding are created [30,31,32,33,34,35,36,37,38]. To prove the existence of pre-organized recognition sites, in the fourth step (Figure 2), the IIPs are exposed to the template ion, and the imprinted cavities are thus selectively filled by the target metal ion [38]. Lately, driven by the intrinsic features of polysaccharides, the cross-linking of natural polymers, including chitosan (CS), cellulose (CEL), and alginate (ALG), carrying metal-binding groups has been the main approach used to engineer IIPs [33,34,37,38].

**Figure 3:**
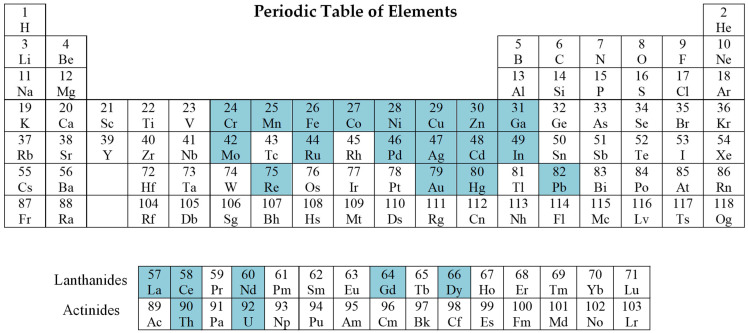
Periodic table of elements. The target metal ions discussed in this work are highlighted in blue.

Several reviews have been published on the technologies used to synthesize IIPs for applications in SPE and sensing [30,31,32,33,34,35,36,37,38,39]. For instance, Hande et al. [31] presented the IIP synthesis methodologies for HMIs’ preconcentration and sensing, and recent advances in IIPs for specific radionuclides can be found in the publication of Kusumkar et al. [35], whereas Chen et al., reported on computational simulation of IIPs synthesis [39]. Further, Erdem et al. [32] described the synthesis and application of IIPs based only on synthetic polymers, while the construction of natural IIPs and their applications in water treatment was described by Li and Yang [33]. The present review will focus on the application of IIPs based on synthetic and natural polymers, or a combination of them for cationic or anionic species recognition, emphasizing mainly the papers published since 2011. Figure 3 presents the periodic table where the elements used as template ions considered in this work are highlighted. The objective of this review is to give updated information and in-depth perspectives on the application of IIPs materials for the selective extraction of various transition metal ions (TMI) from aqueous environments. This review will also provide an overall view on the efficiency of the selective extraction of different IIPs materials obtained by various approaches.

## 2. Important Features on IIPs’ Synthesis and Evaluation of Selective Binding Properties

### 2.1. Main Components of IIPs

The main components of IIPs, besides the target ion that is the template, are generally the functional monomers, cross-linkers, solvents (porogens), and initiators. The selection of these compounds is crucial for the preparation of IIPs with high selectivity and specificity [38].

#### 2.1.1. Templates

As presented above for the preparation of IIPs, metal ions are usually the templates. The selectivity of IIPs is diminished when a target metal ion exhibits similar physicochemical characteristics to the competing ones. Thus, during the preparation process, the template ion and its ligand are adequately chosen to improve IIP selectivity [38].

#### 2.1.2. Functional Monomers and Polymers

In the selection of functional monomers, the following aspects should be considered: (i) the monomer should contain functional moieties that can bind the template ion; (ii) the monomer should be stable during the polymerization process; and (iii) the monomer should not contain ligand groups that inhibit polymerization. Commercial monomers that contain a vinyl group such as methacrylic acid (MAA), 4-vinylpyridine (4-VP), 1-vinylimidazole (1-VI), acrylamide (AAm), and acrylic acid (AA) were mainly employed as ligands to obtain IIPs [30,31,32,33,34,35,36,37,38]. However, these monomers have low efficiency in terms of selectivity. When the ligands did not contain vinyl groups, they were modified to include polymerizable moieties, as in the case of *N*-methacryloyl-L-histidine (MAH) and crown ethers [30,31,32,33,34,35,36,37,38]. Furthermore, the introduction of the trapping approach by Rao et al. [30] has allowed the physical entrapment of various non-vinylated ligands (such as diphenylcarbazide (DPC), dithizone (DZ), and 8-hydroxyquinoline (8-HQ)) within polymer networks generated by copolymerization of vinylated ligands with a cross-linker [36,37].

The polymers used to prepare IIPs should contain metal-binding groups. Besides the first work, in which P4-VP was used to prepare IIPs [39], the use of other polymers has also been reported. For instance, Kabanov et al., prepared a copolymer of diethyl vinyl phosphonate and AA by cross-linking with methylenebisacrylamide (MBAAm) in the presence of HMIs [40], and Ohga et al., prepared reusable sorbents by cross-linking metal-complexed CS with chloromethyloxirane for the separation of Cd(II) from Cu(II) and Hg(II) [41]. In the last decade, CS as a functional linear polymer has mainly been used due to its great abundance in nature, non-toxicity, biocompatibility, and biodegradability [33,34]. Besides CS, the use of ALG and CEL derivatives has also been reported [33].

#### 2.1.3. Cross-Linkers

Cross-linking agents are used to stabilize the entire network. The amount and the nature of the cross-linking agent have a significant impact on the sorption performance of IIPs. Cross-linking agents can be classified into two categories, namely, those comprising vinyl groups that can react with functional monomers (e.g., MBAAm, ethylene glycol dimethacrylate (EGDMA), trimethylolpropane trimethacrylate (TMPTMA), poly(ethylene glycol)diacrylate (PEGDA), and divinylbenzene (DVB)) and those that are only used to cross-link linear or hyper-branched polymers (e.g., glutaraldehyde (GA) and epichlorohydrin (ECH)) [36,37,38]. Insufficient cross-linking agents could lead to IIP with reduced mechanical stability and with randomly distributed recognition sites that can affect the selectivity toward the template ion [38]. On the other hand, the introduction of a high amount of cross-linking agent could generate IIPs with rigid networks, which could reduce the mass transfer performance, chains flexibility, and the number of recognition sites per unit of mass [37,38].

#### 2.1.4. Initiators

Peroxides and azo compounds are commonly used initiators. The compound azobisisobutyronitrile (AIBN) is widely used as an initiator due to its mild fragmentation conditions. The removal of dissolved oxygen from the polymerization system is a key step because oxygen inhibits free radical polymerization. This can be generally achieved using vacuum extraction, ultrasound, nitrogen, or argon purging [38].

#### 2.1.5. Porogens to Generate Porous 3D Structure within IIPs

In IIP synthesis, the solvent provides the reaction environment for the polymerization reaction. The solvent is responsible for both the dissolution of the reagents required for polymerization (e.g., template ions, functional monomers, cross-linking agents, and initiators) and porosity. The polarity and the dielectric constant are important characteristics of the solvent that directly influence the thermodynamic features of IIPs. These thermodynamic properties do not only affect the porous polymer structures but also play key roles in the preparation of organized/precise imprinting sites. Therefore, the choice of solvent is directly connected to the physicochemical parameters of the template ions, functional monomers, and cross-linking agents. Toluene, methanol, DMF, DMSO, acetonitrile, CHCl_3_, CH_3_COOH, and dichloroethane are solvents commonly used [37,38].

#### 2.1.6. Reagents to Leach out the Template Ion

To leach out the template ion, the most common practice is to wash the IIPs with a strong acid such as HCl, H_2_SO_4_, or HNO_3_ [37]. However, they can exhibit a higher affinity for the ligand than for the target ion. Thus, when some components of the IIP (such as functional monomers) are unstable in acidic conditions, the use of ethylenediaminetetraacetic acid (EDTA) and thiourea as chelating agents is recommended [37].

### 2.2. Strategies to Prepare IIPs Materials

**Figure 4:**
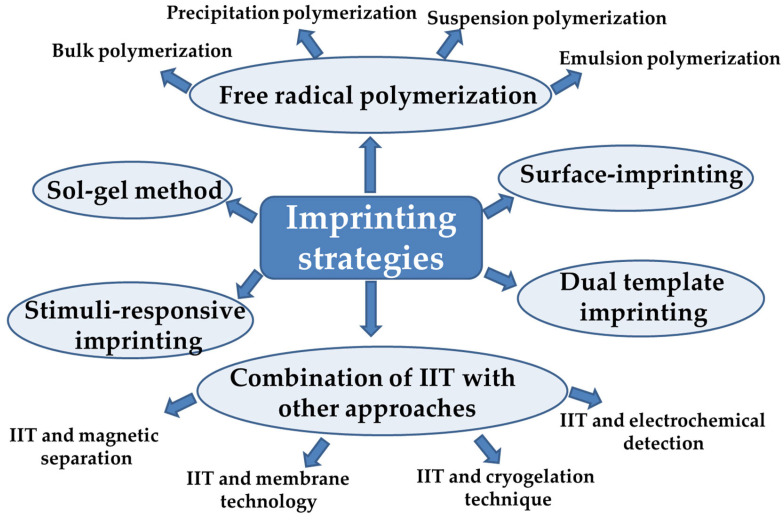
Strategies applied to prepare IIPs materials (IIT is the abbreviation for ion imprinting technology).

IIPs can be obtained using multiple preparation methods (Figure 4). The most commonly used approach is free radical polymerization, which includes bulk, precipitation, suspension, and emulsion polymerization methods. The imprinting by polymerization techniques has been deeply described in the publications of Branger et al. [36,37] and Zhou et al. [38], and thus they will be just briefly presented. To overcome the disadvantages of conventional IIPs, a plethora of technologies have been further promoted such as surface imprinting, stimuli-responsive imprinting, and dual or multiple-component imprinting strategies [38].

#### 2.2.1. Bulk Polymerization

Bulk polymerization is the earliest and simplest approach to prepare IIPs that do not need special equipment [37,38]. The template ions, functional monomers, cross-linking agents, and initiators are usually dissolved in good solvents according to an optimum ratio. To obtain IIP particles with a certain dimension, the bulk materials are crushed, ground, and sieved. This procedure can have a major disadvantage since the grinding, crushing, and sieving operations can destroy some binding sites, thus leading to a significant decrease in the yield of the process [37]. In addition, the irregular shape and size of the IIP particles after grounding are an inconvenience for many chromatographic and separation applications. Due to these limitations, many studies have been focused to prepare IIPs directly as beads using homogeneous or heterogeneous polymerization.

#### 2.2.2. Precipitation Polymerization

The reaction mixture consists of a single phase, composed of monomers, ion templates, and initiators, all dissolved in various pore-forming agents. The polymerization is initiated in a homogeneous solution. This technique requires that the obtained polymer be insoluble in the reaction mixture. Once polymerization starts, the first oligomers and insoluble polymer cores start to form [37,38]. The former remains dissolved in the solvent (porogens), while the nuclei precipitate and continuously grow by adding other monomers and oligomers from the continuous phase [37,38]. The precipitation polymerization approach is the second most used process after bulk polymerization to prepare IIP in form of particles. This technique allows for good control of the particle dimensions, whereby IIPs in the micrometric or nanometric range can be synthesized by optimizing the monomers to solvent ratio and the stirring of the polymerization mixture. However, one limitation of this method is the use of an excess of porogen, which results in multiple purification steps for its removal. Moreover, the template ion can be easily leached out and some binding sites can be wiped out since fewer cross-linked polymer networks are generated by this technique.

#### 2.2.3. Suspension Polymerization

In suspension polymerization, the synthesis of IIPs occurs in the dispersed phase, which contains template ions, functional monomers, porogens, and initiators that can be regarded as a set of many micro-reactors for carrying out local bulk polymerization [37,38]. The process requires constant mechanical stirring to keep particles suspended in the continuous phase containing the stabilizers. The suspension polymerization technique typically allows the preparation of IIPs with sizes in the micrometric range (250–550 μm). The porosity of IIPs prepared by suspension polymerization can be adjusted by porogenic solvents. To avoid the passage of the template ion from the dispersed organic phase to the aqueous dispersing phase, the use of inverse suspension polymerization was proposed. In this process, the dispersion phase is a mineral oil [37,38].

#### 2.2.4. Emulsion Polymerization

For emulsion polymerization, the continuous phase is usually water. In this case, the hydrophobic monomer was dispersed in water using an oil-in-water emulsifier. The continuous phase contains the initiators that produce free radicals [37,38]. As the reaction proceeds, a growing oil–water interface is generated due to the formation of new polymer cores and their growth. To impede the agglomeration of the growing nuclei, a stabilizing agent is added. Nevertheless, this also represents a drawback since additional purification steps to remove the surfactant from IIP particles are necessary. For this technology, the reverse phase emulsion (water-in-oil) can also be used. With this method the size of the IIPs can be well controlled, allowing for the preparation of uniform microspheres and nanospheres [37,38].

#### 2.2.5. Sol–Gel Method

Sol–gel method can generate stable and uniform IIPs under mild synthesis conditions. The template ions are introduced into the inorganic network through gel interaction. Organic–inorganic hybrid materials with different shapes, sizes, specific functional moieties, and affinity can be prepared by the sol–gel technique [37,38]. The IIPs prepared by sol–gel approach exhibit greater thermal and chemical stability than those obtained with conventional imprinting methods [37].

#### 2.2.6. Surface-Imprinting

Conventional imprinting methods for IIPs may result in multiple shortcomings including excessive template ions entrapping depth, difficult leaching, and low regeneration level, poor affinity of the imprinting sites for the target species, reduced mass transfer effect. These drawbacks can be solved by surface-imprinting polymerization technology. The main advantage of the surface-imprinting approach consists in the enhancement of the adsorption efficiency and selectivity through the generation of the recognition sites on the large specific surface area of the carrier [36,37,38]. For surface-imprinting polymerization, identifying carrier materials with stable properties, large specific surface areas, and low price is crucial. In recent years, various IIPs have been fabricated by means of surface-imprinting technology, with supports including activated carbon, silicon materials, magnetic materials, and clay minerals [30,31,32,33,34,35,36,37,38]. Moreover, this technique has been widely investigated to design surface-imprinted materials consisting of various natural polymers with improved selectivity and specificity [33,34]. However, it should be pointed out that there are some shortcomings in using natural polymers such as the impossibility to include all appropriate functional moieties on natural polymers and their lack of structural stability under harsh conditions [33].

#### 2.2.7. Other Imprinting Technologies

With the development of IIT, strategies based on dual or multiple functional monomers and dual or multiple template ions have attracted increasing attention [36]. As IIT has been developed, many scholars have investigated its combination with other technologies, such as magnetic separation technology, membrane technology, and electrochemical detection technology [30,31,32,33,34,35,36,37,38]. In the following sections, the preparation of IIPs using the combination of IIT and of other approaches will be described.

### 2.3. Evaluation of the Binding Performances of IIPs

Important parameters that influence the sorption performance of IIPs are the chemical nature of ligand groups, the cross-linking density, but also the characteristics of wastewaters, such as the pH and the coexistence of cross-contaminants [36,37,38]. The main challenge when IIPs synthesis is involved is to reach selectivity and enhance the sorption capacity, as well as to obtain an imprinting effect, which is the main proof that the imprinting process was successful. To assess the imprinting effect, a reference polymer must be also prepared and its features should be compared with those of the IIP. The reference material is called non-imprinted polymer (NIP) [30,31,32,33,34,35,36,37,38]. Both the IIP and the corresponding NIP should be studied in the presence of metal ion solutions: either the template ion or a mixture of the template ion and interfering species. This can be performed in batch or flow mode conditions. From the determination of the ion residual concentration in solution after contact with the IIP or NIP, the removal efficiency (*RE*, %) (Equation (1)) and the adsorption/binding capacity at equilibrium (*q_e_*, mg/g) (Equation (2)) are calculated [30,31,32,33,34,35,36,37,38].

(1) $$RE=\frac{C_{0}-C_{e}}{C_{0}}\times 100$$

(2) $$q_{e}=\frac{\left(C_{0}-C_{e}\right)V}{m}$$

where *C*_0_ is the initial concentration of metal ions (mg/L), *C_e_* is the concentration of metal ions in the aqueous solution at equilibrium (mg/L), *V* is the volume of the aqueous solution (L), and *m* is the mass of IIPs used for extraction (g).

The binding capacity of IIPs materials toward the template ion was also evaluated by applying different mathematical models to the experimental equilibrium and kinetic sorption data (Table 1). Thermodynamic parameters presented in Table 1 have also been calculated by conducting the binding experiments at various temperatures.

The ability of the template ion to be recognized by the IIP in the presence of some interfering species, typically other cations, is evaluated by conducting competitive experiments. These can be binary mixtures [37,42] or multicomponent ones [43]. The selectivity coefficient (*k*) Equation (13) and the relative selectivity coefficient (*k’*) Equation (14) have been defined for IIPs by Dai et al. [44], according to the definition given by Kuchen et al., for metal-ion exchange resins [45].

(13) $$k=\frac{K_{D}\left(template\;ion\right)}{K_{D\;}\left(interfering\;ion\right)}$$

(14) $$k^{\prime }=\frac{k_{IIP}}{k_{NIP}}$$

where *K_D_* represents the distribution coefficient and is calculated by Equation (15); *k_IIP_* and *k_NIP_* are the selectivity coefficients of IIP and NIP, respectively.

(15) $$K_{D}=\frac{q_{e}}{C_{e}}$$

where *q_e_* is the sorption capacity at equilibrium, mg/g; *C_e_* represents the equilibrium concentration, mg/L.

The higher the values of *k* and *k’*, the better the imprinting effect.

## 3. Ion-Imprinted Polymeric Materials for Selective Extraction of Transition Metal Ions

### 3.1. Copper-Imprinted Polymers

Cu(II) is an essential micronutrient that plays an important role in chlorophyll formation and photosynthesis in plants, being also an enzymatic co-factor in animals [46,47]. Although having a high tolerable limit in drinking water (i.e., 2 mg/L), exceeding it can be detrimental to the health of plants and animals. The main sources of Cu(II) water pollution are anthropogenic activities: mining, metal processing, domestic wastes, and different production industries [46]. In this regard, different Cu(II) IIPs have been developed over the years as promising strategies to efficiently and selectively capture Cu(II) from wastewater. Synthetic Cu(II) IIPs are usually prepared according to the succession of steps depicted in Figure 2: (i) the complexation of Cu(II) ions with different monomers, such as AAm [48,49,50], AA [51], MAA [52,53,54,55], methyl methacrylate (MMA) [56], hydroxyethylmethacrylate (HEMA) [57], 4-VP [52,55], picolinic acid (PA) [56], MAH [57], itaconic acid (IA) [58], etc.); (ii) polymerization of the formed complex in the presence of a cross-linker; and (iii) removal of the template Cu(II) ions after (co)-polymerization [52]. To enhance their chemical and mechanical stability and to improve their selectivity toward Cu(II) ions, many composite IIPs have also been engineered. For instance, Cu(II)-imprinted composite hydrogels have been prepared by Wang et al., through the free-radical polymerization of AAm in the presence of Cu(II) ions as the template and amino-functionalized SiO_2_ as inorganic support [48]. The composite hydrogels obtained after leaching of the imprinted ions were capable of selectively rebinding Cu(II) from binary mixtures with Pb(II), Cd(II), or Ni(II) ions. Moreover, they reached a maximum sorption capacity of 538 mg Cu(II)/g, at pH 5 and a contact time of 20 min. The adsorption kinetics followed the PSO model, thus supporting chemisorption as the rate-controlling step. Cu(II)-imprinted magnetic and non-magnetic membranes were developed by Jalilzadeh et al. by the free-radical polymerization of HEMA and of a pre-formed Cu(II)-MAH complex under cryogelation conditions [57]. Following the elution of template ions from both types of cryogels, their ability to rebind Cu(II) ions was thoroughly investigated as a function of initial pH and Cu(II) concentration, temperature, contact time, selectivity, and reusability. The cryogels sorption performance toward Cu(II) ions increased with the amount of Cu(II)-MAH complexes and Fe_3_O_4_ nanoparticles used during their synthesis. Maximum sorption capacities of 77.2 mg Cu(II)/g and 179 mg Cu(II)/g have been attained for the P(HEMA-*co*-MAH) and Fe_3_O_4_/P(HEMA-*co*-MAH) cryogels, respectively, at pH 5.5 and 2 h contact time. Furthermore, both Fe_3_O_4_/P(HEMA-*co*-MAH) and P(HEMA-*co*-MAH) were highly selective for Cu(II) ions recovery from multi-HMIs solutions containing Cd(II), Pb(II), and Zn(II) ions, higher selectivity coefficients being obtained in the case of magnetic cryogels (Table 2). The higher Cu(II) sorption capacity of magnetic cryogels was correlated with the presence of Fe_3_O_4_ nanoparticles, which increased the cryogels’ porosity and surface area, and also led to a nearly homogeneous distribution of imprinted template cavities within their structure [57].

**Figure 5:**
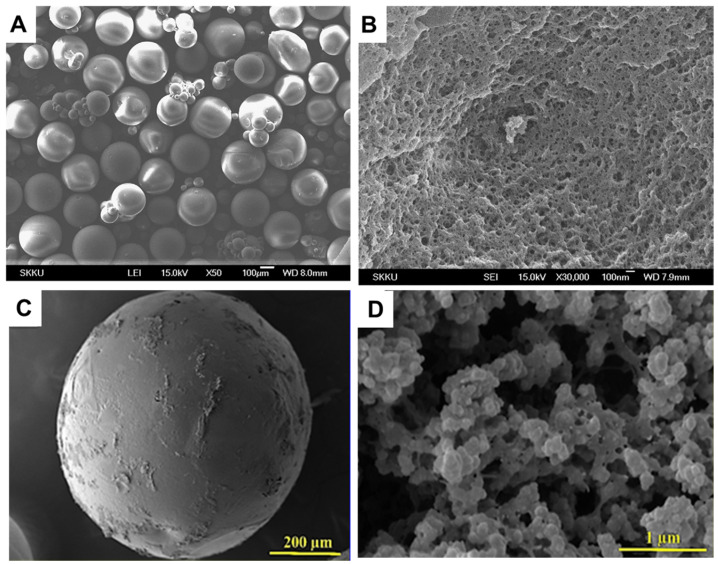
SEM micrographs of Cu(II)-imprinted P(MAA-*co*-4-VP) microparticles (**A**); internal morphology of Cu(II)-imprinted P(MAA-*co*-4-VP) microparticles (**B**) (reproduced with permission from Ref. [52]. Copyright 2010 Elsevier); SEM micrographs of Cu(II)-imprinted P(MMA-*co*-PA) beads (**C**); internal morphology of Cu(II)-imprinted P(MMA-*co*-PA) beads (**D**) (reproduced with permission from Ref. [56]. Copyright 2020 Elsevier).

IIP beads with large surface areas are often preferred for wastewater purification applications because they facilitate the timely interaction between pollutants and ligand groups, and could be used in packed bed columns. Thus, Cu(II)-IIP beads with different sizes, porosity, and morphology were also prepared by free-radical polymerization methods using different combinations of monomers and cross-linking ratios [52,56,58]. For instance, Cu(II)-imprinted porous microparticles (Figure 5A,B) were prepared by suspension polymerization using toluene as porogenic solvent, MAA and 4-VP as functional monomers, and EGDMA as cross-linker [52]. Toluene was also used to obtain porous Cu(II)-imprinted beads with ≈450 µm size (Figure 5C,D) and surface area of 95 m^2^/g by the suspension polymerization of MMA (monomer) and EGDMA (cross-linker) in the presence of Cu(II)-PA (functional ligand) complex [56]. Due to the large surface area, the imprinted P(MMA-*co*-PA) beads exhibited remarkably fast sorption kinetics, the sorption equilibrium being reached in 1 h, at pH 5. It was also highly selective for Cu(II) separation from its mixtures with Ni(II), Co(II), and Zn(II) ions. Despite the similar radii of Cu(II) (73 pm), Ni(II) (69 pm), Co(II) (65 pm), and Zn(II) (74 pm), the obtained *k* values were 26.8, 34.0, and 57.7, respectively, thus demonstrating the important role of imprinting process on the selectivity of the IIP beads. Furthermore, their production could be easily scaled-up to be used as packed bed material for the selective recovery of Cu(II) ions from wastewaters containing complex matrices of pollutants.

Lately, much attention has been paid toward developing polysaccharide-based Cu(II)-IIP materials [23,42,43,59,60,61,62,63,64,65,66], because of their widespread bioavailability and abundance of functional groups that are capable to interact with Cu(II) ions. Furthermore, to improve their sorption performance, mechanical properties, and/or recovery from wastewaters, different inorganic particles, including Fe_3_O_4_ [59,63], zeolite [23,42,43], montmorillonite (MMT) [65], ATT [60], or GO [62], have been employed to fabricate polysaccharide-based Cu(II)-IIP composite materials. Table 2 presents the sorption performances of these materials in comparison with those based on synthetic 3D matrices. For example, magnetic CS microspheres with maximum sorption capacities of 109.89 mg Cu(II)/g have been prepared by Cai et al. [59] by the co-precipitation in alkaline media. Kinetic studies showed that the sorption of Cu(II) ions has been described better by the PSO model, indicating chemisorption as the main adsorption mechanism. Both Fe_3_O_4_-CS composites exhibited good selectivity for Cu(II) adsorption from multi-HMIs solutions. Selectivity coefficients of 38.866, 7.103, 31.679, and 2.297 for Cu(II) removal from its binary solutions with Zn(II), Ni(II), Cd(II), or Cr(VI), respectively, have been determined for the imprinted Fe_3_O_4_-CS microspheres [59].

**Figure 6:**
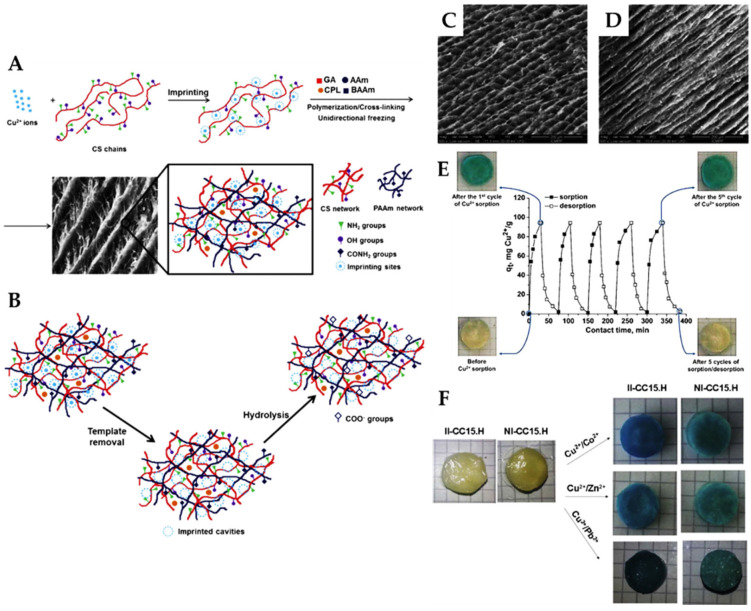
Preparation of Cu(II)-imprinted PAAm/CS/zeolite composite cryogels (**A**) and the removal of template Cu(II) ions and partial hydrolysis of amide groups in PAAm (**B**) (reproduced with permission from Ref. [42]. Copyright 2018 Elsevier). SEM micrographs of Cu(II)-imprinted PAAm/CS/zeolite composite cryogels after the elution of Cu(II) ions recorded perpendicular (**C**) and parallel (**D**) to the direction of freezing (reproduced with permission from Ref. [42]. Copyright 2018 Elsevier). The kinetics performance of Cu(II)-imprinted PAAm/CS/zeolite composite cryogels in successive sorption and desorption of Cu(II) ions (**E**) (reproduced with permission of Romanian Academy Publishing House, the owner of the publishing rights, Ref. [23]). Optical images of Cu(II)-imprinted (denoted as II-CC15.H) and non-imprinted (denoted as NI-CC15.H) PAAm/CS/zeolite cryogels before and after HMIs sorption in multicomponent solutions (**F**) (reproduced with permission from Ref. [42]. Copyright 2018 Elsevier).

Dinu et al., developed Cu(II)-imprinted PAAm/CS/zeolite cryogel composites with pre-organized recognition sites and tailored porous structure by combining the ion-imprinting and the ice-templating methodologies (Figure 6) [23,42].

Cu(II)-imprinted CS/zeolite composite cryogels have also been prepared by the same approach [43]. The Cu(II) ions were first chelated with the –NH_2_ and/or –OH groups of CS to obtain a Cu(II)/CS complex. Then, GA, as cross-linker, and zeolite microparticles with or without AAm and MBAAm as monomers, as well as the initiators, were added to the reaction mixture (Figure 6A). The geometry of Cu(II)-imprinted cavities was preserved through GA cross-linking, even after its leaching (with EDTA) from the cryogels. Furthermore, to improve the overall HMI chelation performance of the cryogel composites, the partial hydrolysis of the amide groups in PAAm was also performed to generate carboxylate groups (Figure 6B) [23,42].

Anisotropic Cu(II)-imprinted composite cryogels with tube-like interconnected pores were obtained by combining IIT with the unidirectional ice-templating method (Figure 6C,D) [23,42]. This allowed a fast diffusion of HMIs inside the cryogels structure. A maximum sorption capacity of 260 mg Cu(II)/g composite was reported for the Cu(II)-imprinted PAAm/CS/zeolite composite cryogels, at pH 4.5 and 1 g/L sorbent dose; the equilibrium of sorption being attained in only 20 min (Table 2) [23,42]. In comparison, the Cu(II)-imprinted CS/zeolite composite cryogels were capable of retaining only 55.08 mg Cu(II)/g composite, at pH 4.5, and a contact time of 150 min, but in multi-HMIs solutions [43]. The higher sorption capacity of Cu(II) ions by the former composites could be attributed to the presence of hydrolyzed amide groups from PAAm into their structure. The Cu(II)-imprinted PAAm/CS/zeolite composite cryogels preserved their ability to retain Cu(II) ions during five reusing cycles without shape changes or decline of sorption capacity (Figure 6E) [42]. Furthermore, they were highly selective for Cu(II) ion sorption from its mixtures with interfering HMIs such as Co(II), Zn(II), and Pb(II) ions, as illustrated by the more intense blue color of imprinted cryogels compared to the non-imprinted ones (Figure 6F). Selectivity coefficients of 7.28, 24.42, 47.02, and 34.85 were reported for Cu(II) separation from its mixtures with Co(II), Ni(II), Zn(II), and Pb(II) ions, respectively (Table 2). Thus, the competing Co(II) and Ni(II) ions did not match the well-defined Cu(II)-imprinted cavities because of their smaller ionic radius (65 pm for Co(II) ions and 69 pm for Ni(II) ions) [42]. Although Zn(II) ions have a similar ionic radius (74 pm) compared with that of Cu(II) ions (73 pm), their significantly lower electronegativity (1.65) led to the highest *k* values for Cu(II) sorption from Cu(II)/Zn(II) mixtures. This highlights the pivotal role of ligand groups’ pre-organization around the template ion during the imprinted preparation of cryo-composites [42].

**Figure 7:**
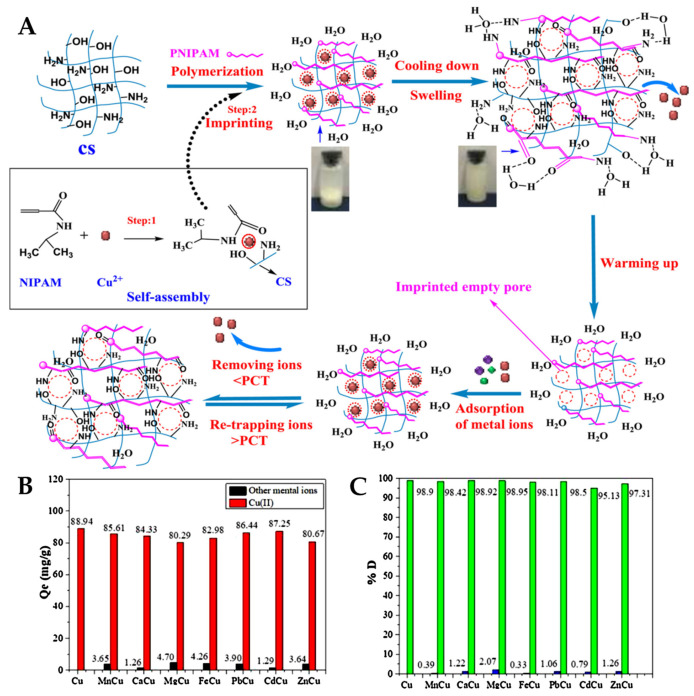
Representation with the preparation, and HMIs adsorption and desorption by thermosensitive Cu(II)-imprinted PNIPAAm-CS composites (**A**). Effect of interfering HMIs on the equilibrium adsorption capacity (*q_e_*, mg/g) (**B**) and desorption percentage (D, %) (**C**) of Cu(II)-imprinted PNIPAAm-CS composites. (Adapted with permission from Ref. [64]. Copyright 2020 Elsevier).

A sustainable IIP sorbent with outstanding selectivity for Cu(II) in multi-HMIs solutions was prepared by the grafting polymerization of *N*-isopropylacrylamide (NIPAAm) onto CS in the presence of template Cu(II) ions using a gradient heating process (Figure 7A) [64].

The sorbent exhibited a phase change temperature (PCT) at 35.31 °C in the heating cycle, and at 26.27 °C in the cooling cycle. After synthesis, the template Cu(II) ions were removed from the sorbent by decreasing the temperature below the PCT, followed by washing with cold water. The PNIPAAm chains elongated at temperatures below PCT, thus disrupting the interactions between Cu(II) ions and the functional groups of the sorbent. By subsequently increasing the temperature above PCT, the sorbent shrunk again to regain the sorption properties toward Cu(II) ions. The sorbent attained a maximum sorption capacity of 105.49 mg Cu(II)/g at 318 K, pH 6, and 0.5 g/L sorbent dose. The reversible phase change allowed a 96.05% sorption capacity recovery for Cu(II) ions after five successive sorption–desorption cycles. Furthermore, the imprinting process conferred the sorbent with selectivity toward not only the sorption of Cu(II) ions from a multi-HMIs solution with Mn(II), Ca(II), Mg(II), Fe(III), Pb(II), Cd(II), and Zn(II) ions (Figure 7B), but also for its desorption (Figure 7C) [64].

In another work, Qin et al., developed Cu(II)-imprinted MMT/CS gel beads as promising sorbents for the selective removal of Cu(II) from multi-HMIs solutions [65]. The Cu(II) sorption capacity of these composite gel beads increased with the increase in MMT content in their structure, reaching 84 mg/g, 92 mg/g, and 118 mg/g at 0%, 20%, and 45% MMT. The enhancement of Cu(II) sorption performance with the increase in MMT content was attributed to the capacity of MMT to interact with HMIs by cation exchange. Moreover, the imprinting process conferred excellent selectivity to the MMT/CS composite gel beads for the sorption of Cu(II) from its mixture with Ni(II), Cd(II), Zn(II), and Co(II) ions.

A thermoresponsive Cu(II)-imprinted bacterial cellulose (BC) derivative (ITB) was prepared from BC and NIPAAm as the thermoresponsive functional monomer [67]. The produced Cu(II)-ITB had high selectivity and adsorption capacity (140.85 mg/g) with two phase transition temperatures at 38.57 °C and 30.81 °C; thus, the Cu(II)-ITB could be recycled by adjusting its temperature to these threshold values.

Cu(II)-selective ion-imprinting membranes using triethylenetetramine (TETA) as ligand were prepared by combining IIT with membrane technology and were successfully used for selective separation of Cu(II) ions from the metal finishing and electroplating processes [68]. Combining surface IIT with self-assembly techniques, Wang et al. [69] developed self-assembled surface Cu(II)-imprinted membranes consisting of PAA and CS.

### 3.2. Cadmium-Imprinted Polymers

Cd(II) is one of the most hazardous HMIs, being highly toxic and carcinogenic. The main sources of Cd(II) contamination are mining, metal plating, electroplating, and chemical industries [70]. Different materials with improved performance have been prepared in recent years by the ion-imprinting technique for Cd(II) detection [71,72] and adsorption [73,74,75,76,77,78,79,80] from wastewaters (Table 3).

Bio-based Cd(II)-imprinted CS beads with a maximum sorption capacity of 123.65 mg Cd(II)/g (at pH 7, 0.65 mg/L sorbent dose and 48 h contact time) were recently prepared by Babakhani et al., by ionotropic cross-linking with TPP [73]. Compared with the non-imprinted CS beads, a 21 ± 5% sorption capacity enhancement was determined for the Cd(II)-imprinted ones because of the specific recognition sites for Cd(II). The kinetic sorption data were best fitted by the PSO model, supporting chemisorption as the dominant adsorption mechanism, through chelation with the free amino groups of CS and phosphate groups of TPP. By combining the ion-imprinting technique with ECH cross-linking, Rahangdale et al., prepared Cd(II)-imprinted AAm-*g*-CS gels that were used for the selective recovery of Cd(II) ions from acidic leachate of Ni-Cd battery waste [74]. The imprinted gels exhibited a maximum sorption capacity of 167 mg Cd(II)/g at pH 6, 2 h contact time, and 2 g/L sorbent dose. The selectivity coefficients toward Cd(II) recovery from multi-HMIs solutions containing Ag(I), Cu(II), Ni(II), and Zn(II) were 4.56, 4.13, 4.11, and 4.20, respectively. Furthermore, the Cd(II)-imprinted AAm-*g*-CS gels were capable to recover 84.3% of Cd(II) ions from a complex leachate of Ni-Cd battery waste [74]. Cd(II)-imprinted composite CMCS-SiO_2_ sorbents with a maximum sorption capacity of 20.7 mg Cd(II)/g and fast sorption kinetics (30 min) in batch experiments were prepared by Lü et al. [76]. When used as a solid phase matrix in column experiments, the CMCS-SiO_2_ composites were capable of effectively removing Cd(II) even in the presence of interfering Ca(II), Mg(II), Zn(II), Co(II), Cu(II), and Pb(II) ions, with an enrichment factor of 257.

Murat et al., prepared Cd(II)-imprinted MA-*co*-AN/DVB microspheres via the precipitation polymerization technique for selective Cd(II) removal from multi-HMIs wastewater in column setups [79]. These microspheres exhibited a maximum sorption capacity of 20.46 mg Cd(II)/g, at pH 6 and 0.5–2 mL/min flow rate. The sorbed Cd(II) ions were successfully eluted with 1 mol/L HNO_3_ aqueous solution to regenerate the column. Selectivity coefficients in dynamic competitive sorption experiments for the separation of Cd(II) ions from multi-HMIs solutions containing Cu(II), Mn(II), Ni(II), and Pb(II) ions were 15.2, 4.10, 9.20, and 3.01, respectively. The strong affinity of imprinted microspheres toward Cd(II) ions was attributed to the dimensional and geometrical matching between the template cavities and the ionic radius of Cd(II) (95 pm), compared to the ionic radii of Cu(II) (73 pm), Mn(II) (83 pm), Ni(II) (69 pm), and Pb(II) (119 pm).

Fast sorption kinetics and high selectivity coefficients in multi-HMIs wastewaters were reported by Li et al. [77] and Wang et al. [78] for Cd(II)-imprinted ATU/N-propylmaleamic acid-functionalized SiO_2_ composites and HMAM/DVE3 IPN hydrogels, respectively. For example, the Cd(II)-imprinted ATU/N-propylmaleamic acid-functionalized SiO_2_ composites (prepared using acetonitrile as porogenic solvent) reached the sorption equilibrium in 8 min and a maximum sorption capacity of 38.30 mg Cd(II)/g at pH 5 and 1 g/L sorbent dose [77]. This composite showed excellent selectivity toward Cd(II) recovery in binary solutions containing Ni(II), Cu(II), Co(II), Pb(II), or Zn(II) ions, the selectivity coefficients being 18.64, 2.55, 6.27, 20.82, and 15.41, respectively. This behavior was assigned to a combination of factors, including the size and geometry of imprinted cavities, as well as the inherent affinity of C=S function groups (soft base) toward Cd(II) ions (soft acid). In another work, the sorption equilibrium for Cd(II) ions was reached in 16 min using Cd(II)-imprinted HMAM/DVE3 IPN hydrogels [78]. In addition, these IPN hydrogels exhibited a maximum sorption capacity of 179.86 mg Cd(II)/g, at pH 6. The adsorption mechanism established by FTIR and XPS indicated that the sorption of Cd(II) ions proceeded through chelation with the –NH, –OH, C=O, and C–O groups of the hydrogel. Moreover, the Cd(II) imprinting resulted in a high increase in the IPN hydrogel’s selectivity toward Cd(II) in the presence of competitive ions with the charge and similar ionic radii, such as Cu(II), Ni(II), and Pb(II), and the selectivity coefficients being 8.33, 8.79, and 9.18, respectively.

To investigate the influence of functional groups on the selectivity performance, Adauto et al., synthesized new Cd(II)-IIPs using 1-VI as a functional monomer, and MP or AMP as functional organosilane, in the presence of TRIM as a cross-linker [80]. Maximum sorption capacities of 16.99 mg Cd(II)/g and 10.40 mg Cd(II)/g were determined for the imprinted 1-VI/MP/TRIM and 1-VI/AMP/TRIM IIPs, at pH 7.2 and 1 g/L sorbent dose. Both Cd(II)-imprinted polymers displayed *k’* higher than unity, in binary solutions of Cd(II) with Pb(II), Zn(II), Hg(II), Cu(II), Ni(II), Ca(II), Mg(II), or Na(I) ions compared to the non-imprinted materials, because of the specific recognition sites for the Cd(II) ions. The selectivity coefficients showed comparable trends for the Cd(II)-IIPs prepared with MP or AMP. However, the obtained values were considerably higher for the materials prepared with MP, indicating that the affinity of -SH groups toward Cd(II) ions is higher than that of the –NH_2_ groups [80].

### 3.3. Zinc-Imprinted Polymers

Zinc is one of the most important essential trace elements in human nutrition, and the second most abundant trace metal in the body [81,82]. Zinc deficiency is at the origin of several disorders including growth retardation, diarrhea, eye and skin lesions, immunity depression, and malfunction of wound healing. However, excess consumption of Zn(II) ions causes a disparity in several chronic and cellular processes leading to adverse effects including alteration in the immune response, weight loss, neurodegenerative diseases such as the formation of amyloid plaques in Alzheimer’s disease, Menkes disease, Wilson’s disease, Parkinson’s disease, and prostate cancer, hypocalcemia, and bone resorption [81,82,83,84]. The metal coating industry is among the main sources of Zn(II) in wastewaters. Therefore, an efficient method for the determination of trace amounts of Zn(II) in complex matrices such as river water, food, vegetables, and whole grain cereals is imperative. This task can be achieved by selective separation, concentration, and accurate determination of Zn(II) ions. SPE is one of the most used sample pretreatments. Among the different sorbents used for SPE, IIPs have attracted much attention as selective sorbents for the preconcentration of metal ions. However, reports on the synthesis of the IIP selective for Zn(II) are limited (see Table S1) [83,84,85,86,87,88,89,90,91,92,93]. For example, 8-HQ has been used to prepare Zn(II)IP for separation and preconcentration of Zn(II) from different matrices prior to its determination, by mixing the binary complex of Zn(II) with 8-HQ, using styrene (ST) as the monomer and EGDMA as the cross-linker followed by thermal polymerization [83], or MAA as the monomer, EGDMA as the cross-linker and ethanol/acetonitrile mixture as porogen, followed by precipitation polymerization [86]. The selectivity of the IIPs prepared with ST as the monomer was investigated in the presence of various cations and anions, either present in a real sample or able to form a complex with 8-HQ, i.e., Ni(II), Co(II), Ag(I), Cd(II), Al(III), Fe(III), Cu(II), Pd(II), Pb(II), Ca(II), Cl^−^, Br^−^, I^−^, and CO_3_^2−^, at an initial mole ratio of 1000 (ion/zinc). The results evidenced that the recovery of Zn(II) was quantitative (recovery > 95.3%) in the presence of an excessive amount of possible interference ions indicating that, under the optimum conditions, the synthesized IIP behaves as a selective sorbent for Zn(II). Zn(II)-IP extractors have been effective in the preconcentration of Zn(II) from various waters (tap, well, rain, river, and sea waters) and cereal samples, with a level of recovery > 96%.

**Figure 8:**
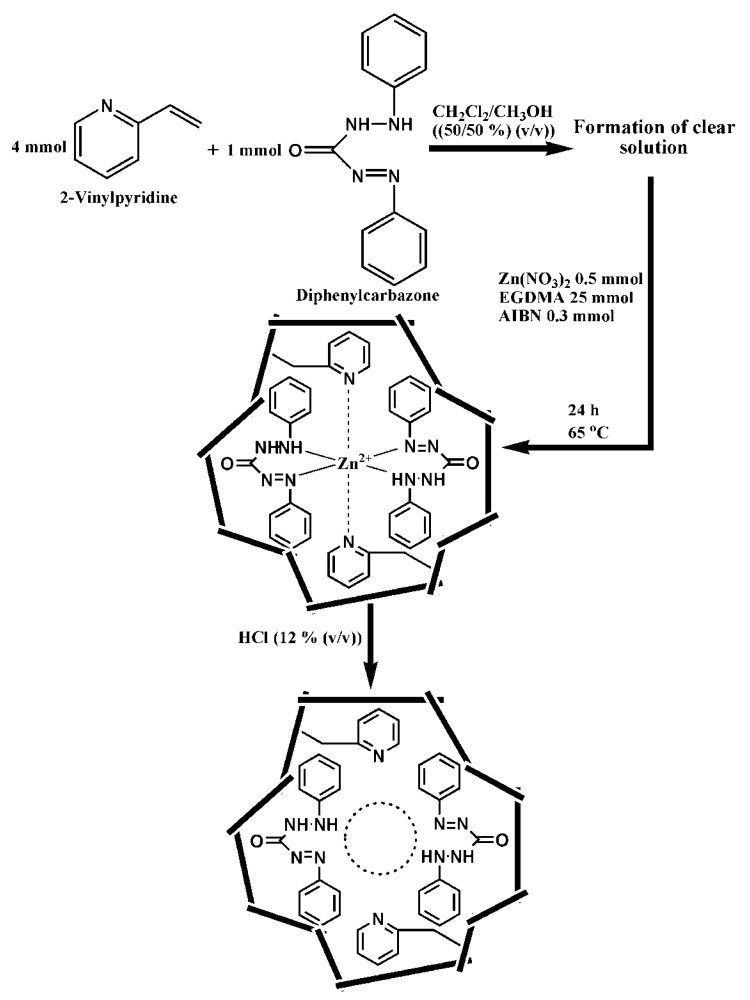
Schematic illustration of imprinting process for the preparation of Zn(II)-IP by bulk polymerization. (Adapted with permission from. Ref. [89]. Copyright 2014 Elsevier).

A Zn(II)-imprinted polymer was synthesized via precipitation polymerization of the pre-complex formed between Zn(II) with 1-VI (1:4) in the presence of HEMA and EGDMA [84]. The aim of HEMA addition was to increase the surface hydrophilicity of the Zn(II)-IP particles. Competitive adsorption of Zn(II) in the presence of Cu(II), Co(II), and Ni(II) was investigated from a binary solution of Zn(II) and the interfering ions, in batch mode. The selected competitor ions have the same charge and close ionic radii (Zn(II) = 74 pm, Cu(II) = 73 pm, Ni(II) = 69 pm, and Co(II) = 65 pm). The *K_d_*, *k,* and *k’* (the ratio between the selectivity factor of imprinted to that of non-imprinted polymer) were evaluated [84]. A high selectivity toward Zn(II) was exhibited by the Zn(II)-IP compared with NIP, the values of *k’* ranged from 5.44 to 46.2, depending on the interfering ion. Another Zn(II)-IP was synthesized by the copolymerization of 2-vinylpyridine (2-VP) as the monomer and EGDMA as the cross-linker, Zn(NO_3_)_2_ as a template salt, diphenylcarbazone as the ligand, and the methanol/dichloromethane mixture as a solvent (Figure 8) [89].

The designed sorbent was tested for the extraction and determination of Zn(II) ions as trace levels in river water, vegetables, red meat, and milk samples. Under the optimized conditions, the detection limit of the proposed method was found to be 0.15 mg/L. The degree of template ion recovery in the presence of some alkaline, alkaline earth, and transition metal ions was determined, and found a level of recovery >96% for Zn(II) and (5–8)% for interfering ions. From the tolerance data, it was found that the foreign ions did not significantly influence the preconcentration of Zn(II) ions at pH 6.6. The authors also reported that the Zn(II)-IP particles can be repeatedly used with no significant decrease in their performances [89]. Trace levels of Zn(II) at concentrations of ng/mL, in high-volume samples, could easily be separated by the Zn(II)-IP.

**Figure 9:**
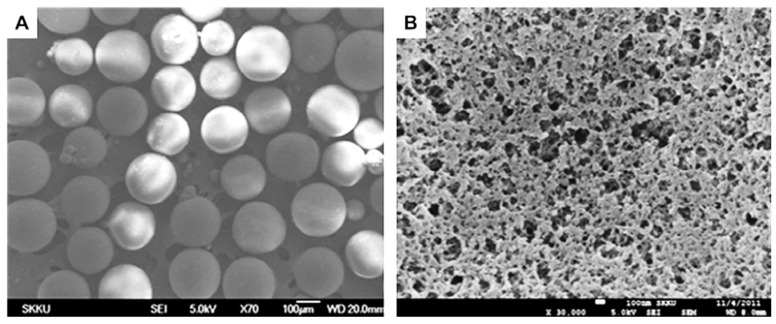
SEM micrographs of (**A**) exterior and (**B**) interior of Zn(II)-IP particles composed of 2,2′-bipyridyl and 4-VP monomers cross-linked with EGDMA (reproduced with permission from Ref. [90]. Copyright 2013 Elsevier).

Porous polymer beads were prepared from a self-assembled complex composed of 2,2′-bipyridyl and 4-VP monomers and Zn(II) as the template ion, EGDMA as the cross-linker, and toluene as porogen via suspension polymerization (see Table S2, Figure 9A,B) [90].

As the particles possess many pores (Figure 9B), a very high adsorption capacity was measured for Zn(II) ions at 210.61 µmol/g, while those for other ions were much lower, i.e.: 37.92 µmol/g, 33.02 µmol/g, and 9.70 µmol/g for Cu(II), Ni(II), and Pb(II), respectively. The selective separation characteristics of the Zn(II)-IP beads were analyzed by calculating the distribution ratio, *D_i_*, *k*, and *k’* for Cu(II), Ni(II), and Pb(II) as interfering ions. The experimental pH was 6.2 and the concentration of each metal ion was 0.15 mmol/L. The adsorption capacity for Zn(II) was much higher than that of any competitor ions, and its value became even higher with the increase in the cross-linker/monomer (C/M) ratio. This is probably due to the increase in the surface area per unit mass adsorbent polymer particle in association with the reduced particle size (diameter) at an increased C/M ratio. On the other hand, the NIP beads (at a C/M ratio of 8) did not exhibit high adsorption capacity and selective separation of Zn(II). As the selectivity coefficients for Zn(II) to Cu(II) and to Ni(II) were 8.52 and 52, respectively, it can be stated that the Zn(II)-IP synthesized in this study possessed a high selective binding of Zn(II) ions. The high values of *k’* support a strong imprinting effect of the polymeric adsorbent synthesized by this strategy. The Zn(II)-IIP particles lost only about 10% of their adsorption capacity after 10 repeated uses.

In another work, 3,5,7,2′,4′-pentahydroxyflavone (morin), MAA, and EGDMA were used as the Zn(II) ion-selective complexing reagent, functional monomer, and cross-linker, respectively, for the preparation of Zn(II)-IP by the bulk polymerization process [85]. Zn(II)-IP nanoparticles were tested as a solid phase extractor for the uptake of Zn(II) from aqueous solutions. The selectivity of the Zn(II)-IP sorbent toward Zn(II) ions was evaluated in binary systems with cations having close atomic radii, and identical physicochemical properties, such as Cu(II), Ni(II), Cd(II), Pb(II), and Co(II) in comparison of IIP with NIP sorbents, under optimum conditions. The *k* and *k’* values for Zn(II) ions against interfering ions were calculated, and it was found that the competitive adsorption capacity of Zn-IIP nanoparticles for Zn(II) ions was much larger than for that of NIP. Thus, the *k’* values were 30.7, 23.9, 26.4, 42.6, and 26.8 when the competitor ions were Cu(II), Co(II), Ni(II), Pb(II), and Cd(II), respectively. It was obvious that in the case of the IIP prepared with Zn(II)/morin complex as the template, the 3D cavities that remained after the leaching of Zn(II) ions, which have a specific shape and size of the template, were a more selective for target ions than the NIP. Based on these results, the authors concluded that the prepared IIP could be applied as a selective sorbent for the separation of Zn(II) ions in the presence of other metal cations in complex matrices [85]. The sorbent affinity for Zn(II) was not diminished by repeated uses during 5 months.

Salicylic acrylate as a chelating ligand has been complexed with Zn(II) and then thermally polymerized with EGDMA [87]. The chelating property of the salicylate structure is due to the existence of an OH group in the ortho position relative to the carboxyl or carbonyl group that can react with both hard and intermediate cations. Competitive adsorption of Zn(II) in the presence of Cd(II), Ni(II), and Co(II) was investigated at the optimum pH (6.0). The prepared Zn(II)-IPs showed an extraction efficiency of Zn(II) much higher for Zn(II) than for the competitor cations. Thus, the extraction efficiency of Zn(II) at pH 6.0 was 94.9%, while for Co(II), Ni(II), and Cd(II), it was 35.5%, 10.5%, and 19.9%, respectively [87].

It was found that the IIPs prepared by bulk and precipitation polymerizations presented a heterogeneous distribution of the binding sites, which led to poor accessibility of the target ion to the functional groups in the cavities, because many of the functional groups and template ions are embedded inside the polymer network, and to a slow mass transfer [91]. Surface imprinting has been tested as an opportunity to improve the distribution and accessibility of the binding sites on the surfaces of IIPs [91,92,93]. Graphene oxide (GO) is one of the most attractive supports for the preparation of IIPs due to its large surface area and presence of numerous functional groups (hydroxyl, epoxide, and carboxylic). A novel Zn(II)-IP sorbent has been reported by Kazemi et al., using GO as supporting material [91]. The magnetic nanocomposite of CS/GO cross-linked with GA modified with AA was prepared first. Zn(II) was complexed with zincon as the ligand (see Table S2), and then mixed with the magnetic nanocomposite CS/GO, and finally with ST as the monomer and EGDMA as the cross-linker, the magnetic nanocomposite Zn(II) IIP-GO/CS being obtained by coprecipitation polymerization [91]. This nanocomposite sorbent manifested high selectivity, and fast mass transfer for Zn(II) ions associated with the large surface area of GO and with improved accessibility of the imprinting sites. The selectivity of the synthesized IIP-GO/CS toward Zn(II) ions was investigated by considering the competitive extraction of Zn(II) in the presence of other ions including Cu(II), Co(II), Ni(II), and Cd(II) able to complex with zincon. It was found that the competitor ions did not interfere in the extraction of Zn(II) ions, the level of recovery of Zn(II) being >96%. Moreover, the magnetic IIP-GO/CS nanoparticles could be reused without significant loss of the selective sorption performance up to nine sorption–desorption cycles. The feasibility of the method was demonstrated by the determination of Zn(II) ions in real samples including well water, drinking water, black tea, rice, and milk. The accuracy of the procedure was assessed through the recovery experiments from samples spiked with known amounts of Zn(II). The results demonstrated that the recoveries of the spiked ions ranged from 96.0 to 106.0%.

The surface imprinting method was used by Yu et al., to prepare a novel IIP on the surface of ATT [93]. For this purpose, Zn(II) ions were coordinated to the functional groups of CS. The addition of ATT and 3-glycidoxypropyltrimethoxysilane (A-187) to the Zn(II)/CS complex generated the composite by grafting silanol groups of A-187 onto the surface of ATT through the acid-catalyzed self-hydrolysis, while the epoxy groups of A-187 were connected with the Zn(II) coordinated to CS. Thus, a Zn(II)-IP was formed on the surface of ATT. The selectivity of the Zn(II)-IIP was investigated by competitive adsorption of Zn(II) from their mixture with Cd(II), Cs(I), Co(II), Ba(II), Sr(II), and Pb(II). The ion-imprinting effect was clearly observed as the Zn(II)-IIP had a higher adsorption efficiency of Zn(II) than of any other metal ions. Moreover, the *k’* values higher than seven indicated that Zn(II) ions can be selectively adsorbed from their binary mixture with Cd(II), Cs(I), Co(II), Ba(II), Sr(II), and Pb(II).

A thermosensitive ion-imprinted hydrogel (IIH) with an IPN structure was prepared by the thermal polymerization of *N,N*-dimethylaminoethyl methacrylate (DMAEMA) and photoinitiated cationic polymerization of bis-(3,4-epoxycyclohexyl)adipate (BECA) with Zn(II) ion as template [88]. The imprinted cavities in the IPN gel displayed a volume phase transition with the increase in temperature up to 50 °C associated with the thermosensitivity of the poly(*N*,*N*-dimethylaminoethyl methacrylate) (PDMAEMA) network. It has been observed that the highest affinity of the IIH for Zn(II) was located at 50 °C because the shape of the imprinting cavities and the distribution of the functional groups fitted better to Zn(II) ions at this temperature. The selectivity of IIH toward Zn(II) ions, in the presence of Cu(II), Ni(II), and Co(II) as interfering ions, was evaluated by the values of *k* and *k’*, taking the NIH as the control sorbent. The following values were reported for the selectivity coefficient in the case of IIH, at 50 °C, 8.27, 8.75, and 9.33, for Cu(II), Ni(II), and Co(II) as interfering ions, respectively, while for NIH, the values were 1.14, 1.22, and 1.35. The values of *k* were much lower at 30 °C, for both sorbents, the order of interfering ions being the same: 2.58, 2.67, and 2.74, in the case of IIH, and 1.17, 1.27, and 1.46 for NIH. The values of *k’* at 50 °C were 7.25, 7.17, and 6.91, and at 30 °C were 2.21, 2.1, and 1.88. Therefore, the IIH possessed excellent selectivity toward template ions at 50 °C compared with at 30 °C due to the thermosensitive adsorption of the template ions. The shape transformations of the PDMAEMA network in the IIH at 50 °C, led to the increase in the selective adsorption ability of the IIH, and consequently, the interfering ions were leached out.

### 3.4. Cobalt-Imprinted Polymers

Cobalt is one of the metal contaminants that it enters the environment mostly through industrial activities, such as metallurgy, mining, paint, and electronics. In large quantities, it may lead to various acute or chronic diseases, including those of the gastrointestinal tract, asthma, or pneumonia. Therefore, the effective treatment and removal of Co(II) ions from wastewater is very important [94].

Two types of IIP prepared by free radical cross-linking polymerization of MAA or 4-VP monomers in the presence of dipicolinic acid (DPA)-Co(II) preformed complex and EGDMA (cross-linker) for the selective extraction of Co(II) ions from aqueous medium were reported by Yusof et al. [95]. The poly(methacrylic acid) (PMAA)/DPA and P(4-VP)/DPA IIPs obtained after the extraction of template ions exhibited Co(II) removal efficiencies of 92% and 84%, respectively, at a sorbent dose of 0.6 g/L. The Co(II) ion coordination mode by DPA, along with the functionality of monomers, led to the production of highly selective IIP materials. Selectivity coefficients of 18.46, 13.77, and 30.11 were determined for Co(II) separation from mixtures containing Ni(II), Fe(III), and Mg(II) ions, respectively, using the PMAA/DPA composites (see Table S3). On the other hand, the selectivity coefficients for the same HMIs in the case of P(4-VP)/DPA composites were 9.39, 38.53, and 10.41, respectively. The sorption capacity remained almost unchanged after seven successive sorption–desorption cycles and the composite sorbent preserved its integrity.

Co(II) ion-imprinted membranes were obtained by free radical polymerization of *N*-(pyrrolidin-2-ylmethyl) methacrylamide (PMMA), using AIBN as initiator and EDGMA as cross-linking agent [94]. The equilibrium of sorption was reached within only 45 min and the kinetic data were well fitted by the PSO model. It was found that the sorption process of Co(II) ions was better described by the Langmuir isotherm, which confirms that the sorption may have occurred through a monolayer process, with the maximum sorption capacity being 428.24 mg/g. The efficacy of the sorbent decreased by only 16.6%, after four sorption–desorption experiments, demonstrating its enhanced stability in repeated cycles.

The performance of Co(II)-imprinted Fe_3_O_4_/SiO_2_/P(1-VI) composites in the selective separation of Co(II) ions from aqueous solutions has been investigated by Kang et al. [96]. The equilibrium sorption data were best described by the Langmuir isotherm model, with the maximum sorption capacity of Co(II) ions being 23.09 mg/g. Furthermore, the sorption kinetics reached equilibrium in 3 h, and followed the PSO kinetic model, indicating chemisorption as the main sorption mechanism. The *k* values for the Co(II) recovery from mixtures with Cu(II), Cd(II), Zn(II), and Pb(II) were 19.99, 50.28, 11.02, and 7.56, respectively (see Table S3). The IIPs were easily regenerated with 0.5 M HNO_3_, and the removal efficiency remained about 81% even after four sorption–desorption cycles.

Core–shell magnetic Co(II)-IP was synthesized by Khoddami et al., by a surface imprinting technique combined with the sol–gel process using 3-(2-aminoethylamino) propyltrimethoxysilane (AAPTS) as the functional ligand, and tetraethyl orthosilicate (TEOS) as the cross-linker [97]. The equilibrium sorption data were best fitted by the Langmuir isotherm model, with the maximum sorption capacity being 35.21 mg Co(II)/g, at pH 8. The *k* values for Co(II) recovery from multi-HMIs solutions with Pb(II), Ni(II), and Cd(II) ions were 41.17, 79.74, and 56.48, respectively. The imprinted core–shell composite sorbent was highly stable and reusable, its sorption capacity decreasing by only 5.8% after seven sorption–desorption cycles.

In another work, Zhao et al., developed a new Co(II)-IP using magnetic Fe_3_O_4_ nanoparticles as the support, and bis(2-methacryloxyethyl) phosphate (B2MP) and glycylglycine (GG) as functional monomers [98]. The sorption kinetics of P(B2MP-*co*-GG)/Fe_3_O_4_ composites for Co(II) ions was relatively fast; the equilibrium of sorption was reached in 20 min at pH 8.0 and 298 K (see Table S3). The sorption kinetics were well fitted by the PSO kinetic model and the sorption at equilibrium was better described by the Langmuir isotherm, with a maximum sorption capacity of 33.4 mg Co(II)/g. The *k* values for extraction of Co(II) ions from its binary mixtures with Fe(II), Cu(II), Mg(II), Zn(II), and Ni(II) were 5.25, 4.05, 6.06, 11.81, and 4.48, respectively. The P(B2MP-*co*-GG)/Fe_3_O_4_ composites displayed good stability and reusability, and the sorption capacity remained nearly at 90% after six sorption–desorption cycles.

Biocomposites based on 8-HQ anchored on γ-Fe_2_O_3_-CS using ECH as the cross-linker were successfully prepared and applied for the selective removal of Co(II) from aqueous solutions [99]. The magnetic biocomposites had a maximum Co(II) sorption capacity of 100 mg Co(II)/g at pH 8, with the sorption equilibrium being established in 10 min. Kinetic and thermodynamic behaviors were also investigated and the results showed that the sorption of Co(II) ions followed the PSO model and was an endothermic process. The *k* values obtained for the separation of Co(II) from the binary systems with Cd(II), Ni(II), and Pb(II) were 11, 42, and 2, respectively. The biosorbent presented good stability after three cycles of sorption–desorption using 0.5 M of HNO_3_ as eluent.

**Figure 10:**
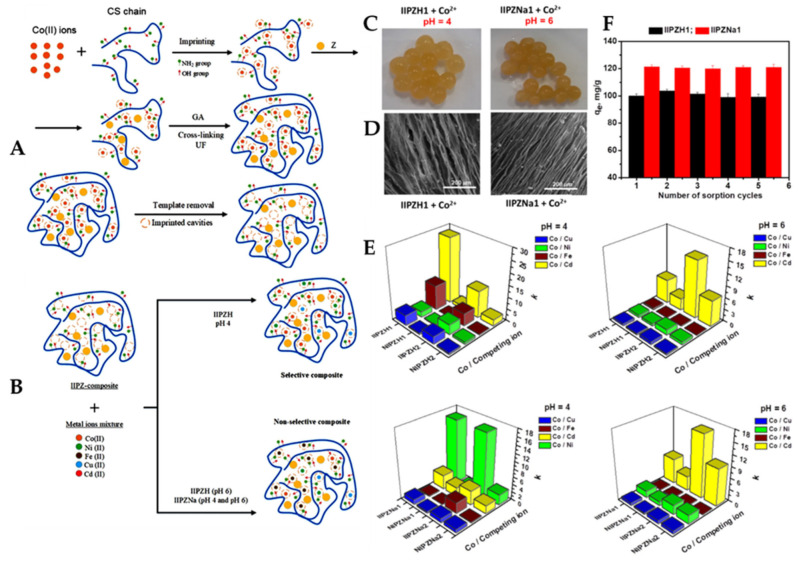
(**A**) The preparation strategy of Co(II)-imprinted CS/zeolite cryo-composites and removal of template ions with ethylenediaminetetraacetic acid disodium salt dihydrate (Na_2_EDTA). (**B**) Selectivity mechanism of Co(II)-imprinted CS/zeolite cryo-composites at pH 4 and pH 6 (ion-imprinted composites = IIPZH and IIPZNa, non-imprinted composites = NIPZ and NIPNa; zeolites (Z1, Z2) converted into the H^+^ or Na^+^ form; Z1 = 60–70 wt% clinoptilolite, Z2 = 82–86 wt% clinoptilolite). (**C**) Optical images of IIPZH1 and IIPZNa1 composites loaded with Co(II) ions at pH 4 and pH 6. (**D**) SEM micrographs of IIPZH1 and IIPZNa1 sorbents loaded with Co(II) ions. (**E**) Selective sorption of Co(II) ions from their five-component mixtures onto the composite cryo-beads. (**F**) The reusability performance of IIPZH1 and IIPZNa1 sorbents after five consecutive sorption–desorption cycles. (Reproduced with permission from Ref. [100]. Copyright 2020 Elsevier).

Novel IIP CS-based composite cryo-beads with remarkable affinity and selectivity for Co(II) ions were prepared by Sáez et al., combining the pH-dependent adsorptive features of activated zeolites with the IIT, using GA as a cross-linker (Figure 10A) [100].

These biosorbents were used in the selective removal of Co(II) ions from multicomponent aqueous mixtures with Cu(II), Ni(II), Fe(II), and Cd(II) ions, at two pH values (pH 4 and 6, respectively) (Figure 10B). The maximum sorption capacity of Co(II) ions was higher when the zeolites incorporated into the IIP CS-based composite cryo-beads were conditioned with NaCl (120.4 mg/g for IIPZNa1 and 126.6 mg/g for IIPZNa2) than when they were treated with HCl (102.3 mg Co(II)/g IIPZH1 and 86.2 mg Co(II)/g IIPZH2). Moreover, these results were sustained by the darker pink color of the Co(II)-loaded cryo-beads conditioned with salt-activated zeolites (IIPZNa1, Figure 10C). The SEM micrographs of the Co(II)-loaded cryo-beads (IIPZH1 and IIPZNa1) show their well-defined lamellar patterns, even after HMIs sorption (Figure 10D). Both ion-imprinted composites (IIPZH and IIPZNa) and non-imprinted composites (NIPZH and NIPZNa) were capable of sorbing Co(II) ions from their mixtures with Cu(II), Ni(II), Fe(II), and Cd(II) ions (Figure 10E). The IIPZH CS-based composite cryo-beads were strongly selective toward Co(II) sorption in mixtures containing Cu(II), Ni(II), Fe(II), and Cd(II) ions, with the selectivity coefficients being in the range 2.3–28.1, at pH 4 (see Table S3). Furthermore, the selectivity coefficients of IIPZNa sorbents were situated in the range 0.05–16.5, at pH 6. The sustainability of IIP CS-based composite cryo-beads was demonstrated by the complete recovery of their sorption capacity for Co(II) ions in five sorption–desorption cycles (Figure 10F).

### 3.5. Nickel-Imprinted Polymers

Nickel is a common toxic heavy metal, which is mainly detected as Ni(II) in wastewaters coming from electronic industries, electroplating, batteries, and mining. In trace amounts, nickel is an essential element for the human body that comes from vegetables and grains. However, ingestion of excessive amounts of Ni(II) leads to abnormal skin color and skin diseases, cancer, and nervous disorders [101]. Consequently, its removal is highly demanded. Thus, several Ni(II)-imprinted materials have been prepared [101,102,103,104,105,106,107,108,109,110,111,112] (Table 4).

A new imprinted biosorbent, as film, for selective Ni(II) sorption was obtained using CS as the substrate and ECH as the cross-linking agent [101]. The sorption capacity of CS films was 20 mg Ni(II)/g, and the *k* values to Ni(II) were 20.352, 7.138, 56.980, 8.888, 55.150, and 49.249 in the presence of Zn(II), Cd(II), Co(II), Mg(II), Ca(II), and Mn(II) ions, as competing species. The sorption kinetics study indicated that equilibrium was reached after 4 h at pH 4, and the sorption behavior was in good agreement with the PSO model. After five sorption–desorption cycles, the sorption capacity of imprinted CS films for Ni(II) ions still remained at 96.1%.

In the last decade, Ni(II)-imprinted materials with remarkable sorption performances have been also prepared using CS as a functional linear polymer. Thus, macroporous Ni(II)-imprinted CS foams were prepared using sodium bicarbonate and glycerine as porogens [102]. The prepared sorbent achieved a maximum sorption capacity of 69.93 mg Ni(II)/g at pH 6, 313 K, and 2 h contact time. The sorption behavior of the CS foam was best described by PSO and Langmuir monolayer sorption models. The selectivity coefficients for Ni(II) separation from mixtures with Co(II) and Mn(II) ions were 3.63 and 3.88, respectively. The Ni(II) ions sorption capacity of CS foams decreased by about 18% after five successive cycles, when 0.1 M EDTA solution was used as eluent.

Magnetic CS/PVA beads, having improved adsorption capacity and selectivity for Ni(II) ions, have also been fabricated [103]. The maximum equilibrium adsorption capacity was reached within 6 h and the magnetic CS/PVA beads absorbed about 500 mg Ni(II)/g, at 298 K. In column experiments, the magnetic CS/PVA beads exhibited selectivity for Ni(II) recovery from multi-HMIs wastewater. Selectivity coefficients of 15.05, 23.06, and 18.25 were determined for Ni(II) separation from its binary mixtures with Cu(II), Ag(I), and Zn(II), respectively. Kinetic data and adsorption isotherms were well fitted by PSO kinetic and Langmuir isotherm models, respectively. The adsorption capacity was preserved for ten cycles of sorption–desorption, which indicates that the magnetic CS/PVA beads have good efficiency for repeated use. Ni(II)-IPs based on MWCNT, using CS as a bio-based polymer, AA as the functional monomer, and MBAAm as the cross-linker were also designed by inverse suspension polymerization [104]. The maximum sorption capacity was 19.86 mg Ni(II)/g at pH 6, and the selectivity coefficients for Ni(II) recovery in mixtures with Pb(II) and Cu(II) ions were 13.09 and 4.42, respectively. The sorption data were best fitted by the Freundlich isotherm and PSO kinetic models, demonstrating that the process was mainly multilayer chemosorption. In another study, a mesoporous melamine/CS/activated carbon Ni(II)-imprinted biocomposite sorbent with good selectivity toward Ni(II) ions was fabricated [105]. A maximum theoretical sorption capacity of 109.86 mg Ni(II)/g was calculated using the Langmuir model. Selectivity coefficient values of 3.13, 4.48, 3.72, and 2.51 for Ni(II) toward Zn(II), Cd(II), Cu(II), and Pb(II), respectively, were determined. Furthermore, the melamine/CS/activated carbon sorbent presented high reusability after five sorption–desorption cycles.

Magnetic Ni(II)-imprinted CS nanoparticles have been successfully prepared and used for the selective adsorption and separation of Ni(II) ions from wastewaters [107]. The prepared magnetic ion-imprinted CS nanoparticles reached a maximum sorption capacity of 18.5 mg Ni(II)/g, and the time required for establishing the sorption equilibrium was less than 1 h. Thermodynamic results revealed the predominantly endothermic sorption process of Ni(II) ions onto magnetic ion-imprinted CS nanoparticles, and could be well described by Langmuir isotherm and PFO kinetic models. The *k* values of magnetic ion-imprinted CS nanoparticles for the removal of Ni(II) from binary systems with Cu(II) or Zn(II) were 3.02 and 14.35, respectively. Moreover, the sorption capacity of the magnetic IIPs was diminished by about 10% after the fifth adsorption–desorption cycle.

The PMAA/diphenylcarbazide IIPs synthesized by Zhou et al., by bulk polymerization demonstrated remarkable sorption capacity toward Ni(II) ions [112]. The adsorption equilibrium was reached within 30 min and followed the Freundlich isotherm and PSO kinetic models. The maximum adsorption capacity of PMAA/diphenylcarbazide IIPs was 86.3 mg Ni(II)/g at pH 7. In the presence of Na(I), K(I), Mg(II), Ca(II), Ba(II), and Al(III), the *k* values of the imprinted PMAA/diphenylcarbazide composites to Ni(II) ions were 2.107, 3.079, 5.333, 2.436, 1.775, and 3.908, respectively. Desorption of Ni(II) was successfully performed with 0.1 M HCl and the sorbent was reused in five sorption–desorption cycles.

Supermacroporous ion-imprinted cryogels based on PHEMA and MAH as functional monomers endowed with excellent sorption and selective abilities toward Ni(II) ions were prepared by combining IIT with the cryogelation technique [108]. The adsorption kinetics of these cryogels for Ni(II) ions was relatively fast, the equilibrium of sorption being achieved within 60 min. The maximum adsorption capacity of the cryogels at pH 6.5 was 5.54 mg/g, being maintained almost unchanged even after ten cycles of sorption–desorption. The *k* values of PHEMA-MAH cryogels for Ni(II) ions in the presence of Fe(III), Zn(II) or Cu(II) as interfering ions were 4.3, 3.6, and 4.2, respectively.

Environmental-friendly Ni(II)-imprinted magnetic nanomaterials have been synthesized by polymerization of PVA on the surface of bentonite/CoFe_2_O_4_/SiO_2_ particles [109]. The adsorption capacity of Ni(II) by the magnetic bentonite/CoFe_2_O_4_/SiO_2_/PVA nanocomposites increased gradually with time, and reached the adsorption equilibrium at 60 min. The kinetic and isotherm experimental data were well fitted by the PFO and Langmuir models. The *k* values toward Ni(II) in the presence of Cd(II), Zn(II), or Cu(II), as competing ions were 2.149, 2.507, and 3.768, respectively. Nitric acid (0.1 M) was the best desorbing agent for Ni(II). These nanocomposites were successfully reused in five sorption–desorption cycles.

Ni(II)-imprinted ALG beads with remarkable selectivity and affinity toward Ni(II) ions were also prepared [110]. The sorption capacity of imprinted ALG beads was 352.14 mg Ni(II)/g at pH 7 and 301 K. The equilibrium data were well fitted by Langmuir and Freundlich isotherms. The *k* values were 6.38, 6.62, and 7.10, respectively, when Ni(II) ions were separated from their binary mixture with Cu(II), Co(II), and Zn(II) ions. Furthermore, these Ni(II)-imprinted ALG beads exhibited good reusability after five sorption–desorption cycles (*RE* remained around 95%).

Long et al., prepared Ni(II)-IP by the precipitation polymerization technique of 2-(allylmercapto)nicotinic acid (ANA) (as functional monomer) and EGDMA (as the cross-linking agent) in the presence of Ni(II) ions as the template, and AIBN as the initiator [111]. The FTIR and XPS analyses revealed that the sorption of Ni(II) ions by the IIPs was based on the chemisorption. The maximum Ni(II) adsorption capacity of the IIPs was 38.85 mg/g at pH 6 and 303 K, the adsorption equilibrium being reached in less than 20 min. The sorption kinetic was well fitted by PSO kinetic, whereas the equilibrium data were well described by the Langmuir isotherm model. Moreover, the Ni(II)-IP showed excellent *k* values of 33.63, 219.59, 4.23, 16.89, and 32.74 in binary systems containing Ni(II) and Zn(II), Mg(II), Co(II), Cd(II), or Cu(II), respectively. The sorbent was easily regenerated and reused in five sorption–desorption cycles, when 82.7% of its initial sorption capacity was preserved.

### 3.6. Iron-Imprinted Polymers

Various iron-imprinted polymer systems have been elaborated in the last decades mainly as solid-phase extractors for pre-concentration or removal of iron from the environmental water (see Table S4) [113,114,115,116,117,118] or food samples [116,119]. The surface molecular imprinting using CS-succinate for the complexation of Fe(III), followed by cross-linking with ECH has been adopted for the preparation of Fe(III)-IP taking into account its advantages such as high selectivity and the absence of mass transfer difficulties [113]. The selectivity for Fe(III) ions of this IIP system has been demonstrated by the adsorption of Fe(III) in competition with Mn(II) and Al(III), in batch mode. The values of *k* found for an Fe(III)/Mn(II) binary system were 3.09 and 12.63 for NIP and IIP, respectively, while the value of *k’* was four. In the case of an Fe(III)/Al(III) binary system, the values of *k* were 1.16 and 5.96 for NIP and IIP, respectively, while the value of *k’* was 5.13. The time necessary to reach the equilibrium of sorption was relatively short (about 80 min).

**Figure 11:**
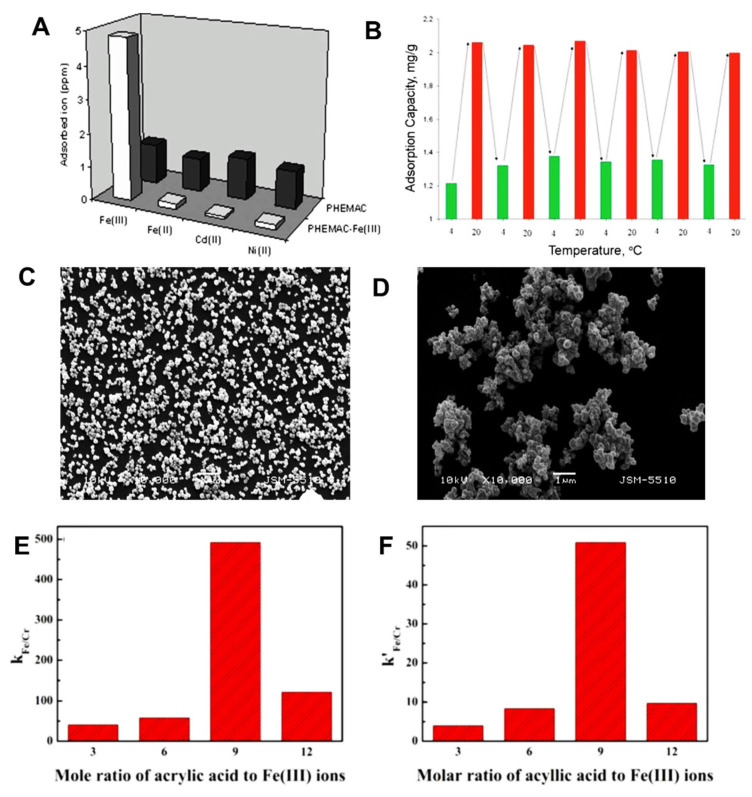
(**A**) Adsorbed template and competitive ions onto PHEMAC and PHEMAC-Fe(III) monoliths for Fe(III)/competitive ion pairs (reproduced with permission from Ref. [114]. Copyright 2010 John Wiley and Sons). (**B**) Adsorption of Fe(III) at two temperatures (reproduced with permission from [115]). SEM images of NIP (**C**) and Fe(II)-IIP (**D**) particles; Mag 10000x (reproduced with permission from Ref. [119]. Copyright 2017 Elsevier). Effect of the mole ratio between AA and Fe(III) on the selectivity factor (**E**) and relative Selectivity factor (**F**) (reproduced with permission from Ref. [120]. Copyright 2019 Elsevier).

Denizli et al., adopted the monolithic preparation of Fe(III)–IIP by in situ polymerization of HEMA in the presence of Fe(III) pre-complexed with *N*-methacryloyl-(l)-cysteine methyl ester (MAC) (PHEMAC-Fe(III)), using acetonitrile as porogen, followed by the template removal with EDTA [114]. The specific surface area of the monolith was 35.2 m^2^/g. The selectivity of PHEMAC-Fe(III) mesoporous sorbent for Fe(III) was demonstrated by the competitive sorption at the equilibrium of Fe(III) in the presence of Fe(II), Cd(II), or Ni(II), in binary systems [114]. It was found that the ion cavities formed in the PHEMAC-Fe(III) preferentially adsorbed Fe(III) in competition with Fe(II), Cd(II), or Ni(II), supported by the values of *k’* found comparing PHEMAC-Fe(III) with PHEMAC, which were 24.8, 37.0, and 59.7 for Fe(III)/Fe(II), Fe(III)/Cd(II), and the Fe(III)/Ni(II) ion pairs, respectively. Figure 11A shows that the highest amount of Fe(III) was adsorbed onto PHEMAC-Fe(III) in comparison with PHEMAC, in the presence of competing ions, with this difference being attributed to the generation of recognition sites during the synthesis of monolith [114].

After ten adsorption–desorption cycles, the adsorption capacity of the PHEMAC-Fe(III) column was found at the 90% level from the initial, which supports a high level of recyclability for this monolith.

An interesting approach for the preparation of Fe(III)-IIP was reported by Karabörk et al., who prepared thermoresponsive poly(methacryloyl antipyrine (MAAP)-Fe(III)-NIPAAm (TIIP) by bulk polymerization [115]. The formation of the pre-complex between Fe(III) and MAAP is presented in Table S5. The optimum pH for the sorption of Fe(III) onto TIIP was 4.0, and the optimum temperature for the adsorption of template was 20 °C. Adsorption of template ion onto TIIP and NIP particles in competitive conditions in binary systems, i.e., Fe(III)/Al(III), Fe(III)/Zn(II), Fe(III)/Co(II), Fe(III)/Mn(II), Fe(III)/Ni(II), and Fe(III)/Cu(II), was much higher than that of any competitor ion due to the pre-designed geometry of cavities; the ion affinity being as follows: Fe(III) > Ni(II) > Co(II) > Mn(II) > Zn(II) > Al(III) > Cu(II). The adsorption capacity of the TIIP particles for Fe(III) ions was also much higher than that onto NIP particles [115]. The values of *k’* were 83.4, 66.4, 62.5, 41.2, 16.3, and 14.8 when the competitor ion was Ni(II), Co(II), Mn(II), Zn(II), Al(III), and Cu(II), respectively. The adsorption–desorption cycles of the template ion as a function of temperature are presented in Figure 11B. As Figure 11B shows, the amount of template adsorbed at 20 °C was almost the same during the six adsorption cycles, the desorbed amount at 4 °C being also similar for each adsorption–desorption cycle. From these results, it can be said that the adsorption and desorption of Fe(III) ions can be performed by temperature switching with this thermoresponsive sorbent.

In another approach, Fe(III)-IIP was synthesized by precipitation polymerization with gallic acid as the ligand for Fe(III), using MMA as the functional monomer, EGDMA as the cross-linker, AIBN as the initiator, and acetonitrile as porogen. Relative selectivity values in competitive conditions were 3.41 and 0.707, in Fe(III)/Cr(III) and Fe(III)/Ag(I) binary systems, respectively [117]. This sorbent was successfully tested for the pre-concentration of Fe(III) from a lake water sample.

For the determination of Fe(III) and total Fe(II) + Fe(III) in wine samples, Mitreva et al., have developed a novel Fe(II)-IIP sorbent synthesized by precipitation copolymerization of 4-VP as functional monomer, TMPTMA as cross-linker and 2,2′-bipyridyl as a specific ligand for Fe(II). The scheme for the Fe(II)-IIP preparation is presented in Table S5 [119]. Differences between the surface structure and morphology between NIP and Fe(II)-IIP particles have been evidenced by SEM (Figure 11C,D). As can be seen in Figure 11C, the NIP particles are almost spherical and uniform, while the Fe(II)-IIP particles look like large aggregates of irregular particles (Figure 11D). The authors have attributed the rough surface of the IIP sorbent to the cavities generated on the sorbent surface after the template removal. The adsorption selectivity toward Fe(II) ions onto the Fe(II)-IIP sorbent has been investigated at pH 7, in batch mode, in the presence of interfering ions, such as Cd(II), Cu(II), Fe(III), Mn(II), Pb(II) and Zn(II), which often coexist with Fe(II) in wine samples [119]. The values of relative selectivity of Fe(II) in competition with these ions ranged between 19.5 for Mn(II) as competitor and 67.5 for Pb(II) as an interfering ion. The lowest selectivity coefficient has been reported for Fe(III) ions (*k* = 2.04) and this was attributed to the similar stoichiometry of the complexes with 2,2′-bipyridyl. To selectively separate Fe(II) from Fe(III), the authors have used fluoride anions as a mask because Fe(III) ions form stable complexes with F^−^ [Fe(III)F_4_^−^]. In this way, the selectivity constant between Fe(II) and Fe(III) dramatically increased up to 1584.4.

Another important application of Fe(III)-IIPs sorbents is focused on the removal of trace Fe(III) from Cr(III)-containing solutions [120,121]. The presence of iron impurities during the fabrication of Cr(III) products has negative effects on the tanning properties of the basic chromium sulfate and on the coloring efficiency of chromium oxide green [120,121]. To solve this problem, Fe(III)-IIP have been prepared either by cross-linking polymerization of AA pre-complexed with Fe(III) using EGDMA as cross-linker, in bulk, followed by the removal of the template with HCl (scheme in Table S5) [120] or by using bis(2-methacryloxyethyl) phosphate (BMAOP) as a ligand for Fe(III) followed by polymerization and leaching of a template (scheme in Table S5) [121]. According to the first approach, the functional monomer interacted directly with the template, and therefore, the optimum mole ratio between AA and Fe(III) was determined first and found to be 1:6, the adsorption capacity being maximum at this mole ratio. However, the selectivity toward Fe(III) in competition with Cr(III) has been the main goal of this study. As can be seen in Figure 8E,F, the highest selectivity factor and the highest relative selectivity factor have been found at a mole ratio of AA to Fe(III) of 9:1, due to the better matching between AA and Fe(III) at this ratio [120]. The IIP sorbent prepared with BMAOP as a ligand for Fe(III), in a mole ratio of 3:1 (Table S5), displayed a high selectivity in the removal of trace Fe(III) ions from high-concentration basic chromium sulfate solutions, even in the presence of other interfering ions [121]. Thus, the relative selectivity factors found for the adsorption of Fe(III) in binary systems with Cr(III), Co(II), Ni(II), Cu(II), or Zn(II) were as follows: 2.44, 4.38, 4.17, 1.78, and 4.15 for Fe(III)/Cr(III), Fe(III)/Co(II), Fe(III)/Ni(II), Fe(III)/Cu(II), and Fe(III)/Zn(II) ion pairs [121].

Iron-imprinted nanochelators have been fabricated and tested for the removal of iron in excess from synthetic gastric fluid [122,123,124]. Using *N*-methacryloyl-(l)-glutamic acid (MAGA), reach in carboxyl and amide groups, as a ligand for Fe(III), Karaboç has developed a novel Fe(III)-IIP sorbent as nanoparticles by surfactant-free emulsion polymerization of HEMA in the presence of Fe(III)/MAGA [123]. A specific surface area and a mean diameter of Fe(III)-IIP nanoparticles of 895 m^2^/g and 95.3 nm, respectively have been reported. For the selectivity test in an aqueous solution, Cd(II), Co(II), Cr(III), Ni(II), Pb(II), Zn(II), and Mn(II) have been used as competitor ions, in batch mode. Fe(III)-IIP nanoparticles displayed a high affinity for Fe(III) in an acidic environment, the optimum pH being 4.0. Comparing the selectivity coefficients found for the adsorption onto Fe(III)-IIP sorbent with those on NIP, the imprinting effect has been obvious by the very high relative selectivity coefficients. The values of *k’* reported for Fe(III)/Cd(II), Fe(III)/Co(II), Fe(III)/Cr(III), Fe(III)/Ni(II), Fe(III)/Pb(II), Fe(III)/Zn(II), and Fe(III)/Mn(II) ion pairs were: 196.5; 241.2; 218.3; 191.5; 164.7; 147,2 and 252.8. The high values of *k’* have been assigned to the successful distribution of the binding sites by the incorporation of MAGA [123]. Information about the in vivo performances of Fe(III)-IIP poly(HEMA-MAGA) nanoparticles have been obtained by adsorption tests in a medium that mimics the intestinal fluid of the small intestine. It was found a higher sorption capacity from intestinal fluid than in aqueous solution (206.4 mg Fe(III)/g IIP). A similar strategy has been adopted by Sangu et al., who have prepared Fe(III)-IIP using MAGA as a functional monomer chelator for the preparation of pre-complex of Fe(III), HEMA, and EGDMA as the cross-linker [124]. The selectivity for Fe(III) of IIP nanoparticles has been evaluated in an aqueous solution in the presence of three interfering ions, Zn(II), Cr(III), and Al(III). The authors have reported the values of *k’* to be 8.85, 4.91, and 6.29, respectively. Removal of Fe(III) in simulated gastric fluid with pH 1.2 was 97.16%, while that of the competitor ions Zn (II), Cr (III), and Al(III), in the same medium, was 13.00%, 5.41%, and 5.89%, respectively. This IIP nanochelator was very stable during the successive adsorption–desorption cycles, the preservation of the removal capacity being 97.13% at the end of ten cycles [124].

### 3.7. Mercury-Imprinted Polymers

Mercury is one of the most toxic and accumulative heavy metal ions, whose identification, pre-concentration, and removal ask for sorbents with high selectivity and sorption capacity. Various Hg(II)-IIP sorbents have been developed for this purpose [125,126,127,128,129,130,131,132,133,134] (Table S6), many of them having the ability to discriminate between inorganic Hg(II) and organic CH_3_Hg(I) ions [125,127,128]. Thus, Dakova et al., have prepared Hg(II)-IIP sorbents by copolymerization of MAA with TMPTMA in the presence of either a Hg(II)-1-(2-thiazolylazo)-2-naphtol complex [125] or a Hg(II)-1-pyrrolydine-dithiocarboxylic acid complex [127] and used them successfully in the determination of Hg(II) and CH_3_Hg(I) in water samples. For this purpose, the selective determination of Hg(II) was performed first, and then the total Hg(II) was measured in digested water samples, and the CH_3_Hg(I) content was the difference between these two measurements. In the selectivity evaluation of Hg(II), the highest selectivity coefficient for Hg(II) was found in the presence of CH_3_Hg(I). The authors have attributed this difference to the large size of CH_3_Hg(I), which hindered the ion diffusion and finally led to the highest selectivity coefficients. The high values of the *k′* (143–1100 times higher for imprinted compared with blank sorbents, Table S6) [127] support the high efficiency of these sorbents in selective identification of Hg(II) speciation.

**Figure 12:**
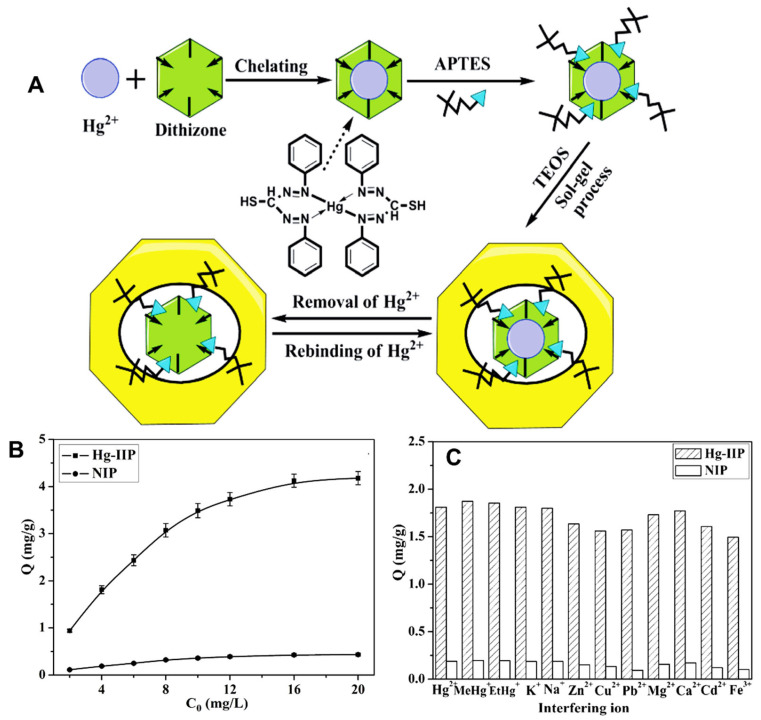
(**A**) Schematic illustration of Hg(II)-IP preparation; (**B**) Adsorption isotherm curves of IIPs and NIPs for Hg(II) in aqueous solutions; (**C**) Adsorption capacities for Hg(II) in the presence of 4 mg/L Hg(II) and 40 mg/L of other metal ions (experimental conditions: IIPs, 20 mg; V, 10 mL; pH, 7.0). (Reproduced with permission from Ref. [128]. Copyright 2011 Royal Society of Chemistry).

A different approach for the synthesis of Hg(II)-IIP sorbents was developed by Zhang et al., by a sol–gel process with the DZ-Hg(II) complex as the template, 3-aminopropyltriethoxysilane (APTEOS) as the functional monomer, and TEOS as the cross-linker, as presented in Figure 12A [128].

The amount of Hg(II) ions adsorbed per unit mass of IIPs increased with the increase in the initial concentrations of Hg(II), the experimentally obtained maximum adsorption capacity of Hg-IIPs being about 10 times higher than that of NIPs (Figure 12B). Figure 12C shows that the interfering ions did not significantly reduce the capacity of the IIPs for Hg(II) pre-concentration even when their concentration was 10 times higher. The selective adsorption of Hg(II) from binary mixtures, including Hg(II)/CH_3_Hg(I), Hg(II)/EtHg(I), Hg(II)/Zn(II), Hg(II)/Cd(II), and Hg(II)/Pb(II), by Hg(II)IIPs was investigated and found the values of *k* in the range of 19–34 [128].

Based on the selectivity of Hg(II) adsorption in competition with CH_3_Hg(I) and C_2_H_5_Hg(I), the authors determined the content in organic mercury species, in a similar way to that presented above [125,127]. The applicability of the Hg(II)-IIP SPE method for the analysis of inorganic and organic mercury species was demonstrated by the analysis of environmental waters and biological samples (human hair and fish meat).

A novel strategy for the preparation of Hg(II)-IIP sorbents was developed by Hajri et al., by the preparation of a Schiff base ligand from 2-pyridine carboxaldehyde and 4-amino-3-hydroxybenzoic acid (HPB), which was linked with the –NH_2_ groups of CS by the carbodiimide (EDC)/*N*-hydroxysuccinimide (NHS) strategy to produce the amide CS derivative [132]. After the coordination with Hg(II), the CS chains have been cross-linked with GA. Selectivity in the sorption of Hg(II) in multicomponent systems containing Hg(II), Pb(II), Co(II), Zn(II), Cu(II), and Cd(II) ions was evaluated, and found the values of the selectivity coefficients for the coexisting ions in the range 14.45–25.77, and the relative selectivity coefficients, *k’* >> 1. Magnetic Hg(II)-IIP sorbents have been designed by Najafi et al., for the determination of low levels of Hg(II) in various fish samples, the sorbent performances being presented in Table S6 [134]. Organic mercury as CH_3_Hg(I) ions, which have a higher toxicity than Hg(II) because of their liposolubility, have also been used as the template in the synthesis of IIP as SPE sorbents for the selective sorption of methylmercury from water [135,136]. For example, Mesa et al., have designed CH_3_Hg(I)-IIP sorbents as AA-EGDMA copolymers using 2-mercaptobenzoimidazole and 2-mercaptobenzothiazole (MBT) as ligands for CH_3_Hg(I) ions and investigated their performances in the sorption/pre-concentration of CH_3_Hg(I) spiked in river and tap water samples [136]. In the optimum conditions, the best results were obtained with CH_3_Hg(I)-IIP sorbent prepared with MBT as the ligand. Using this sorbent, the level of CH_3_Hg(I) ions recovery from spiked water samples ranged from 84% to 95%.

### 3.8. Chromium-Imprinted Polymers

Separation and preconcentration of Cr(III) from water by Cr(III)-IIP sorbents based on ion-imprinted-functionalized mesoporous SiO_2_ have been lately explored (Table S7) [137,138,139,140,141,142]. Liu et al., have developed a Cr(III)-IIP by one-step sol–gel reaction using APTEOS as a coupling agent and a Cr(III) surface imprinting on the surface of a mesoporous SiO_2_ (SBA-15) [137]. This novel sorbent has been successfully used in selective adsorption, pre-concentration, and separation of Cr(III) in competition with Cr(VI) and in the quantitative determination of Cr(III) and Cr(VI) after the reduction of Cr(VI) with hydroxylamine.HCl. After the competitive sorption of Cr(VI), Sr(II), Co(II), Ni(II), Cu(II), Pb(II), and Zn(II), by Cr(III)-IIP and NIP sorbents, the following values for *k’* were reported: 45.67, 197.52, 39.35, 92.36, 20.12, 4485.84 and 83.35, respectively [137].

**Figure 13:**
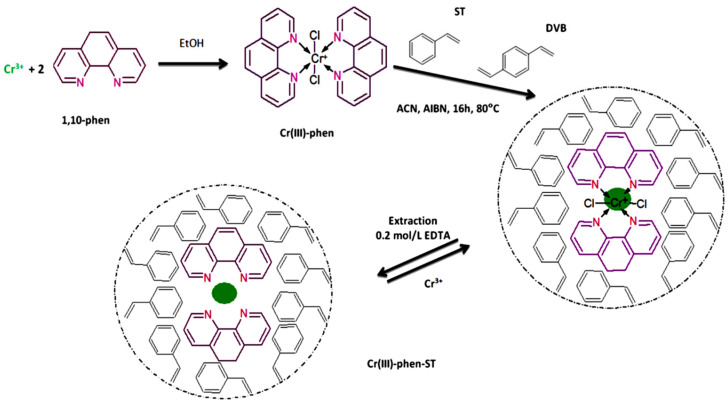
Schematic illustration of the imprinting process for preparation of the Cr(III)-phen-ST-IP (reproduced with permission from Ref. [140]).

In another approach, the surface of SBA-15 was functionalized with DZ as the ligand, and then the imprinting of Cr(III) was performed by the cross-linking polymerization of MAA with EGDMA in the presence of Cr(III) ions on the surface of SBA-15-DZ [138]. The adsorption properties, selectivity, and reusability of the IIP as SPE are strongly influenced by the nature of the metal-complexing ligand, functional monomer, cross-linker, porogen, and initiator [138,139,140]. Starting from the advantages of the dynamic mode, Trzonkowska et al., have explored the performances of the Cr(III)-IIP prepared by bulk method with different functional monomers, cross-linkers, and initiators using the Cr(III)-1,10-phenantroline complex as a template [139,140]. The authors found that the nature of the functional monomer, MAA or ST affected the amount of Cr(III) imprinted, the mechanical properties of the sorbent, and the sorption capacity toward Cr(III) ions, the IIP prepared with ST as monomer and AIBN as initiator having a higher selectivity for Cr(III) than that prepared with MAA. The Cr(III)-IIP could be reused up to 100 sorption–desorption cycles. The selectivity of the sorbent toward Cr(III) in the presence of Cu(II), Mn(II), and Fe(III) ions increased when the pre-complex between Cr(III) and 1,10-phenantroline was used in the synthesis of the copolymer ST-4-VP and DVB [140]. The scheme for the bulk synthesis of Cr(III)-1,10-phenantroline (phen)-ST sorbent is presented in Figure 13.

The as-prepared Cr(III)-IIP has been used in the determination of Cr(III) ions in tap water and infusion of green tea.

Elsayed et al., designed an efficient sorbent for selective separation/removal of Cr(III) by the SPE process by preparation of an azo dye functionalized CS, which was complexed with Cr(III), followed by the cross-linking of CS with glyoxal [141]. In competition with Ni(II), Co(II), Al(III), Eu(III), Cu(II), and Fe(III), in binary systems, the relative selectivity for Cr(III) increased in the order: 9.37, 9.38, 9.85, 10.98, 13.26, and 15.15, respectively. Cr(III)-ion imprinted membranes (Cr(III)-IIMs) were prepared from ALG and PVA as film-forming polymers, poly(ethylene glycol) (PEG) as porogen, and ALG coated gold nanoparticles (ALG-AuNPs) as the cross-linker and Cr(III) ions as the template [142]. Under the optimum conditions of sorption, the adsorption of Cr(VI) was <5%, thus allowing the selective measurement of Cr(VI) after the adsorption of Cr(III).

**Figure 14:**
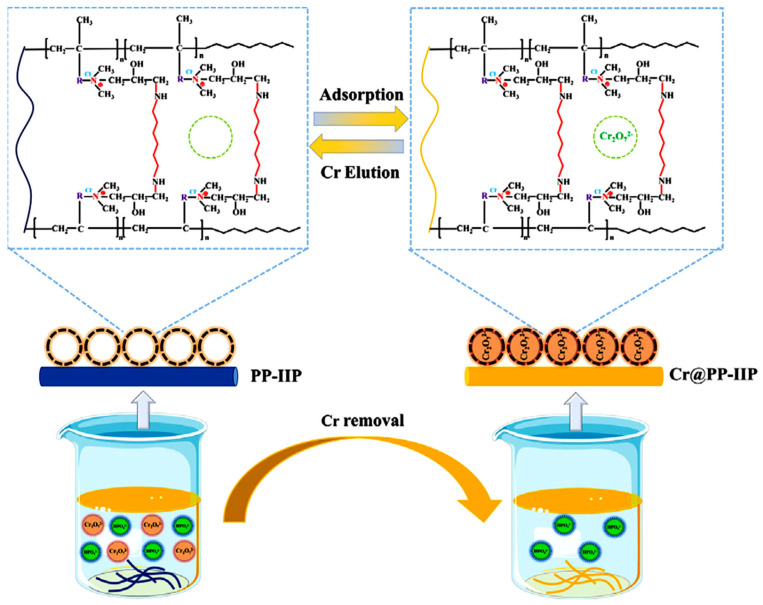
Schematic illustration of the action of PP-IIP in the removal of Cr(VI). Green circle represents Cr_2_O_7_^2−^ anions (reproduced with permission from Ref. [144]. Copyright 2021 Elsevier).

Unlike Cr(III) ions, which have a role as rare essential elements in the human body because they are used in the metabolism of glucose, Cr(VI) is a very toxic, carcinogenic, and mutagenic element. Cr(VI) is found in water as oxyanions as a function of concentration and water pH. The removal of Cr(VI) oxyanions from water is a stringent concern that asks for very efficient and selective sorbents (see Table S7) [143,144,145,146,147,148,149,150,151,152,153,154,155]. For example, fast removal of Cr(VI) from aqueous solution was achieved using the Cr(VI)-IIP prepared with 4-VP and DMAEMA as functional monomers, EGDMA as the cross-linker, AIBN as the initiator, and a binary porogen mixture, by thermally induced polymerization [143]. The sorption kinetic of Cr(VI) ions was very fast in the acidic range. As was already mentioned above, in the case of surface IIPs, the recognition sites are placed on or near the surface of the substrate, making the selective sorption of the template very fast [144,145,147,148]. Surface imprinting of Cr(VI) as the template on polypropylene (PP) as the substrate was adopted by Kong et al. [144]. As seen in Figure 14, the pre-grafting of DMAEMA onto the surface of PP, under a high electron beam irradiation, occurred first followed by the quaternization of a tertiary amine with ECH and cross-linking with 1,6-diaminohexan in the presence of Cr_2_O_7_^2−^ as the template [144]. The benefits of the quaternary ammonium groups on the performances of the novel sorbent were demonstrated by the extension of the pH domain where the maximum sorption capacity was in the range of pH 2 to 4, with a slow decrease when pH increased up to 7.

CS-graphene nanocomposite electrodeposited onto an Au electrode and cross-linked with GA was designed and tested as a sensor for Cr(VI) ions [147]. This sensor was able to detect 1 µM Cr(VI), in tap water and river water, with a high selectivity toward Cr(VI) over the interfering ions such as Zn(II), Co(II), Cu(II), Ni(II), Mn(II), MnO_4_^−^, Cr_2_O_4_^2−^, and Mn_2_O_4_^2−^, indicating that it can be a powerful tool for the analysis of various water samples. After nine sorption–desorption cycles, the electrochemical response declined by 15%, indicating an acceptable recyclability of the Cr(VI)-IIP sensor. Luo et al., modified PP fibers by plasma-mediated grafting of AA, which was then amidated with TETA. Cross-linking with GA in the presence of HCrO_4_^−^ ions followed by leaching the HCrO_4_^−^ ions led to Cr(VI)-IIP sorbent [148].

Magnetic Cr(VI)-IP has lately attracted interest in finding sorbents endowed with fast selective sorption and easy to separate from the environment [149,150]. Thus, Hassenpour et al., prepared the first magnetic Fe_3_O_4_ nanoparticles (MNPs) modified with TEOS [149]. The magnetic Cr(VI)-IIP sorbent was prepared through the precipitation copolymerization of 4-VP as a complexing monomer, HEMA as a functional co-monomer, Cr_2_O_7_^2−^ as the template, and EGDMA as the cross-linker. The selectivity studies in the presence of Ni(II), Cd(II), Cu(II), NO_3_^−^, SO_4_^2−^, and PO_4_^3−^, as interfering ions, in binary systems, indicated that the synthesized sorbent had a high selectivity for the Cr(VI) ions in the presence of competing ions [149]. Recently, it was reported that Cr(VI)-IIP sorbent based on (4-VP-*co*-EGDMA), prepared by free-radical precipitation polymerization with POB as the initiator and ethanol/acetone as porogen, selectively removed Cr(VI) ions, at low concentrations, from electroplating industrial water [151]. The pre-complex formed between Cr_2_O_7_^2−^ and 4-VP was prepared first. The optimum parameters for the sorption of Cr(VI) by this sorbent are presented in Table S7. Recovery of Cr(VI) from electroplating industrial waters was >96%.

Xing et al., developed novel Cr(VI)-IIP sorbents having polypyrrole (PPy) dispersed in a cross-linked hydrogel network based on gelatin (Gel) and CS (Gel/CS/PPy) in order to decrease the consumption of reagents during the desorption process of the sorbent [152]. It was found that the electro-assisted desorption of Cr(VI) increased the desorption efficiency by 140%, while the photo-assisted regeneration improved the desorption rate by 19.8%.

Cr(VI)-IP sorbent prepared by precipitation polymerization with 1-VI as the ligand for Cr_2_O_7_^2−^ anions, MAA as the functional monomer, and EGDMA as the cross-linker was prepared and tested for its capacity to selectively adsorb Cr(VI) ions from real water [153]. The *k’* values of Cr(VI)/Cr(III), Cr(VI)/Co(II), Cr(VI)/Cu(II), Cr(VI)/Ni(II), Cr(VI)/NO_3_^−^, Cr(VI)/PO_4_^3−^, and Cr(VI)/SO_4_^2−^ were 1.367, 1.529, 5.294, 5.052, 1.416, 1.482, and 1.527, respectively (see also Table S7).

**Figure 15:**
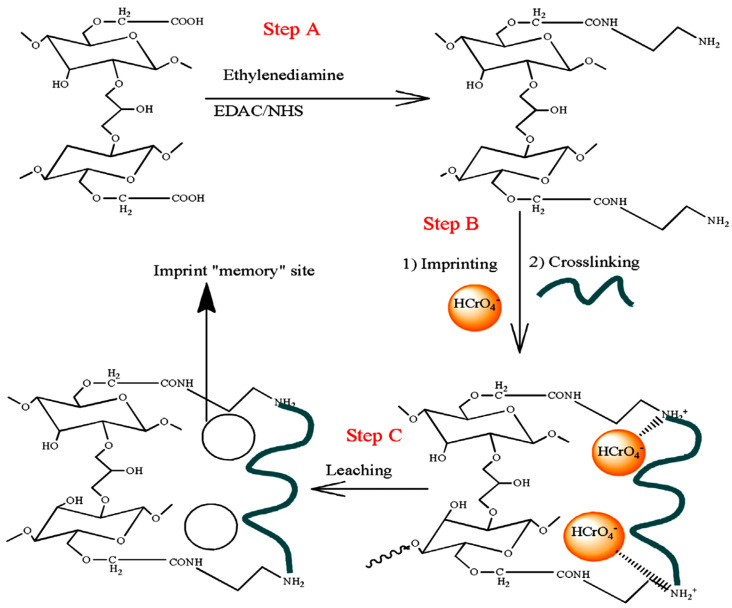
Preparation scheme of CMC/EDA imprinted polymer (reproduced with permission from Ref. [154]. Copyright 2017 Elsevier).

A novel IIP sorbent was prepared by functionalization with ethylene diamine (EDA) of carboxymethyl cellulose (CMC) cross-linked with ECH followed by adsorption Cr_2_O_7_^2−^ as the template, as seen in Figure 15 [154]. In this approach, EDA was acting as a ligand and as the second cross-linker to better stabilize the template.

The values of *k’* for Cr(VI), in binary systems, followed the order: SO_4_^2−^ > PO_4_^3−^ > F^−^ > NO_3_^−^ > Cl^−^. All *k’* values were higher than unity supporting the predilection of the sorbent for Cr(VI) in the presence of interfering anions [154] (see also Table S7). Hu et al., recently reported an interesting study on the role of a cross-linking agent on the polymer template effect taking vinylbenzyltrimethylammonium chloride (VTA) as the functional monomer, EGDMA as the cross-linking agent, and Cr_2_O_7_^2−^ oxyanion as the template ion [155]. A series of IIPs with different mole ratios between the functional monomer and cross-linking agent as well as the corresponding non-imprinted polymers (NIPs) were synthesized for competitive adsorption, kinetic, and elution experiments. This study provides guidance in designing the synthesis of IIP sorbents for the removal of heavy metals from water.

### 3.9. Other Transition Metal Ions-Imprinted Polymers (Mn, Mo, Re, and Ru)

One of the best ways to decrease the level of Mn(II) ions in the environment is their adsorption onto Mn(II)-IIPs (see Table S8) [156,157,158]. For this aim, an interesting approach was recently developed by Aravind and Mathew who fabricated electrochemical sensors for Mn(II) ions by the modification of a platinum electrode using the IIP technique, with and without vinyl functionalized MWCNTs, which come with their high surface area, electrical, chemical, and mechanical stability, as well as easy electron transfer [158]. The functional monomer was MAA and the cross-linker was MBAAm. Information about the performances of this type of sensor is presented in Table S8. To evaluate the selectivity of the Pt-MWCNTs-Mn(II)-IIP sensor, the sorption of Mn(II) in the presence of Co(II), Fe(II), and Cr(III) as competitive ions was investigated. It was found that only Mn(II) ions exhibited a redox peak current response while the other metal ions did not show any current response [158].

Molybdenum exists only in various oxidation states in minerals, but not as a free element. Molybdenum has a small tendency to form a cation in an aqueous solution. Most molybdenum compounds have low solubility in water, but when molybdenum-bearing minerals come in contact with oxygen and water, the resulting oxyanion MoO_4_^2−^ is soluble. This state of Mo(VI) has been used as a template to engineer Mo(VI)-IP sorbents. Mo(VI)-imprinted CS/triethanolamine (TEA) gel beads have been prepared by Zhang et al., using TEA and molybdate solution as a coagulation bath for the formation of CS beads [159]. Performances in the selective sorption of this sorbent can be seen in Table S8. The advantages of magnetic ion-imprinted polymers were exploited by Hassenpour et al., in the fabrication of Mo(VI)-IIP magnetic nanoparticles by the preparation first of SiO_2_-coated Fe_3_O_4_ [160]. MAA and EGDMA were polymerized in the presence of Fe_3_O_4_, and Mo(VI) complexed with NaSCN and tetrabutyl ammonium ions to obtain magnetic Mo(VI)-IP after the removal of Mo(VI) ions [160]. The influence of interfering ions on the adsorption of Mo(VI) ions was evaluated to obtain information about the selectivity of the sorbent toward Mo(VI) ions. As can be seen in Table S8, the Mo(VI)-IP displayed much greater adsorption in the presence of Ni(II), Cd(II), Mn(II), Zn(II), and Cu(II) as competitors [160]. Porous Mo(VI)-IP membranes as selective adsorbents for Mo(VI) were prepared from ALG cross-linked with GA, in the presence of PVA as a thickener, PEG as porogen, and H_3_Mo_7_O_24_^3–^ as the template and cross-linker [161]. The selectivity for Mo(VI) has been tested in multicomponent systems containing Re(VII), Cu(II), Fe(III), Zn(II), and Mn(II) as interfering ions. The results demonstrated a poor adsorption capacity for its coexisting ions at pH 3 and high *k’* values for Mo(VI) (47.16) (Table S8).

Rhenium is one of the scarcest transition metals and is very important for its applications in high-tech domains such as aerospace, electronics, and nuclear energy, and therefore its extraction and recovery have attracted the interest of scientists all over the world [162,163,164]. It was demonstrated that ReO_4_^−^ ion exists irrespective of the acidity of the solution [162,163]. Because of the high cost of rhenium, and taking into account the ionic radius of MoO_4_^2−^, which is 3.23 Å, similar to that of ReO_4_^−^ (3.3 Å), Xiong et al., designed anionic imprinted sorbents using MoO_4_^2−^ as the template, and either EDA-functionalized CS (I-EDA-CS) [162], or CS-mesoporous SiO_2_ composite (I-CTS-KIT-6) [163]. In preliminary tests, the imprinted sorbents using ReO_4_^−^ as the template (Re-EDA-CS or RE-CTS-KIT-6) were also prepared and the sorption capacities were compared with those of the sorbents imprinted with MoO_4_^2−^. The similar results found by the authors constituted the basis for the substitution of ReO_4_^−^ with MoO_4_^2−^ ions as the template. The authors have successfully used the novel sorbents in the selective adsorption of ReO_4_^−^ in multicomponent systems with Cu(II), Fe(III), Zn(II), and Mn(II) as competitors. The adsorption performances are presented in Table S8.

### 3.10. Ion-Imprinted Polymeric Materials for Selective Extraction of Precious Metals

Precious metal ions pose a high interest because of their high economic value and large-scale utilization in the jewelry industry, in electronics development, as catalysts, in metal plating, and in pharmaceutical fields. In this respect, numerous IIP sorbents have been developed and investigated over the last decade as efficient solutions to selectively recover precious metal ions, such as Ru(III), Ag(I), Au(III), and Pd(II), from wastewaters (see Tables S8 and S9).

#### 3.10.1. Ruthenium-Imprinted Polymers

**Figure 16:**
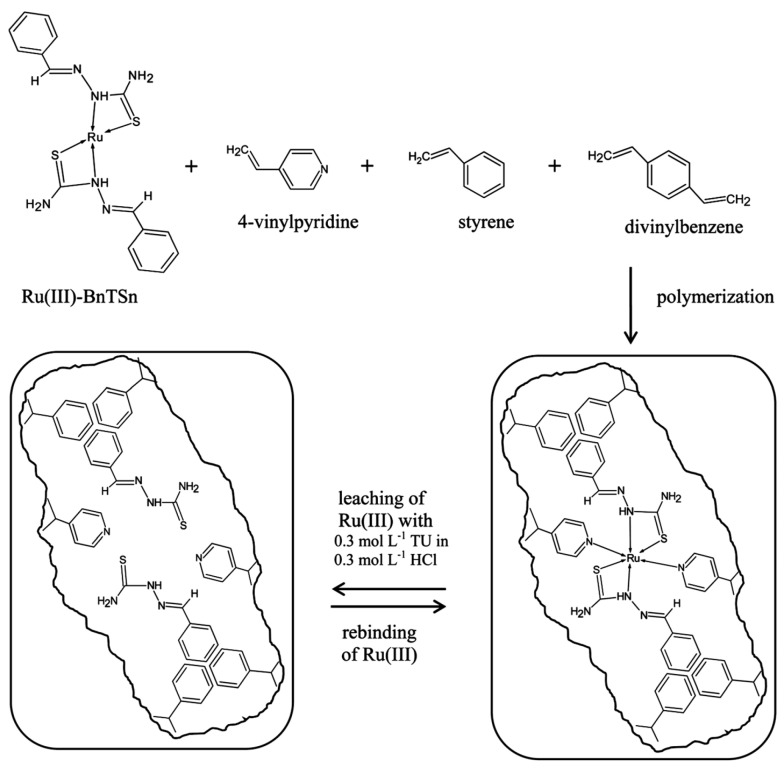
Schematic illustration of the preparation of the Ru–BnTSn (reproduced with permission from Ref. [165]. Copyright 2009 RSC Publishing).

Ruthenium belongs to the platinum group and is widely used in industries such as chemical (catalyst in the production of acetic acid and ammonia), electronic, and solar cells [165,166,167,168]. Ru is employed as a hardener in high-density alloys with Pt group metals utilized in the production of corrosion-resistant electrical connectors and usually coexists with Pt group metals [168]. Some Ru(III) complexes have displayed anticancer activity and have been investigated for this purpose [166,168]. These are the main sources that cause the presence of Ru(III) in the environment. Among the most effective techniques for the removal/recovery of Ru(III), the SPE using smart sorbents such as those obtained by IIT is preferred [165,166,167,168,169]. Zambrzycka et al., developed the synthesis of IIPs as selective sorbents for the separation of Ru(III) from the environment using either 4-VP and ST cross-linked with DVB [165] or MAA/AAm as functional monomers and EGDMA as the cross-linker by the bulk polymerization technique [166]. The complexes of Ru(III) with benzaldehyde thiosemicarbazone (Figure 16) [165] or with 2-thiobarbituric acid [166] have been used as templates for Ru-IIPs. Sorption performances established in dynamic mode can be seen in Table S8. It was found that by using longer and narrower columns, the retention efficiency increased up to 20%.

Monier et al., prepared Ru-IIP using CS functionalized with pyridyl-thiourea (PTU) to form a polymer complex with RuCl_3_, which was cross-linked with ECH [168]. After leaching the Ru(III) ions, the Ru-PTCS sorbent was obtained having Ru(III) recognition sites. The sorption performances and information about the selective sorption of Ru(III) in multicomponent systems are presented in Table S8. In a recent approach, a temperature-sensitive block polymer PDEA-*b*-P(DEA-*co*-AM) was prepared first by solution polymerization and then used in the preparation of a smart Ru(III)-imprinted polymer (Ru-IIP) by cross-linking polymerization of acrylamide (AM) with MBAAm in the presence of PDEA-*b*-P(DEA-*co*-AM) and RuCl_3_ [169] (Table S8). When the Ru-IIP lost its efficiency in the separation of Ru(III), the authors investigated its activity as a catalyst in the reaction between nitrobenzene and benzyl alcohol to form imines [169].

#### 3.10.2. Silver-Imprinted Polymers

**Figure 17:**
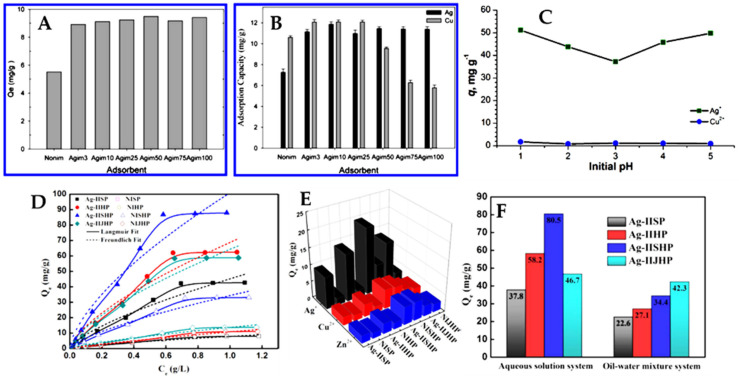
(**A**) Ag(I) sorption capacity of CS hydrogels with different imprinting ratios (Nonim = non-imprinted hydrogels; Agim3 to Agim100 = hydrogels with different Ag(I) imprinting mass ratios). (Reproduced with permission from Ref. [170]. Copyright 2012 American Chemical Society) (**B**) Competitive adsorption of Ag(I) and Cu(II) by Ag(I)-imprinted CS hydrogels with different imprinting ratios. (Reproduced with permission from Ref. [170]. Copyright 2012 American Chemical Society) (**C**) The effect of pH on Ag(I) and Cu(II) ions sorption by Ag(I)-imprinted CS gel beads. (Reproduced with permission from Ref. [171]. Copyright 2015 Elsevier) (**D**) Ag(I) sorption isotherms (with Langmuir and Freundlich models fitting profiles) by Ag(I)-imprinted and non-imprinted CS-P(DVB-glycidyl methacrylate (GMA)-ST) particles. (Reproduced with permission from Ref. [172]. Copyright 2015 American Chemical Society) (**E**) Selectivity of Ag(I) sorption by Ag(I)-imprinted and non-imprinted CS-P(DVB-GMA-ST) particles. (Reproduced with permission from Ref. [172]. Copyright 2015 American Chemical Society) (**F**) The effect of morphology on Ag(I) sorption by Ag(I)-imprinted CS-P(DVB-GMA-ST) particles. (Reproduced with permission from Ref. [172]. Copyright 2015 American Chemical Society).

Song et al., investigated the influence of Ag(I)-imprinted mass ratios on the sorption and selectivity performance of CS hydrogels prepared by the phase inversion method [170]. An increase of about 40% in Ag(I) sorption performance was found for the imprinted hydrogels, irrespective of the CS to Ag(I) mass ratio, compared to the non-imprinted hydrogel (Figure 17A). However, the imprinting ratio significantly influenced the selectivity of Ag(I) sorption over Cu(II) (Figure 17B). Thus, while the non-imprinted CS hydrogel preferentially adsorbed Cu(II) over Ag(I) ions, the affinity toward the two metal ions shifted with the increase in the Ag(I) mass ratio in the imprinted hydrogels. This was attributed to the templates with favorable geometry formed in the CS hydrogels during the imprinting process, which promoted the selective recognition of Ag(I) ions. Additionally, the adsorbed Ag(I) ions could be easily eluted with 0.5 M HNO_3_ solutions, and the sorbents preserved their sorption capacity for up to five sorption–desorption cycles.

Ag(I)-imprinted CS beads were prepared by Zhang et al., by GA cross-linking as potential selective sorbents of Ag(I) ions from wastewaters [171]. The authors reported a bimodal Ag(I) sorption profile as a function of pH and a very high selectivity toward Ag(I) ions in a bicomponent solution with Cu(II) (Figure 17C). The maximum Ag(I) ions sorption capacity was 89.2 mg/g, at 298 K and a sorbent dose of 1 g/L (Table S9). Using FTIR and XPS, the authors established that the sorption of Ag(I) ions proceeded by ion exchange from pH 1 to pH 3, and by complexation from pH 3 to pH 5.

The particles’ morphology is known to influence the HMIs’ sorption performance because it affects the availability of functional groups toward sorbate species. To investigate this issue, CS-modified P(DVB-GMA-ST) particles were prepared by Hou et al., through the IIT as solid particles (Ag-IISPs), hollow particles (Ag-IIHPs), single-hole hollow particles (Ag-IISHPs), and Janus hollow particles (Ag-IIJHPs) [172]. In comparison to the non-imprinted particles, the imprinted ones exhibited a net superior sorption capacity of Ag(I) ions (Figure 17D). Among the different Ag(I)-imprinted particles, the highest Ag(I) sorption performance was recorded for the Ag-IISHPs ones, followed, in order, by Ag-IIHPs, Ag-IIJHPs, and Ag-IISPs (Figure 17D). All Ag(I)-imprinted particles were strongly selective toward Ag(I) sorption in mixtures containing Cu(II) and Zn(II) ions (Figure 17E), the selectivity coefficients being in the range 4.5–6.5. The high sorption performance of Ag-IISHP for Ag(I) ions was explained by the fact that both its internal and external surfaces were available to interact with the metal ions. However, in an oil–water mixture, the Ag-IIJHP exhibited the highest Ag(I) sorption performance, attributed to the amphiphilicity of the Janus structure (Figure 17F) [172].

Ag(I)-imprinted beads comprised of CS and TEA with very high sorption capacity for Ag(I) ions, tunable sorption kinetics, high selectivity, and reusability, were reported by Zhang et al. [173]. It was established that the beads preparation strategy significantly influenced the maximum Ag(I) sorbed amount, as well as the sorption kinetics. The CS/TEA hydrogel beads prepared by dropping a CS solution containing Ag(I) ions into a coagulation bath with TEA had a maximum Ag(I) sorption capacity of 510 mg/g at pH 5, the sorption equilibrium being established in less than 1 h (Table S9). On the other hand, by thermally treating (2.5 h at 60 °C) the above CS/TEA hydrogel beads, the maximum Ag(I) sorbed amount declined to only 350 mg/g, while the sorption equilibrium was established in a much longer time (≈10 h). This behavior was explained by the decline of bead porosity as a result of thermal treatment, which prevented/delayed the Ag(I) ions diffusion inside the sorbent. On the other hand, the thermal treatment resulted in a slight improvement in the beads’ selectivity toward Ag(I) (in mixtures with Cu(II), Pb(II), and Zn(II)), as well as in their stability in successive sorption–desorption cycles (no sorption performance decline in 20 cycles) [173].

#### 3.10.3. Gold-Imprinted Polymers

Unlike other HMIs, in acidic aqueous environments, Au(III) is found as anionic species, i.e., gold chloride complexes (AuCl_4_^−^). This allows its selective adsorption from complex multi-HMIs matrices by means of electrostatic attractions with the protonated ligands (such as –NH_3_^+^ or –OH_2_^+^) on the surface of IIPs.

A new IIP resin based on EDA/*N*-(2-(1-imidazolyl ethyl)/CS for the selective recovery of Au(III) ions from simulated mining, as well as from acid mining drainage wastewaters, was prepared by Ahamed et al. [174]. The maximum Au(III) adsorption capacity estimated from the Langmuir equation was 810.67 mg/g, at pH 3 and 1 g/L sorbent dose (Table S9). The sorption kinetics was best described by the PSO kinetic model, supporting chemisorption as a possible adsorption mechanism, while thermodynamic studies revealed that the Au(III) removal was spontaneous and endothermic. The Au(III) IIP resin was best regenerated with a 0.7 M thiourea (TU)–2 M HCl solution, the adsorption efficiency in the fifth sorption–desorption cycle being still 95%. Furthermore, the selective recovery of Au(III) from simulated mining solutions and from acid mining drainage wastewaters containing Pd(II), Cu(II), Fe(III), and Pt(IV) as competing HMIs was a solid support that the IIP resin could be successfully used to industrially recovery of Au(III) ions [174].

In another work, Firlak et al., prepared an Au(III)-imprinted hydrogel by the photopolymerization of 4-acryloylmorpholine, 2-hydroxyethyl acrylate, and PEGDA (cross-linking monomer) in presence of Au(III) ions [175]. The IIP hydrogel had a maximum Au(III) sorption capacity of 78.43 mg/g, at pH 1 and 3 h contact time. Relative selectivity factors of 50,378, 72,588, and 40,381 were reported for Au(III)/Fe(III), Au(III)/Hg(II), and Au(III)/Zn(II), respectively, from multicomponent wastewaters, indicating that the IIP hydrogel could be used for quantitative separation of Au(III). Moreover, it could be reused for up to five sorption–desorption cycles with 99% sorption capacity recovery.

The IIP technology has also been combined with the strong affinity of Au(III) ions for sulfur-based ligands to fabricate new bio-sorbents for the selective recovery of Au(III) ions from wastewaters [176,177].

Recently, Gao et al., reported on the preparation of TU-functionalized ALG beads with a remarkable selectivity for Au(III) recovery from multi-HMI solutions by combining the IIP technology with the direct templating method [176]. The IIP beads attained a maximum sorption capacity of 184.82 mg Au(III)/g at 323 K in single component solutions, the adsorption equilibrium being reached in 6 h. In multi-HMI solutions, the Au(III)-imprinted TU-functionalized ALG beads exhibited an exceptional selectivity for Au(III) compared to Cu(II), Co(II), Ni(II), and Pb(II). For example, Au(III)/M(II) separation factors of 883.21, 1895.94, 2985.37, and 7634.99 were determined at 0.05 M HCl, when M(II) was Cu(II), Co(II), Ni(II), and Pb(II), respectively (Table S9). Au(III) sorption by the imprinted beads was strongly influenced by the illumination conditions, being much higher under UV mercury light irradiation compared to no light or Xenon light irradiation. This was assigned to the photoreduction of Au(III) ions to Au nanoparticles (NPs) under UV mercury light conditions. SEM images confirmed the presence of Au NPs on the surface of TU-functionalized ALG beads after UV mercury light irradiation, while metallic NPs were not visible at no light or Xenon light irradiation. The high-resolution SEM micrographs showed that the Au NPs on the surface of Au(III)-imprinted ALG beads were spherical, triangular, and hexagonal. Using XPS, it was ascertained that the oxygen, nitrogen, and sulfur functional groups within the TU-functionalized ALG beads were involved in the adsorption of Au(III), while the hydroxyl groups contributed to the Au(III) photoreduction [176].

Monier et al., first grafted 2-mercaptobenzaldehyde onto CS, then the new polymer interacted with Au(III) ions, cross-linked with ECH, before leaching the template ions, to produce a new Au(III)-IIP sorbent [177]. A maximum sorption capacity of 370 mg Au(III)/g was reported at pH 4 and 1 g/L sorbent dose. The Au(III) adsorption occurred mainly by ion-exchange at pH < 3, while at pH > 4, Au(III) ions were mainly sorbed by coordination with the thiol functional groups. The *k’* values for Au(III) recovery from mixtures containing Cu(II), Ni(II), Cr(III), and Pd(II), at pH 4, were 4.51, 3.57, 4.47, and 5.13, respectively (Table S9). Furthermore, the thiol-functionalized CS sorbent was successfully used in seven sorption–desorption cycles, with a 95% sorption performance recovery in the last cycle, by applying a desorption procedure involving its treatment with 0.5 M thiourea solution acidified with 1 M H_2_SO_4_ [177].

#### 3.10.4. Palladium-Imprinted Polymers

**Figure 18:**
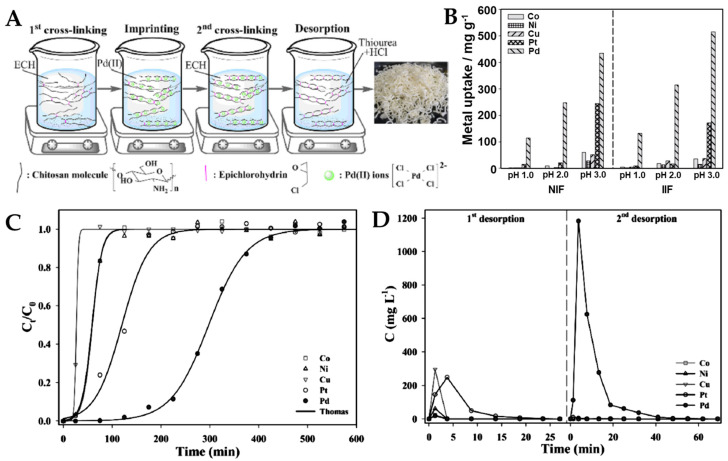
(**A**) Illustration with the Pd(II)-imprinted CS fibers by double ECH cross-linking. (Reproduced with permission from Ref. [178]). (**B**) The effect of equilibrium pH on the sorption of metal ions by non-imprinted (NIF) and Pd(II)-imprinted (IIF) CS fibers. (Reproduced with permission from Ref. [179]. Copyright 2015 Elsevier) Column adsorption (**C**) and desorption (**D**) of Pd(II), Co(II), Ni(II), Cu(II), and Pt(IV) from hydrometallurgy wastewater onto the Pd(II)-imprinted CS fibers. (Reproduced with permission from Ref. [180]. Copyright 2020 Elsevier).

Because of its high market demand and limited supply sources, attention is nowadays pointed toward Pd(II) recovery from secondary sources, such as waste electrical and electronic equipment (WEEE) [178]. To do this, the metallic components of WEEE are usually dissolved in strongly acidic solutions, in hydrometallurgical processes. Thus, Pd(II) is transformed into PdCl_4_^2−^, but at the same time, different other HMIs are dissolved, which complicates the selective recovery of Pd(II). To address this issue, Pd(II)-imprinted CS fibers were prepared by double ECH cross-linking (Figure 18A) and tested for the selective recovery of Pd(II) ions from highly acidic or from hydrometallurgy wastewaters, by batch or in packed columns experiments [178,179,180].

A maximum sorption capacity of 326.4 mg Pd(II)/g estimated by Langmuir model fitting was reported by Lin et al., at pH 1, 298 K, and 0.4 g/L sorbent dose for the Pd(II)-imprinted CS fibers in presence of Pt(IV) as a cross-contaminant (Table S9) [179]. The Pd(II)/Pt(IV) selectivity coefficients varied as a function of sorption pH, being 2082, 29.12, and 5.48 at pH 1, pH 2, and pH 3, respectively. Moreover, the imprinted CS fibers were capable of selectively adsorbing Pd(II) ions from a multi-HMIs solution containing Pt(IV), Co(II), Ni(II), and Cu(II) (Figure 18B). The mechanism of Pd(II) sorption established by FTIR and XPS indicated the electrostatic interaction between the protonated amino groups of CS fibers and Pd(II) ions (present as PdCl_4_^2−^) at strongly acidic pH, while the hydroxyl groups did not contribute to Pd(II) adsorption. A slight increase in Cu(II), Ni(II), and Co(II) sorbed amounts by the CS fibers was noted as the pH increased from 1 to 3, which was correlated with the steady deprotonation of amino groups. The selectivity of imprinted CS fibers toward Pd(II) ions could be optimized with respect to several preparation conditions, including ECH dosage in the first and second cross-linking steps and the pH of the imprinting reaction medium [179]. Furthermore, they have successfully been used in the recovery of Pd(II) ions from extremely acid hydrometallurgy wastewater (pH 0.2) in a packed column setup (flow rate: 0.25 mL/min) containing also Pt(IV), Co(II), Ni(II), and Cu(II) ions (Figure 18C) [180]. The column showed poor adsorption performance for Co(II), Ni(II), Cu(II), and Pt(IV) ions, while the exhaustion with Pd(II) ions was reached after 448 min of operation. A sorption capacity in dynamic conditions of 94.2 mg Pd(II)/g was estimated by fitting the experimental data with the Thomas model. Furthermore, the column was successfully regenerated by a two-step procedure involving the treatment with 1 M NaOH and acidified thiourea solutions, which allowed the recovery of a nearly pure Pd(II)-enriched eluent (Figure 18D) [178].

Pd(II)-imprinted chelating resins based on 2-aminobenzaldehyde-functionalized CS capable to retain up to 275 mg Pd(II)/g at pH 5, 303 K, and a sorbent dose of 1 g/L were prepared by Monier et al. [181]. The equilibrium of Pd(II) sorption by the imprinted functionalized CS resin was reached in 1 h, with the sorption kinetics being best described by the PSO kinetic model. This supports chemisorption as the probable interaction mechanism between Pd(II) and the ligand groups on the resins. Using FTIR and XPS, the authors probed that, at pH 5, Pd(II) ions coordinated in square planar geometry at the amino groups originating from CS and 2-aminobenzaldehyde. The relative selectivity coefficients of the imprinted CS resin compared to the non-imprinted one for Pd(II) separation from multi-HMIs solutions containing Cu(II), Ni(II), Co(II), and Mn(II) were 1.97, 1.88, 2.05, and 2.47, respectively (Table S9). They were lower compared to the CS fibers prepared by Lin et al. [179], this being explained by the fact that at pH 5 the competing HMIs could also coordinate with the functional groups of the CS resin. The 2-aminobenzaldehyde-functionalized CS resin was regenerated with 1 M TU aqueous solution acidified with 1 M HCl and exhibited a 96% sorption performance recovery after five successive sorption–desorption cycles [181].

### 3.11. Ion-Imprinted Polymeric Materials for Selective Extraction of Radionuclides and Rare Earth Metal Ions (Ce, Dy, Gd, La, and Nd)

Various separation and preconcentration procedures such as chemical precipitation, ion-exchange, solvent extraction, and sorption on the solid phase have been established to separate radioactive ions and rare earth metal ions from nuclear wastewaters [35,182,183,184,185,186,187,188,189,190,191,192,193,194,195,196,197,198,199,200,201,202] (Table 5). At the present time, numerous polymeric adsorbents are increasingly used to remove toxic ions from wastewaters. These materials have different advantages such as the control of the adsorption capacity and good selectivity toward toxic ions from wastewaters.

Uranium appears in the environment as a result of leaching from natural deposits, release from mill tailings, emanations from the nuclear industry, combustion of coal and other fossil fuels, and by the release of radioactive wastewater from nuclear research. Uranium is a highly toxic radioactive metal ion that can cause progressive or irreversible renal damage, which, in acute situations, may lead to kidney failure and death. The maximum uranium concentration in drinking water recommended by the WHO [203] is 15 μg/L, and the maximum contaminant level set by the U.S. Environmental Protection Agency [203] for drinking water standard is 20 μg/L.

**Figure 19:**
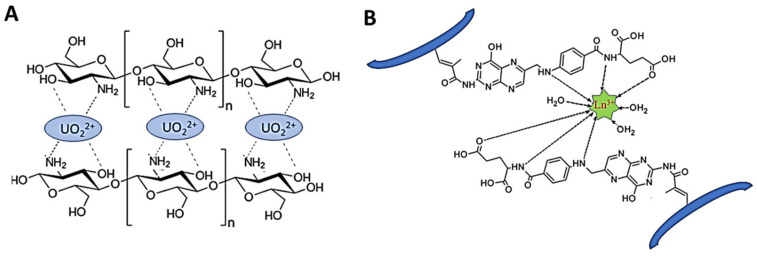
(**A**) Coordination of uranyl ions in active sites on the CS surface (adapted from Ref. [188]. Copyright 2020 Elsevier). (**B**) Chelation of lanthanides in cavities of IIP (adapted from Ref. [202]. Copyright 2015 American Chemical Society).

Liu et al. [187] synthesized ion-imprinting IPN hydrogels based on CS and PVA using as the cross-linker ethylene glycol diglycidylether and uranyl ions as the template, which have a maximum adsorption capacity of 156 mg/g for uranium ions in the pH range 5–6. By combining two procedures, freeze-drying and ion-imprinting, Dai et al. [188] obtained a microporous ion-imprinted CS foam that has a higher adsorption capacity (248.9 and 253.6 mg/g) compared with NIP CS foam (179.6 mg/g) (Figure 19A).

Starting from pure CS, Elsayed et al. [189] synthesized an amidoxime-modified CS by the introduction of cyanoacetic acid followed by an amidoximation with hydroxylamine for the selective retention of uranyl ions from wastewaters. This new IIP has a high adsorption capacity for uranyl ions of 332 mg/g. By incorporating CS with kaolin clay, Yu and co-workers [190] synthesized a new IIP composite that can be used for the selective removal of uranyl ions from radioactive wastewaters with a maximum adsorption capacity of 286.85 mg/g. Monier et al. [191] synthesized an ion-imprinted chelating resin based on modified CEL that demonstrated a maximum adsorption capacity toward uranyl ions of 180 mg/g. In all these cases, the optimum pH for the adsorption process was between 5 and 7, and the adsorption kinetics was found to be consistent with PSO kinetic model and the adsorption isotherms could be described by Langmuir model, indicating a chemisorption mechanism.

Thorium appears in the environment from the nuclear industry and from different anthropogenic activities. This radionuclide is stable in oxidation state +IV and is regularly used as an analog, from a chemical point of view, of unstable tetravalent radionuclides (Pu or Np). He et al. [192] prepared a magnetic Th(IV)-imprinted polymer based on a complex of *N*,*N′*-bis(3-allyl salicylidene)-o-phenylenediamine with Th(IV), as the functional monomer, by surface imprinting technology. This imprinted polymer has a high affinity, good efficiency (*q_e_* = 42.54 mg/g), and reusability. Huang et al. [193] used ECH as a cross-linking agent to obtain a magnetic ion-imprinted CS resin for the removal of Th(IV) from wastewater. The maximum adsorption capacity of this sorbent calculated from Langmuir isotherm was 147.1 mg/g in the acid medium; this process being well described by the PSO kinetic model.

Gore and co-workers [194] obtained a higher adsorption capacity (325 mg/g) of Th(IV) from synthetic radioactive waters by using ion-imprinted electrospun nanofibers of CS/1-butyl-3-methylimidazolium tetrafluoroborate. Kinetic studies point out that the IIP followed the PSO kinetic model, with the chemisorption being the rate-controlling step during the removal of pollutants. 

Lanthanide metals are very important in obtaining catalysts, ceramic materials, magnetic materials, and radar systems. Thus, their selective recovery has received great attention because is difficult to separate the lanthanides due to their chemical similarities. Moreover, the presence of lanthanides in residual waters is of environmental interest, and it is necessary to treat them prior to their release into the environment. More and more studies have involved the use of IIPs for separation, pre-concentration of lanthanides, and for the de-pollution of the wastewaters [195,196,197,198,199,200,201,202] (Table 5).

Zhang and co-workers [195] obtained a chitosan-carbon imprinted aerogel by using CS, acidified MWCNTs, polydopamine, and GO for the separation of Gd(III) from an aqueous solution in static and dynamic systems. In order to highlight the selectivity of this sorbent toward Gd(III), multi-component systems were investigated in the presence of Dy(III), Nd(III), and Pr(III). The obtained results showed the higher affinity of the sorbent for the Gd(III). Similarly, Liu et al. [196] obtained a new imprinted carbon nanotubes-CS hybrid sponge with a tridimensional structure by combining polymerization and lyophilization techniques for the adsorption of Gd(III). This sorbent preferentially adsorbs 88.9% Gd(III) within 1 h and has a rapid regeneration. The preference of the sorbent for the Gd(III) is due to the spatial structure of the cavities that match only with Gd(III) geometry.

Pan and co-workers [197] applied an advanced surface-imprinting technique in order to obtain Ce(III) IIP as hollow microspheres that have greater surface area and pore volume. Due to these characteristics, Ce(III) IIP demonstrated a high adsorption capacity (130.23 mg/g) for Ce(III) and high selectivity in presence of La(III), Fe(III), Pb(II), and Sr(II). In another study, reported by Keçili and collab. [198], Ce(III) was efficiently removed in a dynamic system by an IIP cryogel based on *N*-methacryloamido-antipyrine, which demonstrated a selective maximum adsorption capacity of 36.58 mg/g.

Ali et al. [199] synthesized CS/polyvinylpyrolidone IIPs for the separation of La(III), Ce(III), and Sm(III) from aqueous solutions in batch and dynamic systems. Competitive adsorption experiments showed that La(III), Ce(III), and Sm(III) were adsorbed prior to the other interfering ions (Ho(III), Eu(III), Nd(III), and Co(II)). Ni et al. [200] chose vinylphosphonic acid (VPA) as the functional monomer in order to obtain an IIP that could be used for La(III) removal. This sorbent demonstrated a high adsorption capacity (62.8 mg/g), a high degree of reusability (about 100% after 5 regeneration cycles), and good selectivity for La(III) in the presence of interfering ions. Dy(III) ion-imprinted tridimensional macroporous CS membranes were prepared by an immersion–precipitation–evaporation technique and was successfully used for the selective adsorption of Dy(III) [201].

In most cases, the sorption process followed the PSO kinetic model, which indicates a chemisorption process, and was better fitted to the Langmuir adsorption isotherm, assuming monolayer adsorption onto sorbent surface with a restricted number of identical sites. Chemisorption supposed a chelation process between O and N-containing functional groups and Ln(III) (Figure 19B), involving the imprinting cavities that have a special spatial structure that is only complementary with Ln(III) geometry, which achieves the selective adsorption for these ions [202].

### 3.12. Other Group of Metal Ions-Imprinted Polymers (Ga, In, and Pb)

The main sorption performance data for the removal of Ga(III), In(III), and Pb(II) ions from wastewaters by different IIP materials are presented in Table S10.

Ga(III) is a valuable and scarce HMI used industrially as a raw material in the development of optical glass and semiconductors. Ga(III) recovery from wastewaters is performed by several methods (adsorption, ion exchange, solid-phase extraction, etc.), but often the selectivity of its recovery is very poor. To address this shortcoming, Wang et al., recently developed a new Ga(III)-imprinted bio-adsorbent based on AA-functionalized CS (denoted as Ga(III)-AA-CS) [204]. The prepared sorbent achieved a maximum sorption capacity of 192.4 mg Ga(III)/g at pH 3 and 303 K (Table S10). It also showed very good selectivity toward Ga(III) sorption in binary systems containing Si(IV), Ge(III), Al(III), or Zn(II) as competing ions, with selectivity coefficients of 3.64, 3.15, 2.19, and 1.3, respectively. The sorption mechanism, established by FTIR and XPS, was the cation exchange between Ga(OH)_2_^+^ or Ga(OH)_2_^+^ species and protonated amino groups (–NH_3_^+^) within the sorbent imprinted cavities. Moreover, the imprinted sorbent was successfully reused in six successive sorption–desorption cycles, with only an ≈10% decline in Ga(III) sorption capacity [204].

In(III) is also a naturally scarce element that is intensively used in many modern applications, including the development of liquid crystal displays and solar cells. Since no mineral that contains In(III) as its main constituent has been identified so far, its selective recovery from natural or industrial wastewaters is in high demand. In view of this, Li et al., fabricated In(III)-imprinted polymer composites by the surface imprinting technique, through the free-radical polymerization of VPA [205], or VPA and allyl mercaptan (AMT) [206] in the presence of *N*-propylmaleamic acid-functionalized silica gel. The prepared composites exhibited very good performance for the selective removal of In(III) from monocomponent solutions and complex pollutants mixtures, in batch, as well as in column studies. In batch experiments, maximum In(III) sorbed amounts of 45.07 mg/g [205] and 60.62 mg/g [206] were determined for poly(vilnylphosphonic acid) (PVPA)-*g*-SiO_2_ gel and P(VPA-AMT)-*g*-SiO_2_ gel composites, respectively. The optimum sorption pH for both resins was three, the equilibrium being established in less than 30 min. In column experiments, the In(III) sorption capacities for the two composites were 34.57 mg/g and 48.75 mg/g, respectively. Both composites exhibited very good selectivity for In(III) recovery from multi-HMIs wastewaters. Selectivity coefficients of 189.17, 67.94, 886.63, and 2479.71 were determined for In(III) separation from mixtures containing Cu(II), Pb(II), Zn(II), and Fe(II), respectively, by the (PVPA)-*g*-SiO_2_ gel composite (Table S10) [205]. On the other hand, the selectivity coefficients for the same HMIs in the case of P(VPA-AMT)-*g*-SiO_2_ gel composite were 40.74, 211.86, 36.91, and 69.44 (Table S10) [206]. The noted differences in selectivity could be ascribed to the functional groups of each composite: only phosphate groups in the (PVPA)-*g*-silica gel composite, and phosphate and sulfhydryl groups in the P(VPA-AMT)-*g*-SiO_2_ gel one. Both composites exhibited excellent sorption performance recovery in a column in six sorption–desorption cycles, as well as in the recovery of In(III) from real mining wastewaters.

Pb(II) is one of the most toxic elements in nature. It is widely used in plating, paint, or battery industries, albeit its discharge into soil and water can cause severe health effects on humans, including high blood pressure, neurologic and fertility damage, or renal system failure [207]. Lately, the IIP technology has been used to produce many efficient materials for Pb(II) sensing [208,209] or removal [207,210,211,212,213,214,215,216] from wastewaters. For example, different Pb(II)-imprinted CS-based adsorbents functionalized with different organic ligands such as dithiocarbamate [210], tetraethylenepentamine (TEPA) [211], glutaric acid [216], or PAA [212] were prepared for the selective recovery of Pb(II) from wastewaters. A maximum sorption capacity of 359.68 mg Pb(II)/g and fast sorption kinetics (1.5 h) were reported by Liu et al., for dithiocarbamate-CS beads at pH 6 and 303 K [210]. The effect of the imprinting process on the recovery of Pb(II) ions from multi-HMIs solutions containing Cd(II), Cu(II), Ca(II), Mg(II), or Zn(II) was illustrated by the relative selectivity coefficients, which were 5.08, 2.45, 3.25, 4.24, and 3.20, respectively (Table S10) [210]. On the other hand, when CS beads were functionalized with TEPA ligands, the relative selectivity coefficients for Pb(II) recovery from mixtures with Cu(II) and Zn(II) increased up to 5.08 and 15.41, but decreased for mixtures containing Cd(II), Ca(II), and Mg(II) ions (i.e., 2.17, 2.18, and 2.29, respectively) [211]. These differences could arise not only from the cavities obtained through the Pb(II) imprinting process, but also from the different functional groups involved in the complexation with Pb(II) ions: −NH_2_, −SH, and −C=S− for the dithiocarbamate-functionalized CS beads, and −NH_2_, −NH−, and −OH for the TEPA-functionalized ones. Both types of CS beads were successfully tested up to 10 sorption–desorption cycles, with good sorption recovery performance.

**Figure 20:**
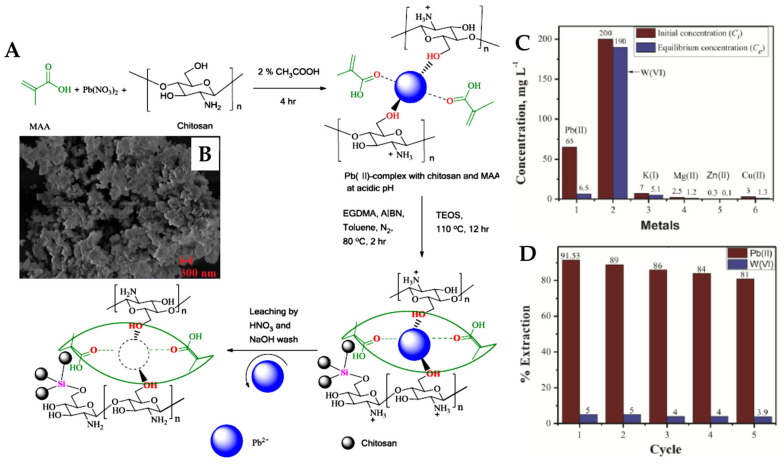
(**A**) Preparation of P(MAA-EGDMA)-*g*-CS IPN hydrogels in presence of Pb(II) ions as template. (**B**) SEM micrograph of the Pb(II)-imprinted P(MAA-EGDMA)-*g*-CS IPN hydrogels. Sorption of Pb(II) ions in presence of competitive HMIs from printed circuit board recycling unit wastewaters (**C**) and sorption of Pb(II) and W(VI) in five consecutive cycles (**D**) in column setup (pH 6, flow rate 2 mL/min, column: height 7 cm, diameter 8 mm). (Adapted with permission from Ref. [207]. Copyright 2016 American Chemical Society).

A high selectivity for Pb(II) recovery in column experiments from printed circuit board recycling unit wastewaters was reported by Hande et al., for P(MAA-EGDMA)-*g*-CS interpenetrated network (IPN) hydrogels (Figure 20A) [207]. The porous IPN hydrogels (Figure 20B) were prepared by the cross-linking polymerization of MAA in presence of CS and of Pb(II) ions as the template and toluene as porogen, using EGDMA and TEOS to cross-link MAA and CS, respectively.

After leaching of Pb(II) ions (by treating with HNO_3_ and washing with NaOH), the obtained IPN hydrogels contained template cavities that were capable of selectively recognizing Pb(II) ions from multi-HMIs wastewaters also containing W(VI), K(I), Mg(II), Zn(II), Mn(II), and Cu(II) ions. The determined selectivity coefficients were 161.58, 77.58, 44.98, 31.00, 52.75, and 39.12, respectively (Table S10). The very high selectivity of the IPN hydrogels toward Pb(II) compared to W(VI) ions was attributed to the formation of tetra-coordinative recognition sites during the imprinting process, while W(VI) typically forms hexa-coordinative complexes. Furthermore, the imprinted P(MAA-EGDMA)-*g*-CS IPN hydrogel was successfully used for the selective recovery of Pb(II) from printed circuit board recycling unit wastewaters during column experiments, with 91.5%, 5%, 27.1%, 40%, 66.66%, and 56.66% removal performance toward Pb(II), W(VI), K(I), Mg(II), Zn(II), and Cu(II) ions, respectively (Figure 20C). The reusability studies using binary Pb(II) and W(VI) solutions showed that the selectivity for Pb(II) ions is preserved even after five sorption–desorption cycles (Figure 20D) [207].

In another work, He et al., prepared a magnetic Pb(II)-imprinted Fe_3_O_4_-CS biosorbent for the selective removal of Pb(II) ions from wastewaters [213]. Thus, the prepared biosorbent showed a maximum sorption capacity of 69.48 mg Pb(II)/g, at pH 5 and 8 h contact time. The *k’* values for the separation of Pb(II)/Cu(II), Pb(II)/Cd(II), and Pb(II)/Ni(II) mixtures by the Pb(II)-imprinted biosorbent were 2.32, 2.20, and 2.05 times higher, respectively, than for the non-imprinted composite. Furthermore, because of the magnetic properties of Fe_3_O_4_ nanoparticles, the biosorbent was easily separated, regenerated, and reused in five sorption–desorption cycles, with 90% sorption capacity recovery [213].

## 4. Conclusions and Perspectives

This review of IIPs represents an updated overview of the selective preconcentration and removal of almost all transition metal ions. The affinity and efficacy of IIPs toward template ions were discussed in terms of operational sorption parameters, such as pH, contact time, initial metal ion concentration, selectivity coefficients, and sustainability in successive sorption–desorption cycles.

Materials prepared by combining the ion-imprinting technology with other synthetic strategies (such as magnetic separation technology, membrane technology, cryogelation, freeze-drying, IPN, etc.) exhibited the best sorption performances in the selective solid-phase extraction of transition metal ions. An increasing trend consists of the synthesis of IIPs as sensors using electrochemical detection for hazardous metal ions.

Even though great advances have been accomplished in this growing field, there are enough places for many other improvements including:

- The majority of analyzed papers reported investigations performed on ideal lab-based aqueous solutions in batch experiments, and only a few of them covered the IIPs performance in fixed-bed column setups and real water samples. Expanding the understanding of IIPs’ sorption behavior in such conditions is mandatory toward their successful implementation in large-scale industrial applications.
- The current scenario to increase the IIPs’ economic benefits is to use them in multiple sorption–desorption cycles. In this context, the identification of alternative eco-friendly regeneration approaches, reduction in generated waste, and minimization of operation costs, while maximizing IIPs’ lifespan, are priority research directions that should be extensively addressed in the future.
- In addition, the focus should also be directed to the development of stimuli-responsive imprinted polymers as a new generation of intelligent selective sorbents. Moreover, the application and development of combined approaches are expected to grow considerably in the following years.

## Figures and Tables

**Table 1:** Equations of the models commonly used for the theoretical analysis of the sorption data.

Equation Number	Equation ^1^	Definition
Kinetic models		
(3)	$q_{t}=q_{e}\left(1-exp^{-k_{1}t}\right)$	Pseudo-first order (PFO) model
(4)	$q_{t}=\frac{q_{e}^{2}k_{2}t}{1+q_{e}k_{2}t}$	Pseudo-second order (PSO) model
(5)	$q_{t}=k_{id}t^{0.5}+C$	Intraparticle diffusion (IPD) model
Isotherm models		
(6)	$q_{e}=\frac{q_{m}K_{L}C_{e}}{1+K_{L}C_{e}}$	Langmuir model
(7)	$q_{e}=K_{F}C_{e}^{N}$	Freundlich model
(8)	$q_{e}=q_{DR}e^{-K_{DR}\varepsilon^{2}}$	Dubinin–Radushkevich (D–R) model
(9)	$q_{e}=\frac{q_{m}a_{S}C_{e}^{N}}{1+a_{S}C_{e}^{N}}$	Sips model
(10)	$lnK^{o}=\frac{\Delta S^{o}}{R}-\frac{\Delta H^{o}}{RT}$	Van’t Hoff equation
(11)	$K^{o}=K_{D}\times M_{adsorbate}\times 55.5$	Standard equilibrium constant
(12)	$\Delta G^{o}=-RTlnK^{o}$	Standard Gibbs free energy of sorption, kJ mol^−1^

^1^*q_e_* and *q_t_*—amount of metal ion sorbed at equilibrium and at time *t*, min, respectively; *k*_1_—rate constant of the PFO kinetic model, min^−1^; *k*_2_—rate constant of the PSO kinetic model, g/mg × min; *k_id_*—intraparticle diffusion rate constant, mg/g × min^1/2^; *C*—constant describing the effect of boundary layer thickness, mg/g; *q_m_*—maximum theoretical sorption capacity, mg/g; *K_L_*—Langmuir constant, L/mg; *K_F_*—Freundlich constant, mg/g × mg^N^ × L^N^; *N*—measure of both the nature and strength of adsorption process, and of active sites distribution, related to the surface heterogeneity; the larger is its value, the more heterogeneous the sorbent system is; *q_DR_*—maximum sorption capacity of the metal ion, mg/g; *K_DR_*—D–R isotherm constant, mol^2^/kJ^2^; ε—Polanyi potential; *a_S_*—Sips constant; Δ*S°*—entropy, kJ/mol × K; Δ*H°*—enthalpy, kJ/mol; *R*—the gas constant, J/mol × K; *T*—the temperature in Kelvin; *M_adsorbate_*—is the abbreviation of atomic mass of each metal ion.

**Table 2:** Selective extraction of Cu(II) ions by IIP materials.

Polymeric Material	Functional Groups	pH	Contact Time, min	*q_m_*, mg/g	Interfering Ions	*k*	Remarks on Adsorption Process	Ref.
NH_2_-SiO_2_/PAAm hydrogels	–NH_2_	5	20	538	Pb(II) Cd(II) Ni(II)	5.58 6.05 5.89	Batch adsorption; Fitting: Langmuir/PSO; Reusability: 6 cycles (96.8% recovery).	[48]
P(HEMA-*co*-MAH) cryogel membranes	–NH_2_; –COOH; –OH	5.5	120	77.2	Cd(II) Pb(II) Zn(II)	3.7 11.1 2.08	Batch adsorption; Fitting: Langmuir/PSO; Reusability: 3 cycles (97.8% recovery).	[57]
Fe_3_O_4_/P(HEMA-*co*-MAH) cryogel membranes	–NH_2_; –COOH; –OH	5.5	120	179	Cd(II) Pb(II) Zn(II)	5.27 15.8 2.63	Batch adsorption; Fitting: Langmuir/PSO; Reusability: 3 cycles (98.7% recovery).	[57]
P(MAA-*co*-4-VP) beads	–COOH	6.2	180	14.92	Ni(II) Zn(II)	43.48 42.38	Batch/column adsorption; Fitting: Langmuir;	[52]
P(MMA-*co*-PA) beads	–COOH	5	60	19.2	Zn(II) Ni(II) Co(II);	57.7 26.8 34.0	Batch adsorption; Reusability: 7 cycles (97% recovery).	[56]
Fe_3_O_4_-CS microspheres	–NH_2_; –OH	5	480	109.89	Zn(II) Ni(II) Cd(II) Cr(VI)	38.866 7.103 31.679 2.297	Batch adsorption; Fitting: Langmuir/PSO; Reusability: 5 cycles.	[59]
PAAm/CS/zeolite cryogels	–NH_2_; –OH	4.5	20	260	Co(II) Ni(II) Zn(II) Pb(II)	7.28 24.42 47.02 34.85	Batch adsorption, Fitting: Langmuir/PSO; Reusability: 5 cycles.	[23,42]
CS/zeolite cryogels	–NH_2_; –OH	4.5	150	55.08	Zn(II) Ni(II) Fe(III) Cr(III)	4.1 4.61 1.84 11.33	Batch adsorption; Fitting: Dubinin–Radushkevich and Sips/PSO; Reusability: 5 cycles	[43]
CS/ATT gels	–NH_2_; –OH	5	60	35.20	Pb(II) Cd(II)	78.45 82.44	Batch adsorption; Fitting: Freundlich/PSO; Reusability: 10 cycles (86% recovery)	[60]
ALG/CS beads	–NH_2_; –COOH; –OH	5.7	480	83.33	Zn(II)	2.28	Batch adsorption; Fitting: Langmuir/PFO; Reusability: 3 cycles	[61]
Fe_3_O_4_-CS/GO composites	–NH_2_; –OH	6	120	132	Zn(II) Ni(II) Co(II) Cd(II)	45.44 85.04 86.9 29.83	Batch adsorption; Fitting: Freundlich/PSO; Reusability: 5 cycles.	[62]

Abbreviations: GO—graphene oxide; MAH—*N*-methacryloyl-L-histidine; PAAm—poly(acrylamide); ATT—attapulgite.

**Table 3:** Selective extraction of Cd(II) ions by IIPs materials.

Polymeric Material	Functional Groups	pH	Contact Time, min	*q_m_*, mg/g	Interfering Ions	*k*	*k’*	Remarks on Adsorption Process	Ref.
AAm-*g*-CS gels	–NH_2_; –OH	6	120	167	Ag(I) Cu(II) Ni(II) Zn(II)	4.56 4.13 4.11 4.20		Batch adsorption; Fitting: Langmuir/PSO; Applied in selective recovery of Cd(II) ions from Ni/Cd battery waste; Reusability: 8 cycles	[74]
CMCS-SiO_2_ composite	–NH-; –COOH; –OH	5	30	20.7	Pb(II) Co(II)		5.3 1.5	Batch/column adsorption; Fitting: PSO;	[76]
ATU/*N*-propylmaleamic acid-functionalized SiO_2_ composite	–NH_2_; –C=S; –OH	5	8	38.3	Ni(II) Cu(II) Co(II) Pb(II) Zn(II)	18.64 2.55 6.27 20.82 15.41		Batch/column adsorption; Reusability: 6 cycles; Tested for determination and preconcentration of Cd(II) ions from synthetic, tap, lake, and mine water samples	[77]
HMAM/DVE3 IPN hydrogel	–NH–; –OH	6	16	179.86	Cu(II) Ni(II) Pb(II)		8.33 8.79 9.18	Batch/column adsorption; Fitting: Freundlich/PSO; Reusability: 20 cycles (98.5% recovery)	[78]
MA-*co*-AN/DVB microspheres	–COOH; –C≡N	6		20.46	Cu(II) Mn(II) Ni(II) Pb(II)		15.2 4.10 9.20 3.01	Column adsorption; Tested for Cd(II) preconcentration and determination from tap, spring, and river water samples; Reusability: 10 cycles.	[79]
1-VI/MP/TRIM polymer resin	–N=; –SH; –OH	7.2	120	16.99	Pb(II) Zn(II) Hg(II) Cu(II) Ni(II) Ca(II) Mg(II) Na(I)	67.4 13.9 1.2 9.7 6.4 168.9 69.1 42.6		Batch adsorption; Fitting: Langmuir/PSO; Reusability: 4 cycles.	[80]
1-VI/AMP/TRIM polymer resin	–N=; –NH_2_; –OH	7.2	120	10.40	Pb(II) Zn(II) Hg(II) Cu(II) Ni(II) Ca(II) Mg(II) Na(I)	24.4 5.2 1.1 1.1 1.1 194.3 624.6 10.0		Batch adsorption; Fitting: Langmuir/PSO; Reusability: 4 cycles.	[80]

Abbreviations: AAm—acrylamide; CMCS—carboxymethyl CS; ATU—allyl thiourea; HMAM—*N*-hydroxymethyl acrylamide; DVB—divinylbenzene; DVE3—triethylene glycol divinyl ether; IPN—interpenetrating polymer networks; MA-*co*-AN—maleic acid-*co*-acrylonitrile; 1-VI—1-vinylimidazole; MP—(3-mercaptopropyl)trimethoxysilane; AMP—(3-aminopropyl)trimetoxysilane; TRIM—trimethylolpropane.

**Table 4:** Selective extraction of Ni(II) ions by IIPs.

Polymeric Material	Functional Groups	pH	Contact Time, min	*q_m_*, mg/g	Interfering Ions	*k*	Remarks on Adsorption Process	Ref.
CS films	–NH_2_; –OH	4	240	20	Zn(II) Cd(II) Co(II) Mg(II) Ca(II) Mn(II)	20.352 7.138 56.980 8.888 55.150 49.249	Batch adsorption; Fitting: Langmuir/PSO Selectivity coefficients: Reusability: 5 cycles	[101]
CS foam	–NH_2_; –OH	6	120	69.93	Co(II) Mn(II)	3.63 3.88	Batch adsorption; Fitting: Langmuir/PSO; Reusability: 5 cycles	[102]
Fe_3_O_4_-CS/PVA beads	–NH_2_; –OH	5.5	360	500	Cu(II) Ag(I) Zn(II)	15.05 23.06 18.25	Batch/Column adsorption; Fitting: Langmuir/PSO Reusability: 10 cycles	[103]
CS-AA/Fe_3_O_4_/MWCNTs	–NH_2_; –COOH; –OH	6	40	19.86	Pb(II) Cu(II)	13.09 4.42	Batch adsorption; Fitting: Freundlich/PSO; Reusability: 5 cycles	[104]
Melamine grafted CS/activated carbon biocomposite	–NH_2_; –N=; –OH	5	120	109.86	Zn(II) Cd(II) Cu(II) Pb(II)	3.13 4.48 3.72 2.51	Batch adsorption; Fitting: Langmuir/PFO; Reusability: 5 cycles	[105]
CMCS microspheres	–NH-; –COOH; –OH	6	360	82.78	Co(II) Mn(II) Cd(II)	5.64 2.68 2.06	Batch adsorption; Fitting: Langmuir/PFO; Reusability: 4 cycles	[106]
Fe_3_O_4_-CS nanoparticles	–NH_2_; –OH	7	60	18.5	Cu(II) Zn(II)	3.02 14.35	Batch adsorption; Fitting: Langmuir/PSO; Reusability: 15 cycles	[107]
PHEMA-MAH cryogels	–NH_2_; –COOH; –OH	6.5	60	5.54	Fe(III) Zn(II) Cu(II)	4.3 3.6 4.2	Batch adsorption; Fitting: Langmuir/Freundlich Reusability: 10 cycles	[108]
Fe_3_O_4_/bentonite/CoFe_2_O_4_/SiO_2_@ PVA nanocomposites	–OH	5.5	120	33.76	Cu(II) Zn(II) Cd(II)	3.768 2.507 2.149	Batch adsorption; Fitting: Langmuir/PSO; Reusability: 5 cycles	[109]
ALG beads	–COOH; –OH	7	1440	352.14	Cu(II) Co(II) Zn(II)	6.38 6.62 7.10	Batch adsorption; Fitting: Langmuir/Freundlich Reusability: 5 cycles	[110]
2-(Allylmercapto) nicotinic acid (ANA) composites	–N=; –S–; –COOH	6	20	38.85	Cd(II) Co(II) Cu(II) Mg(II) Zn(II)	16.89 4.23 32.74 219.59 33.63	Batch adsorption; Fitting: Langmuir/PSO; Reusability: 5 cycles	[111]
PMAA/diphenylcarbazide composites	–COOH; –NH–	7	30	86.3	Na(I) K(I) Mg(II) Ca(II) Ba(II) Al(III)	2.107 3.079 5.333 2.436 1.775 3.908	Batch adsorption; Fitting: Freundlich/PSO; Reusability: 5 cycles	[112]

Abbreviations: ALG—alginate; CS—chitosan; CMCS—carboxymethyl CS; PSO—pseudo-second order; Poly(hydroxyethyl methacrylate)—PHEMA; PMAA—poly(methacrylic acid); PVA—poly(vinyl alcohol); MAH—*N*-methacryloyl-L-histidine; MWCNTs—multi-walled carbon nanotubes.

**Table 5:** Selective extraction of radionuclides and rare earth metal ions.

Template Ions	Polymeric Material	Functional Groups	pH	Contact Time, min	q_m_, mg/g	Interfering Ions	k	Remarks on Adsorption Process	Ref.
U(VI)	CS/PVA cross-linked hydrogel	–NH_2_; –OH	5–6	120	156	Th(IV) Cu(II) Zn(II) Fe(III) Co(II) Ni(II) Mn(II)	6.11 5.05 9.52 7.07 11.51 7.50 9.71	Batch adsorption; Fitting: Langmuir/PSO	[187]
U(VI)	Amidoxime modified CS	–NH_2_; –OH	5	180	332	Th(IV) Al(III) Eu(III) Fe(III) Co(II) Ni(II) Cu(II) Pb(II)	9.47 16.78 12.91 11.14 17.73 17.24 10.55 21.97	Batch adsorption; Fitting: Langmuir/PSO; Reusability: 5 cycles	[189]
U(VI)	Honeycomb-like CS/kaoline clay	–NH_2_; –OH	5	120	286.85	Fe(III) Al(III) Mn(II) Co(II) Ni(II) Ca(II) Mg(II) Cu(II) Na(I) K(I)	7.2 10.66 12.87 14.04 18.10 25.41 45.34 4.91 84.73 64.17	Batch adsorption; Fitting: Langmuir/PSO; Reusability: 5 cycles	[190]
U(VI)	CMC-SAL	–OH; –N=	5	180	180	Fe(III) Mn(II) Co(II) Cu(II) V(V)	34.51 21.55 82.54 42.14 50.27	Batch adsorption; Fitting: Langmuir/PSO; Reusability: 5 cycles	[191]
Th(IV)	Salophen Schiff base magnetic IIP	–OH; –N=	4	30	42.54	La(III) Ce(III) Nd(III) U(VI)	649.2 595.8 96.6 71.1	Batch adsorption; Fitting: Langmuir/PSO; Reusability: 9 cycles	[192]
Gd(III)	MWCNTs-PDA-CS-GO	–NH_2_; –OH	7		150.86	Dy(III) Nd(III) Pr(III)	48.02 25.98 36.06	Batch adsorption; Fitting: Langmuir/PSO; Reusability: 5 cycles	[195]
Gd(III)	COOH-CNTs/CS-IIS sponge	–NH_2_; –COOH; –OH	7	720	71.95	Nd(III) Pr(III) Tb(III) Fe(III)	9.95 9.69 4.48 28.35	Batch adsorption; Fitting: Langmuir/PSO; Reusability: 5 cycles	[196]

Abbreviations: SAL—salicylaldehyde; PDA—polydopamine; CS—chitosan; PVA—poly(vinyl alcohol); PSO—pseudo-second order; CMC—carboxymethyl cellulose; MWCNTs—multi-walled carbon nanotubes; PDA—polydopamine; GO—graphene oxide; CNTs—carbon nanotubes.

## Data Availability

Not applicable.

## Supplementary Materials

The following supporting information can be downloaded at https://www.mdpi.com/article/10.3390/molecules28062798/s1, Table S1: Sorption of Zn(II) ions by IIPs. Table S2: Zn(II) Imprinted Polymeric Solid Phase Extractors. Table S3: Selective extraction of Co(II) ions. Table S4: Selective extraction of Fe(III). Table S5: Fe(III) Imprinted Polymeric Solid Phase Extractors. Table S6: Selective extraction of Hg(II). Table S7: Selective extraction of Cr(III) and Cr(VI). Table S8: Sorption of other transition metal ions by IIPs. Table S9: Selective sorption of Ag(I), Au(III), and Pd(II) ions by IIP materials. Table S10: Selective sorption of Ga(III), In(III), and Pb(II) ions by IIP materials.
