# Supplementary material for: Ion-Imprinted Polymeric Materials for Selective Adsorption of Heavy Metal Ions from Aqueous Solution
Source: Molecules. 2023 Mar 20;28(6):2798. doi: 10.3390/molecules28062798 (PMC10055817; doi:10.3390/molecules28062798)
Supplement: Supplementary file 1 [file molecules-28-02798-s001.zip › molecules-2283453-supplementary.pdf]

Supplementary Material

Ion-imprinted polymeric materials for selective adsorption of heavy metal ions from aqueous solution

Maria Marinela Lazar [1], Claudiu-Augustin Ghiorghita[1], Ecaterina Stela Dragan[1*], Doina Humelnicu[2], and Maria Valentina Dinu[1,*]

[1] Department of Functional Polymers, "Petru Poni" Institute of Macromolecular Chemistry, Grigore Ghica Voda Alley 41 A, 700487 Iasi, Romani

[2] Faculty of Chemistry, Alexandru Ioan Cuza University of Iasi, Carol I Bd. 11, 700506 Iasi, Romania

* Correspondence: sdragan@icmpp.ro (E.S.D.); vdinu@icmpp.ro (M.V.D)

Content:

Table S1: Sorption of Zn(II) ions by IIPs.

Table S2. Zn(II) Imprinted Polymeric Solid Phase Extractors.

Table S3. Selective extraction of Co(II) ions.

Table S4. Selective extraction of Fe(III).

Table S5. Fe(III) Imprinted Polymeric Solid Phase Extractors.

Table S6. Selective extraction of Hg(II).

Table S7. Selective extraction of Cr(III) and Cr(VI).

Table S8. Sorption of other transition metal ions by IIPs.

Table S9. Selective sorption of Ag(I), Au(III) and Pd(II) ions by IIP materials.

Table S10. Sorption of Ga(III), In(III) and Pb(II) ions by IIP materials.

The number of references is the same as in the main text.

Table S1. Sorption of Zn(II) ions by IIPs.

Imprinted HMI	Polymeric material	q_m, mg/g	Comments	Ref. Sec. 3.5.
Zn(II)	1-VI-HEMA-EGDMA	5.29	Batch adsorption<br>Conditions: pH 7.0; 298 K; 10 min<br>Fitted by Langmuir/PSO<br>Selectivity: k′ of ZnIIP was: 22.57, 5.44 and 46.17 for Zn(II)/Cu(II), Zn(II)/Ni(II) and Zn(II)/Co(II) ion pairs	[84]
	Morin ligand<br>MAA-*co*-EGDMA	45	Batch adsorption<br>Conditions: pH 6.0; 1.2 g L^{-1}; 3 min<br>Fitted by Langmuir<br>Selectivity: k′: 30.7, 23.9, 26.4, 42.6 and 26.8 with competitor ions Cu(II), Co(II), Ni(II), Pb(II) and Cd(II),	[85]
	2,2′-bipyridyl ligand<br>4-VP-*co*-EGDMA	13.77	Batch adsorption<br>Conditions: pH 6.2<br>Reusability: 10 % decrease of the original sorption capacity after 10 repeated cycles.<br>Selectivity coefficients for Zn(II)/Cu(II) and Zn(II)/Ni(II) were 8.52 and 52.	[90]
	Zincon ligand<br>GO/magnetic CS cross-linked with GA modified with AA<br>ST-EGDMA	71.4	Batch adsorption<br>Conditions: pH 8.5; 0.2 g L^{-1}; 298 K; 120 min<br>Fitted by Langmuir/PSO<br>Reusability: regenerated up to nine cycles without loss of sorption capacity<br>Determination of Zn(II) in various matrices.	[91]
	CS-MA cross-linked with EGDMA and TEOS	3.06	Batch adsorption<br>Conditions: pH 7.0; 2 g L^{-1}; 298 K; 120 min<br>Fitted by Langmuir/PSO	[92]
	CS/ATT/A-187	46.75	Batch adsorption<br>Conditions: pH 6.0; 1 g L^{-1}; 298 K; 360 min<br>Fitted by Langmuir/PSO	[93]

Abbreviations: AA - acrylic acid; ATT - attapulgite; A - 187-3-glycidoxypropyltrimethoxysilane; CS - chitosan; EGDMA - ethyleneglycol dimethacrylate; GO - graphene oxide; GA - glutaraldehyde; HEMA - 2-hydroxyethyl methacrylate; MAA - methacrylic acid; MA - methacrylamide; ST - styrene; TEOS - tetraethylortosilicate; 1-VI - 1-vinylimidazole; 4-VP - 4-vinylpyridine.

Table S2. Zn(II) Imprinted Polymeric Solid Phase Extractors.

Complexing Reagent	Zn(II) Imprinted Polymeric Solid Phase Extractors	Refs.
Salicylic acrylate	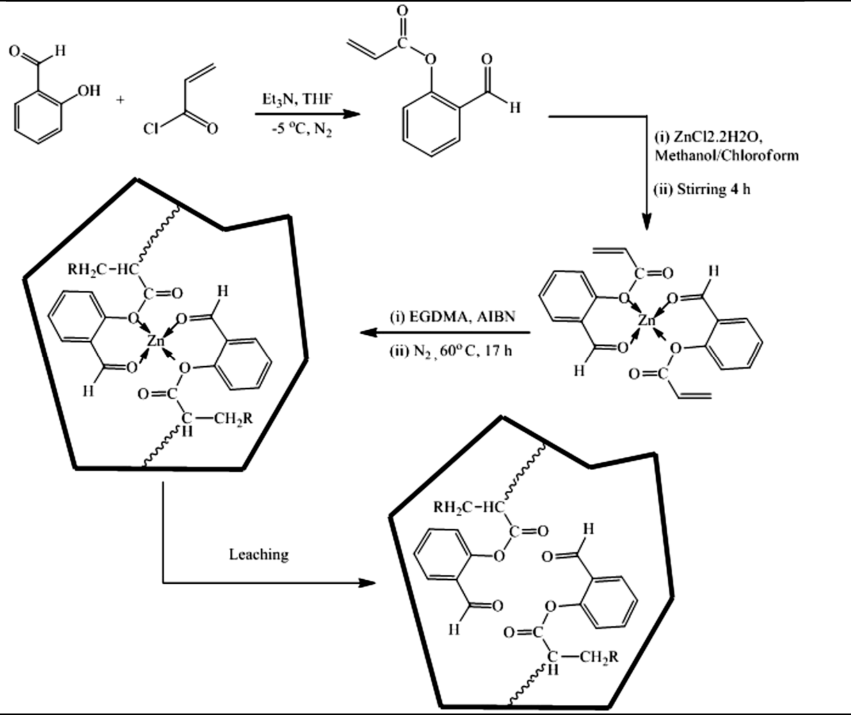	[87]
2,2′-Bipyridyl/4-VP	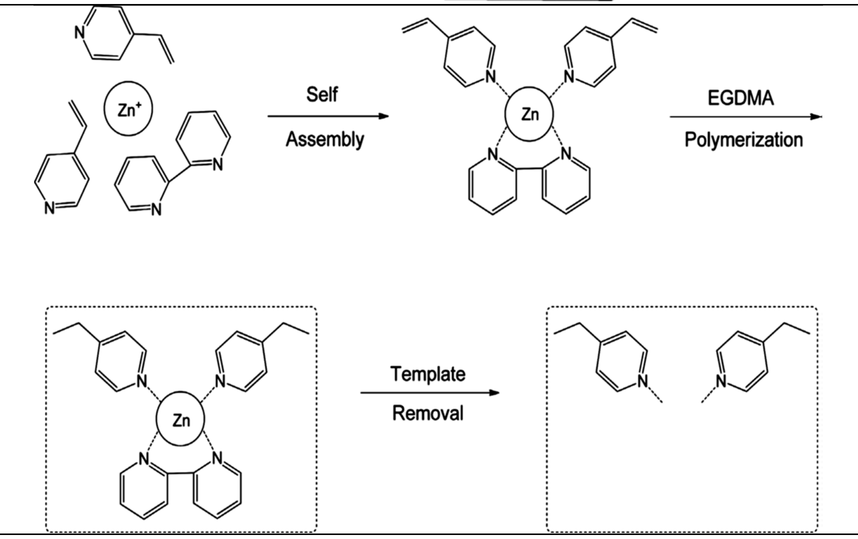	[90]

Zn(II) complexed with zincon-IIP grafted on GO/magnetic CS nanocomposite	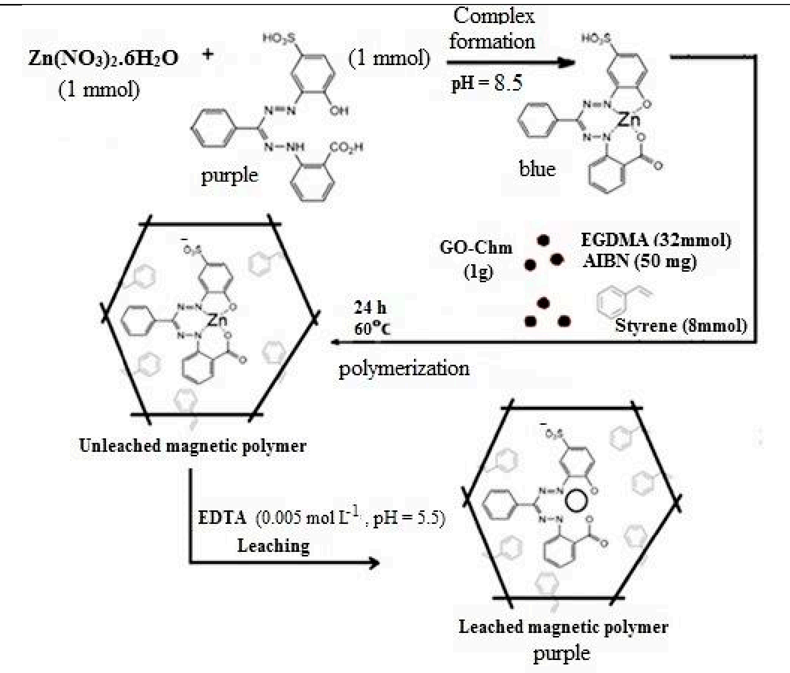	[91]

Table S3. Selective extraction of Co(II) ions.

Template ions	Polymeric material	pH	Contact time, min	q_m, mg/g	Interfering ions	k	Remarks on adsorption process	Ref.
Co(II)	P(MAA)/DPA composites		20	15.39	Ni(II)<br>Fe(III)<br>Mg(II)	18.46<br>13.77<br>30.11	Batch adsorption;<br>Reusability: 7 cycles	[95]
Co(II)	P(4-VP)/DPA composites		30	14.04	Ni(II)<br>Fe(III)<br>Mg(II)	9.39<br>38.53<br>10.41	Batch adsorption;<br>Reusability: 7 cycles	[95]
Co(II)	Fe_3O_4/SiO_2/P(1-VI) composites	7	180	23.09	Cu(II)<br>Cd(II)<br>Zn(II)<br>Pb(II)	19.99<br>50.28<br>11.02<br>7.56	Batch adsorption;<br>Fitting: Langmuir/PSO;<br>Reusability: 4 cycles	[96]
Co(II)	P(AAPTS)/Fe_3O_4@TiO_2@SiO_2 composites	8	30	35.21	Pb(II)<br>Ni(II)<br>Cd(II)	41.17<br>79.74<br>56.48	Batch adsorption;<br>Fitting: Langmuir;<br>Reusability: 7 cycles	[97]
Co(II)	P(B2MP-*co*-GG)/Fe_3O_4 composites	8	20	33.4	Fe(II)<br>Cu(II)<br>Mg(II)<br>Zn(II)<br>Ni(II)	5.25<br>4.05<br>6.06<br>11.81<br>4.48	Batch adsorption;<br>Fitting: Langmuir/PSO;<br>Reusability: 6 cycles	[98]

Co(II)	8-HQ/γ-Fe_2O_3-CS biocomposites	8	10	100	Cd(II)<br>Ni(II)<br>Pb(II)	11<br>42<br>2	Batch adsorption;<br>Fitting: Langmuir/PSO;<br>Reusability: 3 cycles	[99]
Co(II)	CS/zeolite composite cryo-beads	4	1440	120.4–126.6	Cu(II)<br>Ni(II)<br>Cd(II)<br>Fe(III)	9.543<br>6.660<br>20.320<br>225.391	Batch adsorption;<br>Fitting: Sips and Double Langmuir;<br>Reusability: 5 cycles	[100]
Co(II)	CS/zeolite composite cryo-beads	6	1440	86.2–102.3	Cu(II)<br>Ni(II)<br>Cd(II)<br>Fe(III)	1.589<br>1.087<br>2.025<br>14.692	Batch adsorption;<br>Fitting: Sips and Double Langmuir;<br>Reusability: 5 cycles	[100]

Abbreviations: PMMA – *N*-(pyrrolidin-2-ylmethyl) methacrylamide; AAPTS – 3-(2-aminoethylamino) propyltrimethoxysilane; B2MP – bis(2-methacryloxyethyl) phosphate; GG – glycylglycine; 8 - HQ–8-hydroxyquinoline; DPA – dipicolinic acid.

Table S4. Selective extraction of Fe(III).

**Template ions**	**Polymeric material**	**pH**	**Contact time, min**	q_m, **mg/g**	**Interfering ions**	***k***	***k′***	**Remarks on adsorption process**	**Ref.**
Fe(III)	HEMA+EGDMA +MAC- Fe^{3+}<br>($HEMAC-Fe^{3+}$)<br>(monolith)	5.0		0.92	Fe(II)<br>Cd(II)<br>Ni(II)		24.8<br>37<br>59.7	Column adsorption; flow rate 0.5 mL min-1<br>Fitted by Langmuir	[114]
Fe(III)	MAAP-Fe^{3+}-NIPAAm	4.0	30	14.23	Ni(II)<br>Co(II)<br>Mn(II)<br>Zn(II)<br>Al(III)<br>Cu(II)		83.4<br>66.4<br>62.5<br>41.2<br>16.3<br>14.8	Batch adsorption<br>Fitted by Langmuir	[115]
Fe(III)	(DHBPT)2-Fe(III)<br>+MAA+EGDMA	4.5	15	40.41	Mn(II)<br>Cd(II)<br>Cu(II)<br>Pb(II)<br>Zn(II)<br>Ni(II)<br>Al(III)<br>Cr(III)<br>Fe(II)		2.98<br>2.36<br>1.61<br>1.64<br>1.71<br>2.66<br>2.4<br>3.5<br>2.49	Batch adsorption<br>Reusability: sorption capacity loss of 3% at the end of the 6 sorption/desorption cycle	[116]
Fe(III)	2,2′-bipyridyl-Fe(II) complex	7.0	30	1.58	Mn(II)<br>Pb(II)		19.5<br>67.5	Batch adsorption<br>Fitted by Langmuir	[119]

	4-VP-TMPTMA								
Fe(III)	AA-Fe(III)<br>+<br>EGDMA	3.5		114.25	Cr(III)	492.18	50.87	Batch adsorption Fitted by Langmuir/PSO	[120]
Fe(III)	BMAOP-Fe(III)<br>+<br>EGDMA	2.0	180	15.71	Cr(III)<br>Co(II) Ni(II)<br>Cu(II)<br>Zn(II)	701<br>6926<br>1302<br>1171<br>2726	2.44<br>4.38<br>4.17<br>1.78<br>4.15	Batch adsorption Fitted by Freundlich/PSO Reusability: no obvious decrease of the sorption capacity after six cycles.	[121]
Fe(III)	Fe(III)-P(HEMA-MAGA)<br>+EGDMA	4.0	60	204.8	Cd(II)<br>Co(II)<br>Cr(III)<br>Ni(II)<br>Pb(II)<br>Zn(II)<br>Mn(II)		196.5<br>241.2<br>218.3<br>191.5<br>164.7<br>147.2<br>252.8	Batch adsorption	[123]
Fe(III)	Fe(III)-P(HEMA-MAGA)<br>+EGDMA	2.5	60	83.33	Zn(II)<br>Cr(III)<br>Al(III)		8.85<br>4.91<br>6.29	Batch adsorption Fitted by Langmuir/PSO Reusability: preservation of removal capacity up to 97.13% after ten cycles	[124]

Abbreviations: BMAOP – bis(2-methacryloxyethyl)phosphate; DHBPT - 3,6-bis (3,5-dimethyl-1-H-pyrzol-1-yl)-1,2-dihydro-1,2,4,5-tetrazine); MAAP - methacryloylaminoantipyrine; MAC - N-methacryloyl-(L)-cysteine methyl ester; MAGA - N-methacryloyl-(L)-glutamic acid; MMA - methyl methacrylate; NIPAAm - N-isopropyl acrylamide; TMPTMA - trimethylolpropane trimethacrylate.

Table S5. Fe(III) Imprinted Polymeric Solid Phase Extractors.

Complexing Reagent	Fe(III) Imprinted Polymeric Solid Phase Extractors	Refs.
Methacryloyl antipyrine	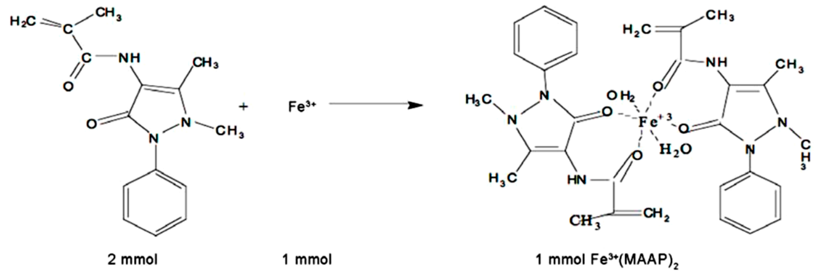	[115]
2,2′-bipyridyl	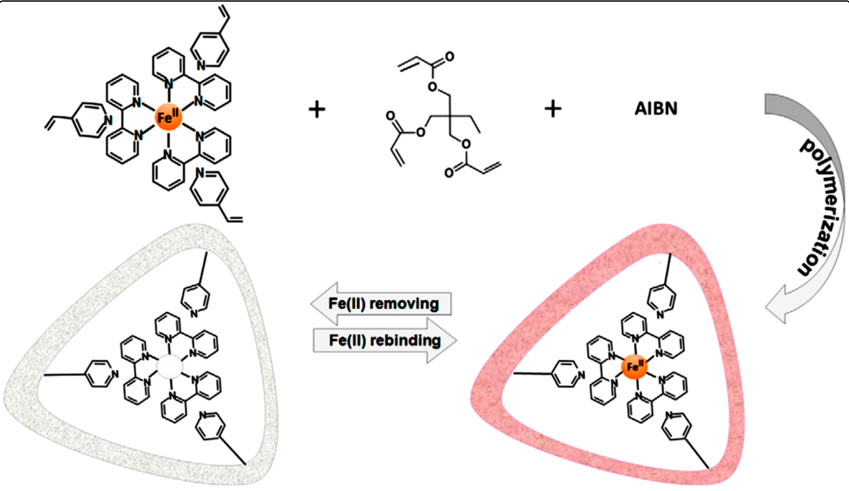	[119]
AA	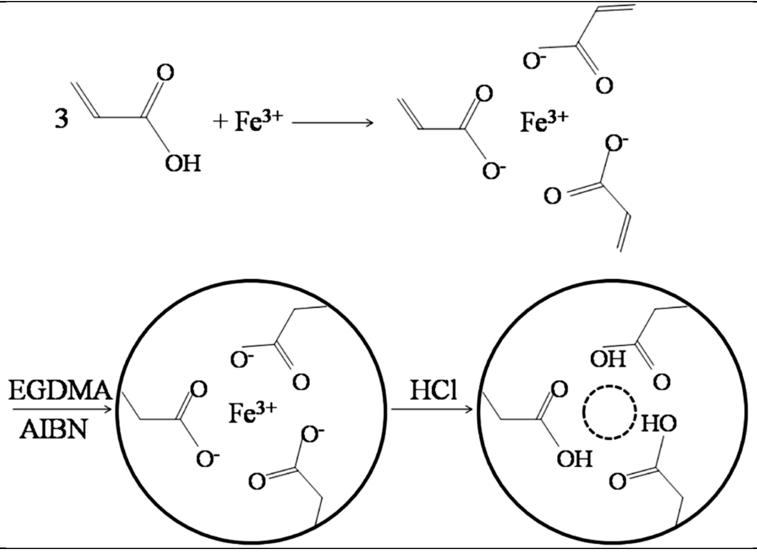	[120]

Bis(2-methacryloxyethyl) phosphate	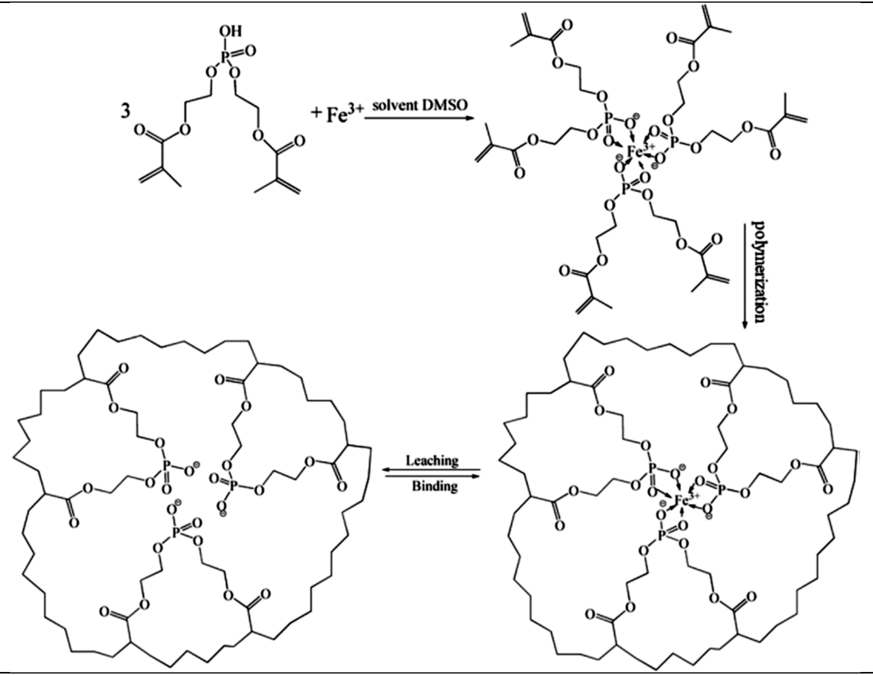	[121]

Table S6. Selective extraction of Hg(II).

Template ions	Polymeric material	pH	Contact time, min	q_m, mg/g	Interfering ions	*k*	*k′*	Remarks on adsorption process	Ref.
Hg(II)	Ligand-1-(2-thiazolylazo)-2-naphtol<br><br>MAA-TMPTMA microbeads	7.0	15	6.42	Cu(II)<br>Ni(II)<br>Co(II)<br>Pb(II)<br>Cd(II)<br>Zn(II)<br>$CH_3Hg(I)$	113.9<br>233.3<br>376.9<br>73.1<br>132.4<br>445.5<br>1633		Batch sorption;	[125]
Hg(II)	Ligand-1-pyrrolidinedithiocarboxylic acid<br><br>MAA-TMPTMA nanoparticles	7.0	30	12.84	Cd(II)<br>Cu(II)<br>Fe(III)<br>Mn(II)<br>Ni(II)<br>Pb(II)<br>Zn(II)<br>$CH_3Hg(I)$		143<br>450<br>400<br>695<br>776<br>330<br>860<br>1100	Batch sorption; Fitted by Langmuir/PSO Reusability: 5% lower sorption capacity after 100 cycles	[127]
Hg(II)	Dithizone/Hg(II) complex APTES-TEOSI	7.0	80	4.45	Zn(II)<br>Cd(II)<br>Pb(II)<br>MeHg(I)<br>EtHg(I)	33.54<br>26.63<br>22.09<br>19.06<br>21.56		Batch sorption; Fitted by Langmuir/PSO Reusability: 5% loss of the sorption capacity after ten sorption/desorption	[128]

								cycles	
Hg(II)	PB-CS	5.0	80	315	Pb(II)<br>Co(II)<br>Zn(II)<br>Cu(II)<br>Cd(II)		17,87<br>14.4<br>12.45<br>15.37<br>16.07	Batch sorption;<br>Fitted by Langmuir/PSO<br>Reusability: > 95% of the initial sorption capacity after five sorption/desorption cycles	[132]
MeHg(I)	MBT-MeHg(I) complex<br>+<br>AA-EGDMA	8.0		0.457	Hg(II)<br>Cd(II)<br>Pb(II)<br>Zn(II)		0.86<br>260<br>288<br>1510	Batch sorption;<br>Fitted by Freundlich/PSO	[136]

Abbreviations: APTES - 3-aminopropyltriethoxysilane; MBT - 2-mercaptobenzothiazole; PB - ligand Schiff base prepared from 4-amino-3-hydroxybenzoic acid and 2-pyridinecarboxaldehyde; RT - room temperature; TEOSI - triethoxysilane.

Table S7. Selective extraction of Cr(III) and Cr(VI).

Imprinted HMI	Polymeric material	q_m, mg/g	Comments	Ref.
Cr(III)	APTES as silane coupling agent; Cr(III)-IIP prepared by sol–gel reaction coupled with surface imprinting on the surface of mesoporous SBA-15.	38.5	Batch adsorption;<br>Conditions: pH 6.0; 298 K; 120 min.<br>Fitted by Langmuir/PSO;<br>Selective adsorption of Cr(III) in competition with: Co(II),<br>Cr(VI), Sr(II), Zn(II), Cu(II), Pb(II), Ni(II), in binary systems.	[137]
	Cr(III)-(phen)$_2$/(ST+EGDMA), acetonitrile as porogen		Dynamic adsorption;<br>Conditions: pH 4.5; 298 K; flow rate 1 mL min^{-1}<br>Reusability: 100 cycles<br>Selectivity coefficient in competition with Fe(III), Mn(II) and Cu(II): 9.6; 4.4; 16.7, respectively.	[139]
	Azo dye functionalized CS cross-linked with glyoxal	250	Batch adsorption;<br>Conditions: pH 5.0; 303 K; 1 g L^{-1}; 180 min.<br>Fitted by Langmuir/PSO;<br>Reusability: 96% of original capacity after fifth cycle<br>Selective adsorption of Cr(III) in competition with: Cu(II), Ni(II), Co(II),	[141]

			Fe(III), Eu(III), Al(III), in binary systems	
	ALG/PVA/ALG-AuNPs membrane	1.75	Batch adsorption;<br>Conditions: pH 6.0; 313 K; 20 h.<br>Fitted by Langmuir/PFO;<br>Reusability: four sorption/desorption cycles<br>Selectivity: high selectivity towards Cr(III) in presence of Cr(VI): sorption: > 97%/<5%	[142]
Cr(VI)	4-VP-DMAEMA-EGDMA	286.56	Batch adsorption;<br>Conditions: pH 2.0; 298 K; 1 g L^{-1}; 10 min.<br>Fitted by Langmuir/PSO;<br>Reusability: 5% decrease of the sorption capacity at the end of the fifth cycle<br>Selectivity constant in the presence of Cu(II), Cd(II) and Cr(III): 135.78; 145.44; 69.91	[143]
	Quaternary PP-*g*-DMAEMA fibers	156.5	Batch adsorption;<br>Conditions: pH 2.0; 298 K; 3 g L^{-1}; 240 min.<br>Fitted by Freundlich/PSO;<br>Reusability: small decrease of the original sorption capacity after five sorption/desorption cycles<br>Selectivity for $Cr_2O_7^{2-}$ in competition with HPO_4^{2-}, NO_3^-, SO_4^{2-}, with k′ of 36.7, 39.9 and 1.46, respectively.	[144]
	Pebax/CS/GO/APTES nanofibers	521.7	Batch adsorption;<br>Conditions: pH 3.0; 298 K; 0.5 g L^{-1}; 120 min.<br>Fitted by Redlich-Peterson/PSO;<br>Relative selectivity in respect with: Pb(II), Cu(II) and Ni(II) has been 11.53, 13.86 and 16.86, respectively.	[145]
	PP fibers-*g*-AA, amidated with TETA cross-linked with GA	167	Batch adsorption;<br>Conditions: pH 3.0; 303 K; 60 min.<br>Fitted by Freundlich/PSO;<br>Reusability: adsorption capacity towards Cr(VI) was > 82% after 10 sorption/desorption cycles	[148]
	Pre-complex (Cr(VI)+4-VP) + (Fe_3O_4/SiO_2) + (HEMA+EGDMA)	44.86	Batch adsorption;<br>Conditions: pH 3.0; 298 K; sorbent dose: 1.7 g L^{-1}; 20 min.<br>Fitted by Langmuir/PSO;	[149]

			Reusability: no loss of the sorption capacity after five cycles<br>Relative selectivity in respect with Ni(II), Cd(II), Cu(II), NO_3^-, SO_4^{2-} and PO_4^{3-}: 4.89, 3.29, 4.06, 2.95, 2.2; 2.45, respectively.	
	4-VP-*co*-EGDMA	3.28	Batch adsorption;<br>Conditions:pH 2.0; 3.2 g L^{-1}; 313 K; 30 min.<br>Fitted by Freundlich/PSO;<br>Reusability: good stability after 10 cycles	[151]
	1-VI-*co*-MAA-*co*-EGDMA	76.92	Batch adsorption;<br>Conditions: pH 2.0; 10 g L^{-1}; 323 K; 15 min.<br>Fitted by Langmuir/PSO;<br>Reusability: sorption capacity decreased with 1.87% at the end of tenth cycle.	[153]
	CMC/ECH/EDA	177.62	Batch adsorption;<br>Conditions: pH 2.0; 1 g L^{-1}; 298 K; 120 min.<br>Fitted by Langmuir/PSO;<br>Reusability: almost no decrease of the sorption capacity after the fifth cycle of adsorption/desorption	[154]

Abbreviations: DMAEMA - 2-(dimethylamino)ethyl methacrylate; CMC - carboxymethylcellulose; EDA - ethylene diamine; phen - 1,10-phenantroline; NPs – nanoparticles; PP - polypropylene; ALG - alginate.

Table S8. Sorption of other transition metal ions by IIPs.

Imprinted TMI	Polymeric material	q_m, mg/g	Comments	Ref.
Mn(II)	MAA-*co*-MBAA<br>On the surface of MWCNT	-	Batch adsorption<br>Conditions: pH 5.0; RT; 100 min<br>Fitted by Langmuir/PSO<br>Reusability: low decrease of the sorption capacity after five cycles.<br>Selectivity: the adsorption capacity decreased in the order: Mn(II) > Cr(III) > Fe(II) > Co(II); detection limit of Pt/MWCNT-IIP for Mn(II) was 0.0138 mM	[158]
Mo((VI)	Mo (VI)-ICTGBs	~300	Batch adsorption<br>Conditions: pH 6.0; 2 g L^{-1}; RT; 500 min<br>Fitted by Freundlich/PSO	[159]

			Reusability: reused up to 10 times with 300 mg g^{-1} > Q > 250 mg g^{-1}<br>Selectivity for Mo(VI) in the presence of Cr(VI), As(V), V(VI)	
	MAA-*co*-EGDMA<br>Fe_3O_4/SiO_2	31.08	Batch adsorption<br>Conditions: 1 g L^{-1}; 3 M HCl; 298 K; 120 min<br>Fitted by Langmuir/PSO<br>Reusability: can be reused at least 5 times without significant loss of adsorption capacity.<br>Selectivity: k′ for Mo(VI) in the presence of Ni(II), Cd(II), Mn(II), Zn(II) and Cu(II) were 10.08, 7.11, 5.12, 11.31, and 7.88, respectively.	[160]
	ALG-cl-GA<br>With PVA as thickener	114.4	Batch adsorption<br>Conditions: 2 g L^{-1}; pH 3; 303 K; 72 h<br>Fitted by Langmuir/PSO<br>Reusability: reused up to three cycles.<br>Selectivity at pH 3: k′ = 2.43-3.71, for Re(VII), Cu(II), Fe(III), Zn(II) and Mn(II), and 47.16 for Mo(VI).	[161]
Re(VII)	I-EDA-CS	418.98	Batch adsorption<br>Conditions: 1 g L^{-1}; pH 3; 303 K<br>Fitted by Langmuir/PSO<br>Reusability: almost no loss of the sorption capacity after six cycles<br>Selectivity for Re(VII) in the presence of Cu(II), Zn(II), Fe(III), and Mn(II), at pH 3.0: 1.4, 1.51, 3.46, and 1.83.	[162]
	I-CTS-KIT-6	368.8	Batch adsorption<br>Conditions: 1 g L^{-1}; pH 3.0; 303 K<br>Fitted by Langmuir/PSO<br>Reusability: the sorbent preserved its capacity after six sorption-desorption cycles.<br>Selectivity: the adsorption efficiency for Zn(II), Cu(II), Mn(II) and Fe(III) in the presence of Re(VII) was < 5%.	[163]
Ru(III)	Ru(III)-BnTSn complex<br>4-VP-*co*-ST-*co*-DVB	0.237	Column adsorption<br>Conditions: pH 8,0; flow rate 0.2 mL min^{-1};<br>Selectivity in the presence of Pt(IV), Pd(II), Rh(II), Co(II) as competitors: k values in the range 5.4-12.5	[165]

	P(MMA-HEMA)<br>blended with<br>PVDF	42.31	Batch adsorption<br>Conditions: 1 g L^{-1}; pH 2.0; 298 K; 2 h<br>Fitted by Langmuir/PSO<br>Reusability: sorption capacity was 40.25 mg g^{-1} after 8 cycles<br>Selectivity: k′ was 4.14 in the presence of Ni(II) as interfering ion	[167]
	Ru-PTCS	249	Batch adsorption<br>Conditions: 1 g L^{-1}; pH 4; 303 K; 180 min<br>Fitted by Langmuir/PSO<br>Reusability: 96% of sorption capacity after 8 sorption-desorption cycles<br>Selectivity: in multicomponent systems with Pd(II), Pt(IV), Co(II), Fe(III), Ni(II), Cu(II), Mn(II), Zn(II), Ca(II) as competitors: k values in the range 5.47-12.37; k′ values > 6.	[168]
	Ru-IIP<br>PDEA-*b*-P(DEA-*co*-AM)<br>in<br>P(AM-*co*-MBAA)	15.46	Batch adsorption<br>Conditions: 5 g L^{-1}; pH 1.5; 308 K; 300 min<br>Fitted by Langmuir/PFO<br>Reusability: sorption capacity slightly decreased in the first 8 cycles, and significantly in the later three cycles.<br>Selectivity: *k′* values of Ru(III)/Ni(II), Ru(III)/Fe(III) and Ru(III)/Cu(II) systems were 3.102, 2.738 and 3.032.	[169]

Abbreviations: AAm - acrylamide; BnTSn - benzaldehyde thiosemicarbazone; cl - cross-linked; DEA - N,N-diethylacrylamide; DVB - divinylbenzene; EDA - ethylene diamine; ICTGBs - ion imprinted chitosan gel beads; MBAAm - N,N′-methylene-bis-acrylamide; MWCNTs - multiwalled carbon nanotube; PVA - poly(vinyl alcohol); PVDF - polyvinylidene fluoride; Ru-PTCS - 2-pyridylthiourea-chitosan imprinted with Ru(III); TEA - triethanol amine.

Table S9. Selective sorption of Ag(I), Au(III) and Pd(II) ions by IIP materials.

**Imprinted HMI**	**Polymeric material**	***q_m*, mg/g**	**Comments**	**Ref.**
Ag(I)	CS hydrogels	≈ 120	Batch adsorption;<br>Conditions: pH 5.6; 298 K; 4h;<br>Selectivity coefficient (k): Cu(II) = 4.21	[170]
	CS/TEA hydrogel beads	510	Batch adsorption;<br>Conditions: pH 5, 298 K, 1h for the CS/TEA hydrogel and 10h for the CS/TEA gel beads;<br>Selectivity coefficients (k):<br>CS/TEA hydrogel beads: Cu(II) = 3.72; Pb(II) = 28.56; Zn(II) = 30.64;	[173]
	CS/TEA gel beads	350		

			CS/TEA gel beads: Cu(II) = 3.95; Pb(II) = 39.4; Zn(II) = 37.9;<br>Reusability: 20 cycles	
	CS gel beads	89.2	Batch adsorption;<br>Conditions: pH 5, 298K, 48h;<br>Fitting: Langmuir/IPD;<br>Selective for Ag(I) over Cu(II);<br>Sorption of Ag(I) proceeds by ion exchange from pH 1 to pH 3, and by complexation from pH 3 to pH 5.	[171]
	CS-P(DVB-GMA-ST) particles	166	Batch adsorption;<br>Conditions: pH 5, 318 K, 8h;<br>Fitting: Langmuir/PSO;<br>Relative selectivity coefficients (k'): Cu(II) = 3.67-4.72, Zn(II) = 4.07-6.22	[172]
Au(III)	Ethylenediamine N-(2-(1-imidazolyl)ethyl-modified CS resin	810.67	Batch adsorption;<br>Conditions: pH 3;<br>Fitting: Langmuir/PSO;<br>Relative selectivity coefficients (k'):<br>Cu(II) = 4.263; Ni(II) = 3.405<br>Regenerated with 0.7 M TU – 2 M HCl solution;<br>Reusability: 5 cycles (95%).	[174]
	Poly(ethylene glycol) diacrylate cross-linked (4-Acryloylmorpholine)-*co*-(2-hydroxyethyl acrylate) hydrogel	78.43	Batch adsorption;<br>Conditions: pH 1, 3h;<br>Relative selectivity factors (α_r): Fe(III) = 50378, Hg(II) = 72588, Zn(II) = 40381.<br>Reusability: 5 cycles;<br>Regenerated with 0.8 M TU – 2 M HCl solution	[175]
	TU-functionalized ALG beads	184.82	Batch adsorption;<br>Conditions: pH < 2.36, 323 K, 6h;<br>Fitting: Freundlich/PFO, PSO;<br>Selective for Au(III) in mixture with Cu(II), Co(II), Ni(II) and Pb(II);<br>Reusability: 5 cycles;<br>UV light induced the photo-reduction of Au(III) ions to Au NPs.	[176]
	2-mercaptobenzaldehyde-CS sorbent		Batch adsorption;<br>Conditions: pH 4, 303 K, 1h;<br>Fitting: Langmuir;<br>Relative selectivity coefficients (k'): Cu(II) = 4.51, Ni(II) = 3.57, Cr(III) = 4.47, Pd(II) 5.13;<br>Regeneration with 0.5 M TU + 1 M H_2SO_4;<br>Reusability: 7 cycles (≈ 95%).	[177]
Pd(II)	CS fibers	150.3	Batch adsorption; Mixtures;<br>Conditions: pH 1, 298 K;	[178]

			Selective for Pd(II) in mixture with Co(II), Ni(II) and Pt(IV);<br>Reusability: 5 cycles	
	CS fibers	326.4	Batch adsorption;<br>Conditions: pH 1, 298 K, 1h, 0.4 g/L sorbent dose;<br>Fitting: Freundlich/PSO;<br>Selectivity coefficients (k): Pt(IV) = 2082 at pH 1, 29.12 at pH 2, and 5.48 at pH 3;<br>Reusability: 3 cycles (95.6%)	[179]
	CS fibers	112.4	Batch/Column adsorption; Mixture;<br>Conditions: pH 1, 298 K;<br>Selective for Pd(II) in mixture with Co(II), Ni(II), Cu(II) and Pt(IV);<br>Selective recovery of Pd(II) in packed columns from extremely acid (pH 0.2) hydrometallurgy wastewater.	[180]
	2-Aminobenzaldehyde-CS resin	275	Batch adsorption;<br>Conditions: pH 5, 303K, 1h, 1 g/L sorbent dose;<br>Fitting: Langmuir/PSO;<br>Relative selectivity coefficients (k'): Cu(II) = 1.97, Ni(II) = 1.88, Co(II) = 2.05, Mn(II) = 2.47;<br>Reusability: 5 cycles (96% recovery).	[181]

Abbreviations: TU – thiourea; P(DVB–GMA–St) – poly(divinylbenzene–glycidyl methacrylate–styrene); ECH – epichlorohydrin.

Table S10. Sorption of Ga(III), In(III) and Pb(II) ions by IIP materials.

Imprinted HMI	Polymeric material	q_m, mg/g	Comments	Ref.
Ga(III)	AA-*g*-CS bio-adsorbent	192.4	Batch adsorption;<br>Conditions: pH 3, 323 K;<br>Fitting: Langmuir/PSO;<br>Selective for Ga(III) in binary mixtures with Si(IV), Ge(III), Zn(II) or Al(III);<br>Reusability: 6 cycles (90.25%)	[204]
In(III)	PVPA-*g*-silica gel composite	45.07	Batch and column adsorption;<br>Conditions: pH 3, 298 K, 10 min;<br>Fitting: Langmuir/PSO;<br>Selectivity coefficients: In(III)/Cu(II) = 189.17, In(III)/Pb(II) = 67.94, In(III)/Zn(II) = 886.63, In(III)/FeII) = 2479.71.<br>Reusability: 6 cycles (in column);<br>Column adsorption capacity: 34.57 mg/g (flow rate = 0.5 mL/min);<br>Tested on real mining wastewater.	[205]

	P(VPA-AMT)-*g*-silica gel composite	60.62	Batch and column adsorption;<br>Conditions: pH 3, 318 K, 16 min;<br>Fitting: Langmuir/PSO;<br>Selectivity coefficients: In(III)/Cu(II) = 41, In(III)/Pb(II) = 212, In(III)/Zn(II) =37, In(III)/Fe(II) = 69;<br>Column adsorption capacity: 48.75 mg/g (flow rate = 0.5 mL/min);<br>Reusability: 6 cycles (in column);<br>Tested on real mining wastewater.	[206]
Pb(II)	Dithiocarbamate-CS beads	359.68	Batch adsorption;<br>Conditions: pH 6, 303 K, 3h;<br>Fitting: Langmuir/PSO;<br>Relative selectivity coefficients: Pb(II)/Cd(II) = 5.08, Pb(II)/Cu(II) = 2.45, Pb(II)/Ca(II) = 3.25, Pb(II)/Mg(II) = 4.24, Pb(II)/Zn(II) = 3.20;<br>Reusability: 10 cycles (75% recovery)	[210]
	TEPA-CS beads	259.68	Batch adsorption;<br>Conditions: pH6, 313K, 8h;<br>Fitting: Langmuir/PSO;<br>Relative selectivity coefficients: Pb(II)/Cd(II) 2.17, Pb(II)/Cu(II) = 5.08, Pb(II)/Ca(II) 2.18, Pb(II)/Mg(II) = 2.29, and Pb(II)/Zn(II) = 15.41;<br>Reusability: 10 cycles (84% recovery)	[211]
	P(MAA-EGDMA)-*g*-CS IPN hydrogel	37.5	Batch/column adsorption;<br>Conditions: pH 6, 2h;<br>Fitting: Langmuir/PSO;<br>Selectivity coefficients: Pb(II)/W(VI) = 161.58, Pb(II)/Zn(II) = 31.00, Pb(II)/Cu(II) = 39.12; Pb(II)/Mn(II) = 52.75; Pb(II)/K(I) = 77.58; Pb(II)/Mg(II) = 44.98;<br>Tested in column for selective extraction of Pb(II) from PCB recycling unit wastewater;<br>Reusability: 5 cycles (using PCB recycling unit wastewater).	[207]
	Fe_3O_4-CS biosorbent	69.48	Batch adsorption;<br>Conditions: pH 5, 8h;<br>Relative selectivity coefficients: Pb(II)/Cu(II) = 2.32, Pb(II)/Cd(II) = 2.2, Pb(II)/Ni(II) = 2.05;<br>Reusability: 5 cycles (87% recovery)	[213]

Abbreviations: PVPA – poly(vinylphosphonic acid); AMT – allyl mercaptan; TEPA – tetraethylenepentamine; PAA – poly(acrylic acid); PCB – printed circuit boards; IPN – interpenetrated polymer networks.
